# Evolution of diapause in the African turquoise killifish by remodeling the ancient gene regulatory landscape

**DOI:** 10.1016/j.cell.2024.04.048

**Published:** 2024-05-28

**Authors:** Param Priya Singh, G. Adam Reeves, Kévin Contrepois, Katharina Papsdorf, Jason W. Miklas, Mathew Ellenberger, Chi-Kuo Hu, Michael P. Snyder, Anne Brunet

**Affiliations:** 1 Department of Genetics, Stanford University, Stanford, CA, USA; 2 Stanford Cardiovascular Institute, Stanford University, Stanford, CA, USA; 3 Stanford Diabetes Research Center, Stanford University, Stanford, CA, USA; 4 Glenn Center for the Biology of Aging, Stanford University, Stanford, CA, USA; 5 Wu Tsai Neurosciences Institute, Stanford University, Stanford, CA, USA; 6 Chan Zuckerberg Biohub, San Francisco, San Francisco, CA, USA; 7 Present address: University of California, San Francisco and UCSF Bakar Aging Research Institute, San Francisco, CA, USA; 8 Present address: Institute of Molecular Biology (IMB), Mainz, Germany; 9 Present address: Department of Biochemistry and Cell Biology, Stony Brook University, Stony Brook, NY, USA; 10 These authors contributed equally; 11 Lead contact

## Abstract

Suspended animation states allow organisms to survive extreme environments. The African turquoise killifish has evolved diapause as a form of suspended development to survive a complete drought. However, the mechanisms underlying the evolution of extreme survival states are unknown. To understand diapause evolution, we performed integrative multi-omics (gene expression, chromatin accessibility, and lipidomics) in the embryos of multiple killifish species. We find that diapause evolved by a recent remodeling of regulatory elements at very ancient gene duplicates (paralogs) present in all vertebrates. CRISPR-Cas9-based perturbations identify the transcription factors REST/NRSF and FOXOs as critical for the diapause gene expression program, including genes involved in lipid metabolism. Indeed, diapause shows a distinct lipid profile, with an increase in triglycerides with very-long-chain fatty acids. Our work suggests a mechanism for the evolution of complex adaptations and offers strategies to promote long-term survival by activating suspended animation programs in other species.

## In brief

The African turquoise killifish has an extreme form of diapause that lasts many months, even years. Comparing gene expression and chromatin states across species revealed gene duplicates and a shift in lipid metabolism as important contributors to the specialization of a diapause expression program, critical for long-term protection and survival.

## Graphical abstract

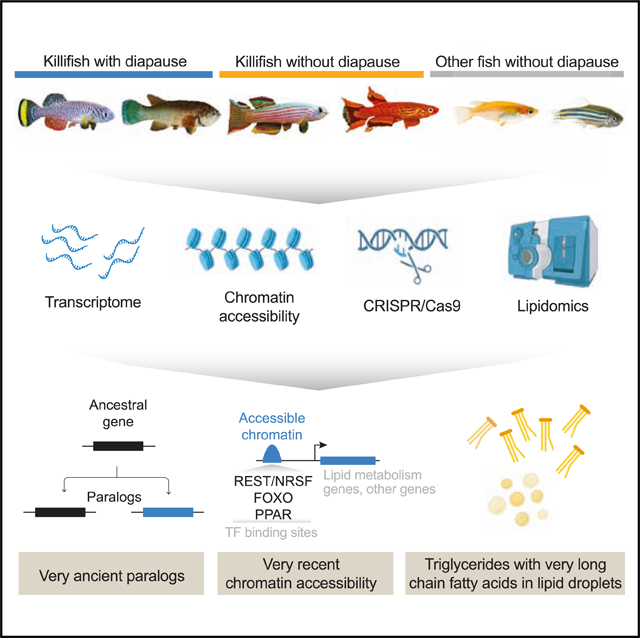

## INTRODUCTION

Extremophiles—species that live in extreme environments—have evolved unique adaptations for survival. Understanding how extreme adaptations evolve can reveal new pathways with important ramifications for survival in all organisms. The African turquoise killifish *Nothobranchius furzeri* is an extremophile for embryo survival. This vertebrate species lives in ephemeral ponds in Zimbabwe and Mozambique that completely dry up for ~8 months each year.^1^ To survive this annual drought, the African turquoise killifish has evolved two key adaptations: a rapid life to successfully reproduce during the rainy season and a form of long suspended animation, with embryos entering diapause and subsisting in the mud during the dry season.^2–5^ Diapause embryos survive for months, even years—longer than adult life—without any detectable tradeoff for future life.^6^ Remarkably, diapause embryos already have complex organs and tissues, including a developing brain and heart.^6^ Hence, diapause provides long-term protection to a complex organism.

Like other suspended animation states (hibernation, torpor), diapause is a multifaceted and active adaptation. Diapause also exists in other vertebrate species, including mammals (e.g., bear, roe deer, and mice).^7^ However, the African turquoise killifish represents an extreme case of diapause in terms of duration and complexity and provides a model to understand the mechanism and evolution of this suspended animation trait in vertebrates. Many genes involved in chromatin remodeling, metabolism, and stress resistance are upregulated in killifish diapause.^6,8,9^ Yet, how such an extreme and coordinated program evolved in nature is unknown. Using the lens of evolution to understand diapause could uncover new protective mechanisms for long-term survival and offer a framework for the evolution of extreme adaptations in nature.

## RESULTS

### Paralogs that specialize for expression in diapause are evolutionarily very ancient

**Figure 1.**
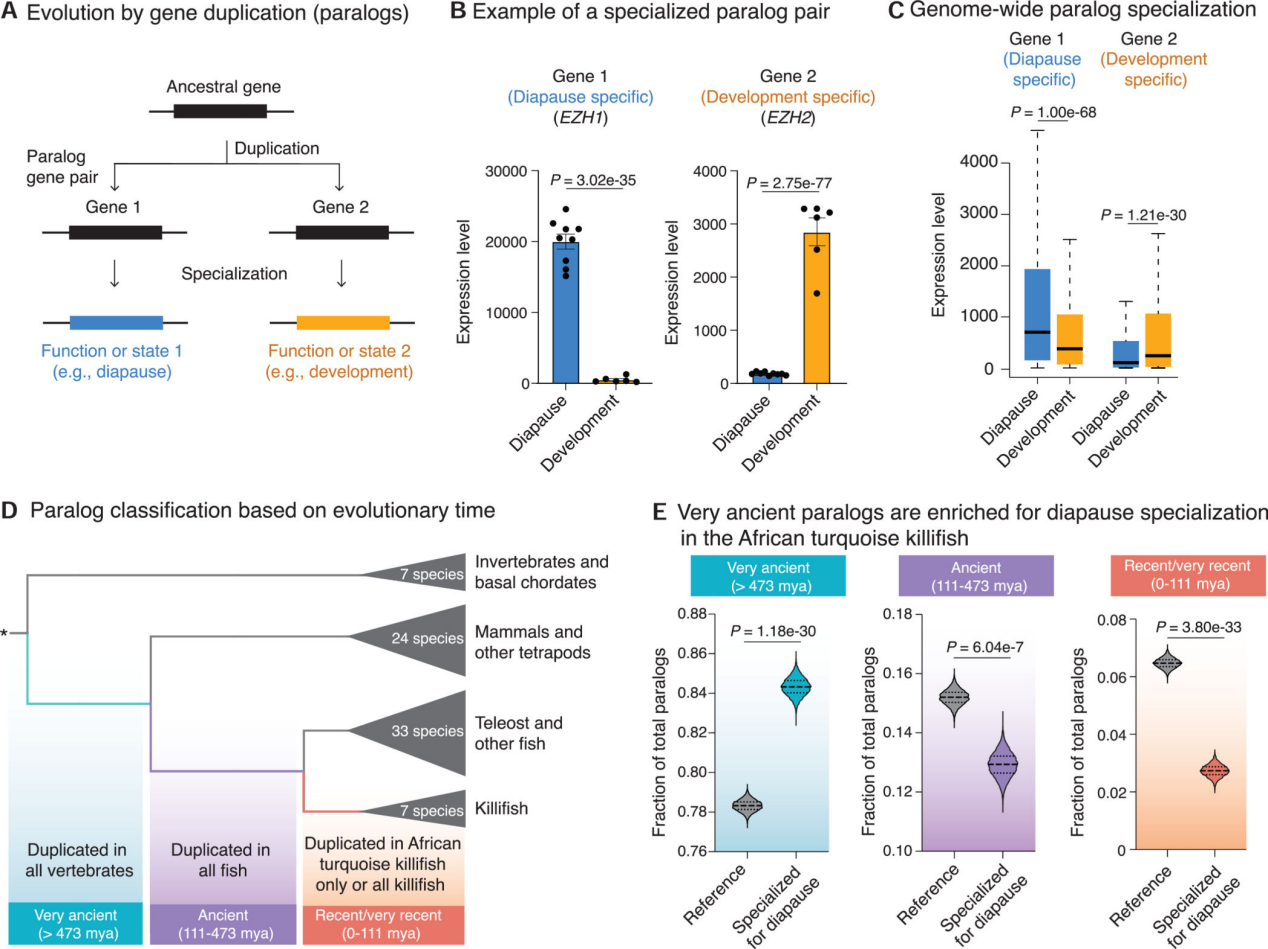
Specialization of very ancient paralogs for expression in diapause in the African turquoise killifish (A) Schematic of paralog specialization after gene duplication. After duplication from the same ancestral gene, genes from a paralog gene pair can specialize for different functions or states (e.g., diapause vs. development). (B) Examples of a paralog gene pair, with specialized expression of gene 1 in diapause (blue, *EZH1*) and gene 2 in development (orange, *EZH2*) in the African turquoise killifish. Bars represent mean expression level (normalized DESeq2 count) across replicates in diapause or development state. Dots show normalized DESeq2 counts in each replicate. Error bar is standard error of mean. Corrected *p* values (median from pairwise comparisons) from DESeq2 Wald test. (C) Boxplots showing expression levels (normalized DESeq2 counts) of all the specialized paralog pairs in diapause and development in the African turquoise killifish genome. Gene 1 of the paralog pair has a higher expression on average in diapause (blue) compared with development (orange), whereas gene 2 hasa higher expression on average in development (orange) compared with diapause (blue). *p* values from Kolmogorov-Smirnov test. (D) Schematic for binning paralog duplication time into 3 categories based on OrthoFinder pipeline with 71 species (see STAR Methods). Divergence time estimates are from Ensembl species tree. Binned categories include genes that were duplicated in the common ancestor of (1) all vertebrates or earlier (very ancient, >473 million years ago [mya]), (2) all fish (ancient, 111–473 mya), and (3) all killifish or the African turquoise killifish exclusively (recent or very recent, 0–111 mya). (E) Fraction of total paralog pairs within each of the very ancient (left), ancient (middle), and recent/very recent (right) binned categories. Violin plots represent the distribution of observed vs. expected specialized paralog fractions generated through 10,000 bootstrapped random sampling. Median and quartiles are indicated by dashed lines. The enrichment of diapause-specialized paralog pairs within each bin is compared with genome-wide expectation. *p* values from chi-square test. See also Figure S1.

We asked when, in evolutionary time, the genes expressed in diapause originated. To this end, we focused on paralogs—duplicated copies of genes.^10,11^ Paralogs are the primary mechanism by which new genes originate and specialize for new functions or states^12,13^ (Figure 1A). Paralogs also allow for a precise timing of the evolutionary origin of specific genes, and they could help explain how the suspended animation state of diapause evolved in the killifish genome. In the African turquoise killifish genome, similar to other vertebrates, most genes have at least one paralog partner. Using phylogenetic inference, we identified 20,091 paralog pairs in the African turquoise killifish genome for our analyses (see STAR Methods and Table S1C). We used our previously generated RNA sequencing (RNA-seq) datasets of development and diapause in the African turquoise killifish^6^ to analyze if the expression pattern of paralogs has diverged in diapause vs. normal development states. Interestingly, many paralog pairs show opposing expression, with one gene in the paralog pair highly expressed in diapause (“diapause-specialized gene,” e.g., the chromatin modifier *EZH1*) and the other gene in the paralog pair highly expressed in development (“development-specialized gene,” e.g., the chromatin modifier *EZH2*) (Figures 1B and S1A–S1C). Overall, 6,247 paralog pairs show expression specialization in diapause (Figure 1C; Table S1C).

We next asked whether paralogs that exhibit expression specialization in diapause are evolutionarily recent or ancient. Diapause in the African turquoise killifish is a relatively recent specialization that evolved less than 18 million years ago (mya).^14^ To determine a time frame of paralogs duplication, we generated a paralog classification pipeline to identify the evolutionary time when each of the African turquoise killifish paralogs originate compared with other species (STAR Methods; Figure 1D).^15^ We distinguished (1) very ancient paralogs (shared with other vertebrates, including mammals) that originated more than 473 mya, (2) ancient paralogs (shared with other fish) that originated between ~111 and 473 mya, and (3) recent/very recent paralogs (killifish/African turquoise killifish specific) that originated less than ~111 mya (Figure 1D; Table S1C). Surprisingly, very ancient paralogs were significantly more likely to specialize for diapause compared with the genome-wide average, even though diapause originated recently (Figure 1E). By contrast, ancient and recent/very recent (killifish-specific) paralogs were significantly less likely to specialize for diapause compared the genome-wide average (Figure 1E). The enrichment for very ancient paralog pairs for specialization in diapause was qualitatively robust to varying outgroups, phylogeny, method to identify paralogs, and paralog family size (Figure S1D; Table S1D). Such an enrichment was not observed for paralogs that are expressed at the same level during development and diapause (in fact, those exhibited a decrease for very ancient paralogs) (Figure S1E) nor in randomized paralog pairs (Figure S1F). Hence, paralogs that exhibit expression specialization in diapause are evolutionarily very ancient.

We next assessed the genomic properties of the paralog pairs specialized for diapause. The majority of paralog pairs are chromosomal duplications in killifish, likely due to whole-genome duplications in the ancestors of all teleost fish, and only a minority are tandem duplicates (on the same chromosome) (Figure S1G). Paralog pairs on separate chromosomes were more likely to specialize for diapause, whereas tandem duplicates were less likely to specialize for diapause (Figures S1G and S1H). These findings are consistent with the observation that duplicates on separate chromosomes acquire different regulatory landscape, while tandem duplicates tend to be co-regulated.^16^ Paralogs that specialize for diapause did not exhibit an increased positive selection at the protein level (Figure S1I) and were in fact more conserved at the gene level than the genome-average (Figure S1J), perhaps reflecting the critical roles of these paralogs, in general, in these states. These results suggest that conserved very ancient paralogs are co-opted for the suspended animation state of diapause, likely by remodeling of their regulatory landscape.

### Very ancient paralogs also specialize in diapause in other killifish species and in mouse

**Figure 2.**
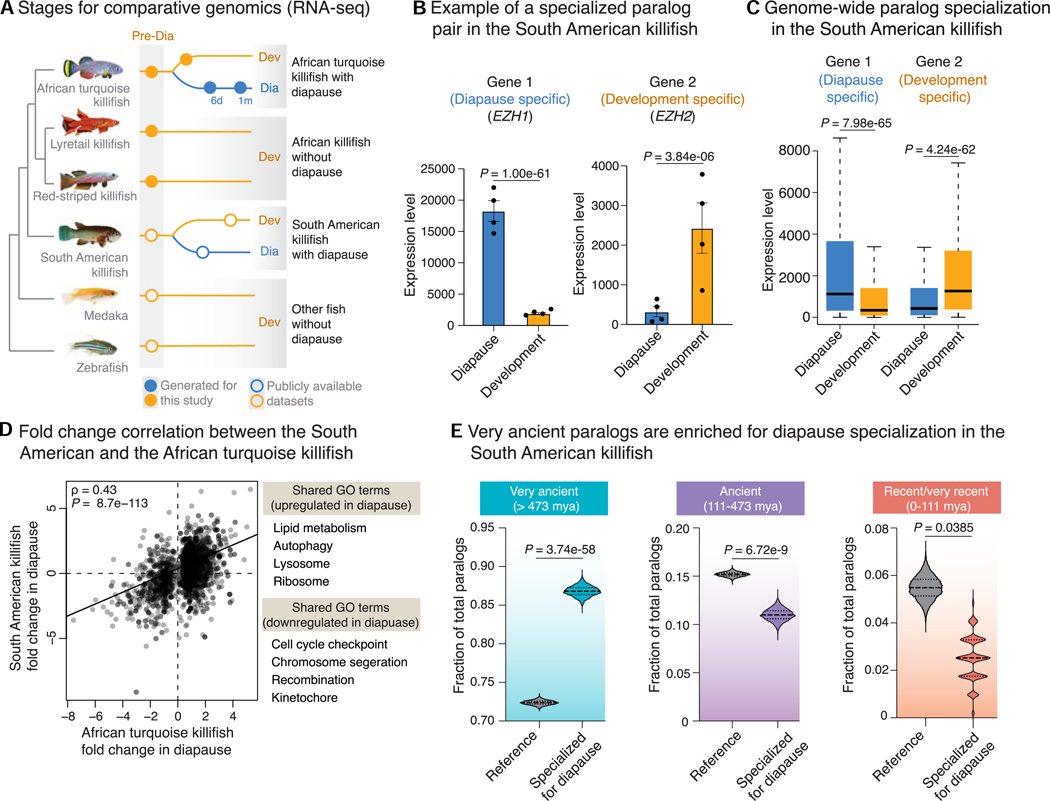
Very ancient paralogs also specialize for expression in diapause in other killifish species with diapause (A) Experimental design for analysis of RNA-seq datasets either publicly available (hollow circles) or *de novo* generated for this study (filled circles) (see also Table S1B). Killifish species are from Africa (with and without diapause) and South America (with diapause). Medaka and zebrafish are other teleost fish without diapause. The development stage (orange hollow circle) in the South American killifish corresponds to post-diapause development. Pre-Dia, pre-diapause; Dia, diapause; Dev, development; 6 days, 6 days in diapause; 1 month, 1 month in diapause. (B) Examples of paralog gene pair, with specialized expression of gene 1 in diapause (blue, *EZH1*) and gene 2 in development (orange, *EZH2*) in the South American killifish. Gene names displayed are the names assigned to the ortholog in the African turquoise killifish. Bars represent the mean expression level (normalized DESeq2 count) across replicates in diapause or post-diapause development state. Error bar is standard error of mean. Each dot represents the normalized expression level for all sample replicates in diapause or post-diapause development. *p* values from DESeq2 Wald test. (C) Boxplots showing the expression levels (normalized DESeq2 counts) of all the specialized paralog pairs in diapause and development in the South American killifish. Gene 1 of the paralog pair has a higher expression on average in diapause (blue) compared with development (orange), whereas gene 2 has a higher expression on average in development (orange) compared with diapause (blue). *p* values from Kolmogorov-Smirnov test. (D) Spearman’s rank correlation between ortholog genes that change with diapause in African turquoise killifish and South American killifish. Dots represent the fold change values of ortholog genes in diapause compared with development in the two species. Spearman’s correlation coefficient (r) and *p* values are indicated. Selected Gene Ontology (GO) terms shared between both species are listed on the right (see also Figure S2I and Table S2A). (E) Fraction of the total paralog pairs within each of the very ancient (left), ancient (middle), and recent/very recent (right) binned categories as described in Figure 1D. Violin plots represent the distribution of observed vs. expected specialized paralog fractions generated through 10,000 bootstrapped random sampling. Median and quartiles are indicated by dashed lines. The enrichment of diapause-specialized paralog pairs within each bin is compared with genome-wide expectation (reference). *p* values are from chi-square test. See also Figure S2.

Many killifish species populate the world, and their ability to undergo diapause is linked to their environment. Killifish species that live in ephemeral ponds exhibit diapause (e.g., African turquoise killifish, South American killifish), whereas killifish species that live in constant water do not undergo diapause and instead continuously develop (e.g., red-striped killifish and lyretail killifish)^17–20^ (Figure 2A). To assess whether the specialization of ancient paralogs in diapause is generalizable to other species that evolved diapause independently, we used RNA-seq data from diapause and development in the South American killifish with diapause, *Austrofundulus limnaeus*,^9^ focusing on samples with time points similar to our study (Figure S2A). We also generated RNA-seq data from the developing embryos of the redstriped killifish *Aphyosemion striatum* and the lyretail killifish *Aphyosemion australe*—the closest relatives of the African turquoise killifish *N. furzeri* but without diapause (Figure 2A). In the South American killifish, paralogs also showed specialized expression in diapause vs. development (Figures 2B, 2C, and S2B–S2D), and their specialized expression correlated with that of paralogs in the African turquoise killifish (Figures 2D and S2E). By contrast, killifish species without diapause expressed both paralogs during development (Figure S2F). Importantly, paralogs with specialized expression in diapause in the South American killifish were likewise enriched for very ancient gene duplicates (Figure 2E).

Mammalian species have forms of embryonic diapause that can last from weeks to a few months.^21^ To assess if specialization and repurposing of very ancient paralogs are also observed in mammals, we analyzed paralog expression and specialization in embryonic diapause in the house mouse, *Mus musculus*.^22^ Expression of genes in the African turquoise killifish and mouse diapause was significantly correlated (Figure S2G), and mouse paralogs specialized in diapause were also very ancient (shared by other vertebrates) (Figure S2H). Genes upregulated in diapause across species shared many functions, including lipid metabolism (Figures 2D and S2I; Table S2A). These data suggest that the specialization and repurposing of very ancient paralogs is an evolutionary mechanism that is repeatedly employed for the evolution of diapause states across distantly related species.

We asked if some genes are uniquely regulated in African turquoise killifish diapause but not in South American killifish or mouse diapause. We identified 2,430 genes that are uniquely regulated in the African turquoise killifish diapause (Figure S2J). Genes uniquely regulated in diapause in the African turquoise killifish were also very ancient paralogs (Table S1D) and were enriched for functions related to ribosome, translation, RNA processing, mitochondria, and peroxisomes (Table S2B), potentially highlighting pathways involved in extreme forms of diapause.

Collectively, these results indicate that very ancient paralog pairs have been repeatedly co-opted for specialized expression in diapause during evolution.

### Evolutionarily recent remodeling of the chromatin landscape at very ancient paralogs

**Figure 3.**
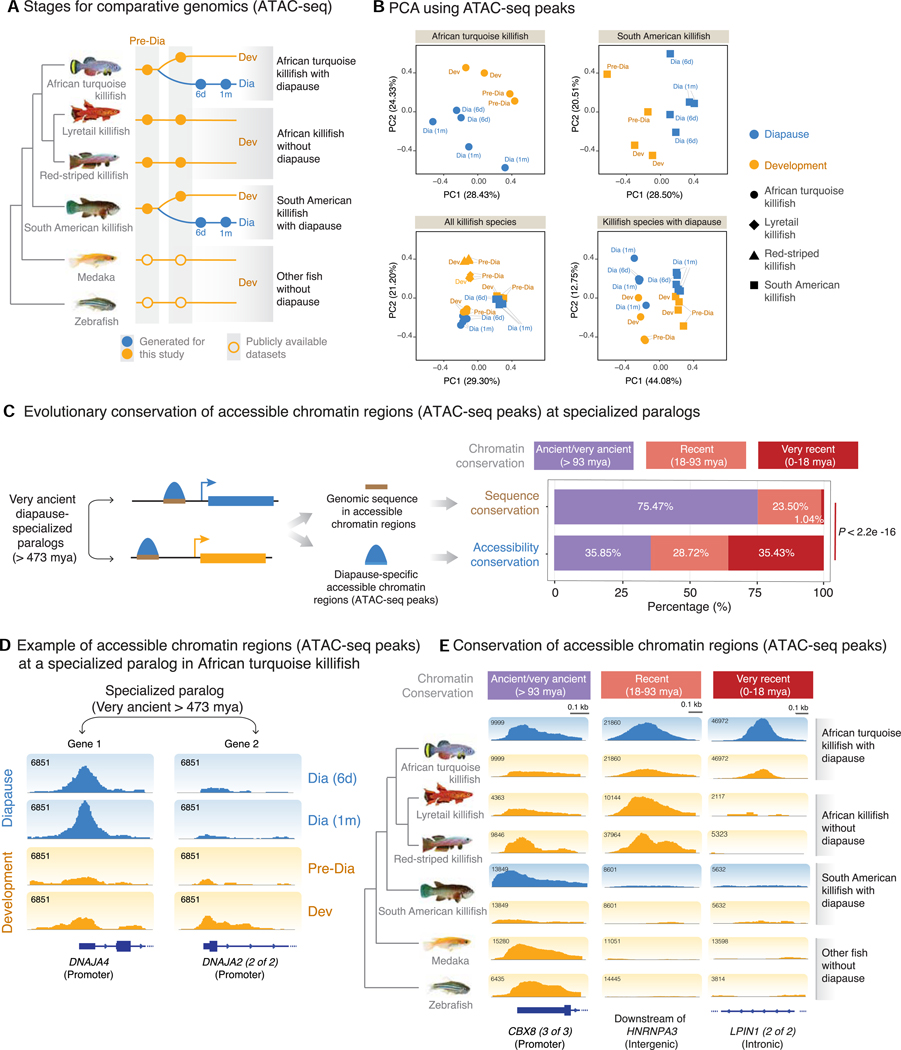
Evolutionarily recent remodeling of genome-wide chromatin landscape drives specialized expression of very ancient paralogs in diapause (A) Experimental design for the ATAC-seq datasets either publicly available (hollow circles) or *de novo* generated in this study (filled circles) (see also Table S1B). Pre-Dia, pre-diapause; Dia, diapause; Dev, development; 6 days, 6 days in diapause; 1 month, 1 month in diapause. (B) Principal-component analysis (PCA) on all chromatin accessibility regions in each species group: African turquoise killifish only (upper left), South American killifish only (upper right), all killifish species (lower left), and diapause-capable killifish (lower right). Each point represents the consensus ATAC-seq peaks (chromatin accessibility) from an individual replicate of a given species at a given developmental or diapause state. Percentage of the variance explained by each principal component (PC) is shown in parentheses. (C) Conservation analysis of genomic sequence and chromatin accessibility at very ancient paralogs with specialization in diapause vs. development. Left: schematic of the analysis. Right: percentage (e.g., conservation) of alignable regions containing diapause-specific chromatin accessibility (upper) and the conservation of diapause-specific chromatin accessibility (lower) near specialized ancient paralogs (see also Figures S3C–S3E). Although the majority of genomic sequences are ancient, the chromatin accessibility at those peaks evolved recently in the African turquoise killifish. *p* values from chi-square test. (D) Example of a chromatin accessibility regions (ATAC-seq peaks) at the promoter of genes from a very ancient paralog pair, with one gene specialized for expression in diapause (*DNAJA4*) and the other in development (*DNAJA2*). Replicates within each condition were aggregated by summation for visualization. Blue boxes and lines represent genomic features (exons and introns, respectively). (E) Examples of diapause-specific chromatin accessibility regions that are: ancient/very ancient (conserved across killifish and medaka or zebrafish; left), recent (conserved in at least 2 killifish species; middle), or very recent (specific to African turquoise killifish; right). Blue boxes and lines at the bottom represent genomic features for the closest gene (exons and introns, respectively). To generate tracks, reads per kilobase per million mapped reads (RPKM)-normalized reads were summed across replicates and biological time points (e.g., diapause and development separately) to obtain single tracks for each species. See also Figure S3.

To characterize the regulatory landscape of the paralogs that specialize in diapause during evolution, we profiled the chromatin accessibility landscape in different species of killifish. We performed assay for transposase-accessible chromatin using sequencing (ATAC-seq), which assesses chromatin accessibility genome wide,^23^ on embryos during diapause and development in killifish species with diapause (African turquoise killifish, South American killifish) and embryos during development in killifish species without diapause (lyretail killifish and red-striped killifish) at a similar developmental stage (Figure 3A). We also used available ATAC-seq data for medaka and zebrafish development at a similar developmental stage.^24^ We verified the quality of our ATAC-seq samples by quality control metrics recommended by the ENCODE consortium (see STAR Methods, Figures S3F and S3G, and Table S3A).

Diapause and development embryos have a distinct chromatin accessibility landscape genome wide, as shown by principal-component analysis (PCA) in the African turquoise killifish and South American killifish (Figure 3B). Accessible chromatin regions also separated diapause and developmental samples of different killifish species (Figure 3B). In the African turquoise killifish, 6,490 genomic regions were differentially accessible in diapause compared with development genome wide (Figure S3A; Table S3B), and they were located mostly in promoter, intronic, or distal intergenic (e.g., enhancer) regions (Figure S3B).

We next examined accessible chromatin regions (ATAC-seq peaks) at paralogs that are differentially expressed in diapause vs. development (e.g., *DNAJA4* and *DNAJA2*; Figures 3D, S1B, and S2C). Paralogs that are specialized in diapause are very ancient (>473 mya); we therefore asked when did the chromatin at these paralogs become accessible (Figure 3C). We developed a pipeline to identify the relative evolutionary origin of ATAC-seq peaks based on multi-genome alignment (see STAR Methods) and classified each ATAC-seq peak as (1) ancient/very ancient (i.e., chromatin accessible in all fish species evaluated, such as *CBX8*), (2) recent (i.e., chromatin accessible only in killifish species, such as *HNRNPA3*), and very recent (chromatin accessible only in the African turquoise killifish, such as *LPIN1*) (Figure 3E). Interestingly, most regulatory regions of very ancient paralogs (>473 mya) that are differentially regulated in diapause exhibited chromatin accessibility very recently (~18 mya), only in the African turquoise killifish (Figure 3C). The very recent chromatin accessibility at very ancient paralogs specialized in diapause was generalizable to non-paralog genes (Figures S3C and S3D) and was most pronounced at distal regulatory elements (likely enhancers) (Figure S3E). Thus, the African turquoise killifish exhibits an evolutionary recent remodeling of the chromatin accessibility landscape at very ancient genes.

### Transcriptional regulators underlying chromatin accessibility in diapause

**Figure 4.**
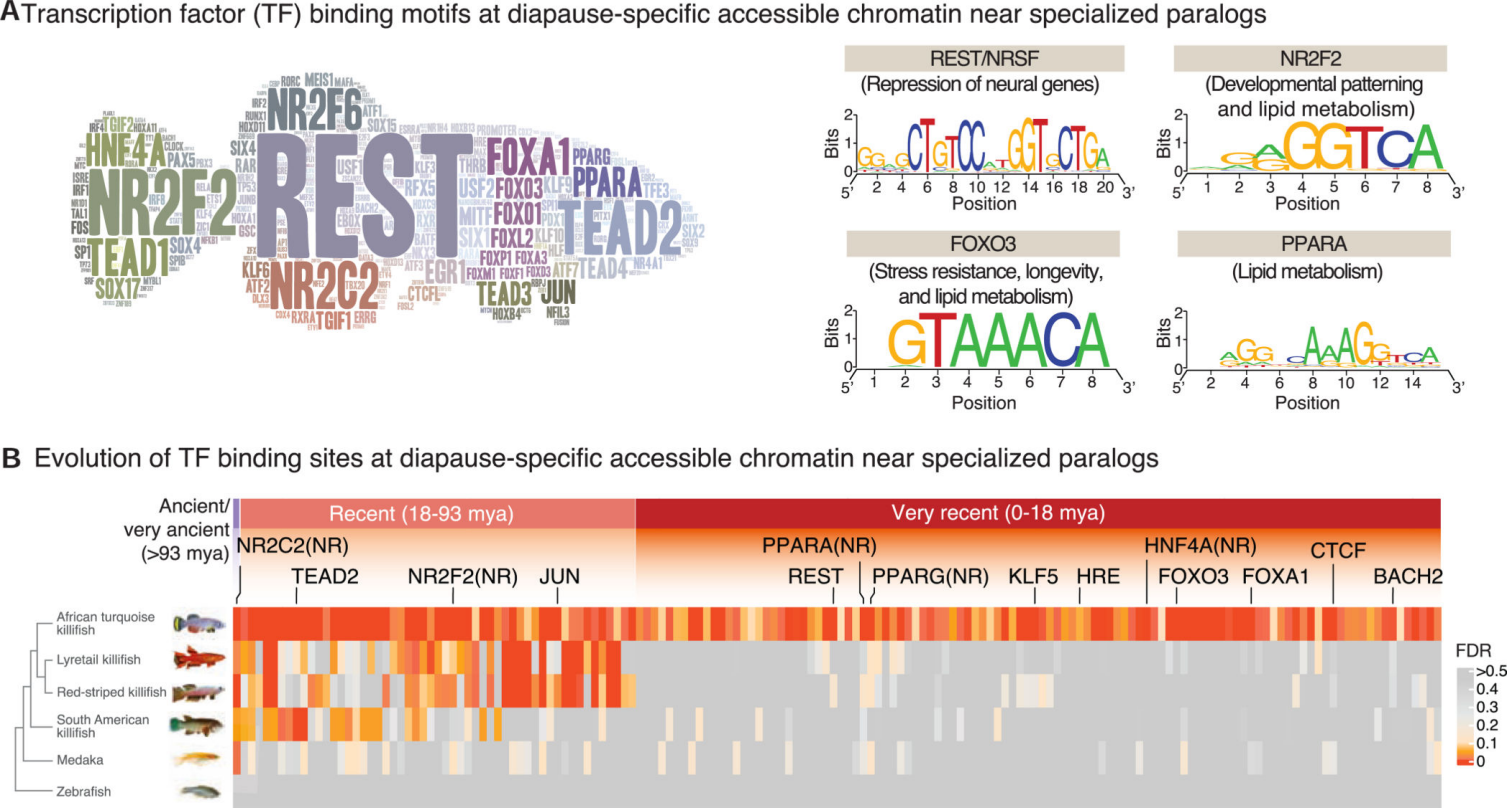
Transcription factor binding site enrichment and conservation within diapause-accessible chromatin (A) Left: word cloud for transcription factors whose binding sites are enriched in the diapause-specific chromatin-accessible regions (ATAC-seq peaks) at specialized paralogs using hypergeometric optimization of motif enrichment (HOMER). Right: consensus binding sites for selected transcription factors. y axis represents *informational content* (i.e., bits), which scales based on single-base overrepresentation in the binding sequence (0: bases are represented equally at 25% each in reference sequences; 2: a single base dominates the entirety of reference sequences at 100%). (B) Conservation in other fish species of the transcription-factor binding sites enriched in the diapause-specific chromatin-accessible regions in the African turquoise killifish. The majority of diapause-specific sites are very recent (i.e., specific to the African turquoise killifish) and not enriched in killifish species without diapause. Selected representative motifs are highlighted. See also Figure S4.

What are the transcriptional regulators underlying evolutionarily recent chromatin accessibility in diapause? We performed enrichment analysis at specialized paralogs (Figure 4; see Figure S4A for paralogs and singletons). Chromatin regions that opened recently in diapause paralogs in the African turquoise killifish were enriched for transcription factor (TF) binding sites for restrictive element-1 silencing transcription factor (REST)/neuron-restrictive silencing factor (NRSF) (hereafter REST), nuclear receptor subfamily 2 group F member 2 (NR2F2), forkhead TFs (e.g., forkhead box A1 (FOXA1) and forkhead box O3 (FOXO3), peroxisome proliferator-activated receptor (PPAR) (e.g., PPARA), and others (Figure 4A). These TF binding sites were specifically enriched in diapause-accessible chromatin but not in development-accessible chromatin (Figure S4B).

Interestingly, TF binding sites for REST, FOXO, and PPARA were enriched in chromatin regions that opened recently in the African turquoise killifish but were not enriched, at the same genomic location, in fish species without diapause (Figure 4B). This differential enrichment was not observed at conserved, accessible chromatin regions genome wide (Figure S4C). Thus, these TF binding sites arose very recently in the African turquoise killifish after divergence from other African killifish species without diapause and could underlie the expression specialization of paralogs in diapause.

The African turquoise killifish and South American killifish have evolved diapause independently,^18^ raising the possibility of either independent or convergent evolution. TF binding sites for REST, FOXO, and PPAR were not enriched, at the same genomic location, in the South American killifish (Figure 4B). However, an alignment-independent analysis at regulatory regions revealed that similar TF binding sites (e.g., REST, FOXO, and PPAR binding sites) were enriched in diapause-specific accessible chromatin at specialized paralogs in the South American killifish (Figure S4D; Table S3C). With this alignment-independent analysis, TF binding sites enriched in the African turquoise killifish and South American killifish, which both have diapause, indeed clustered more readily together than in species without diapause (Figure S4E). These observations are consistent with the convergent evolution of diapause in African turquoise killifish and South American killifish.

Overall, these results identified key TFs underlying diapause regulation, which evolved recently in the African turquoise killifish.

### Evolutionary mechanisms for the origin of TF binding sites in diapause

**Figure 5.**
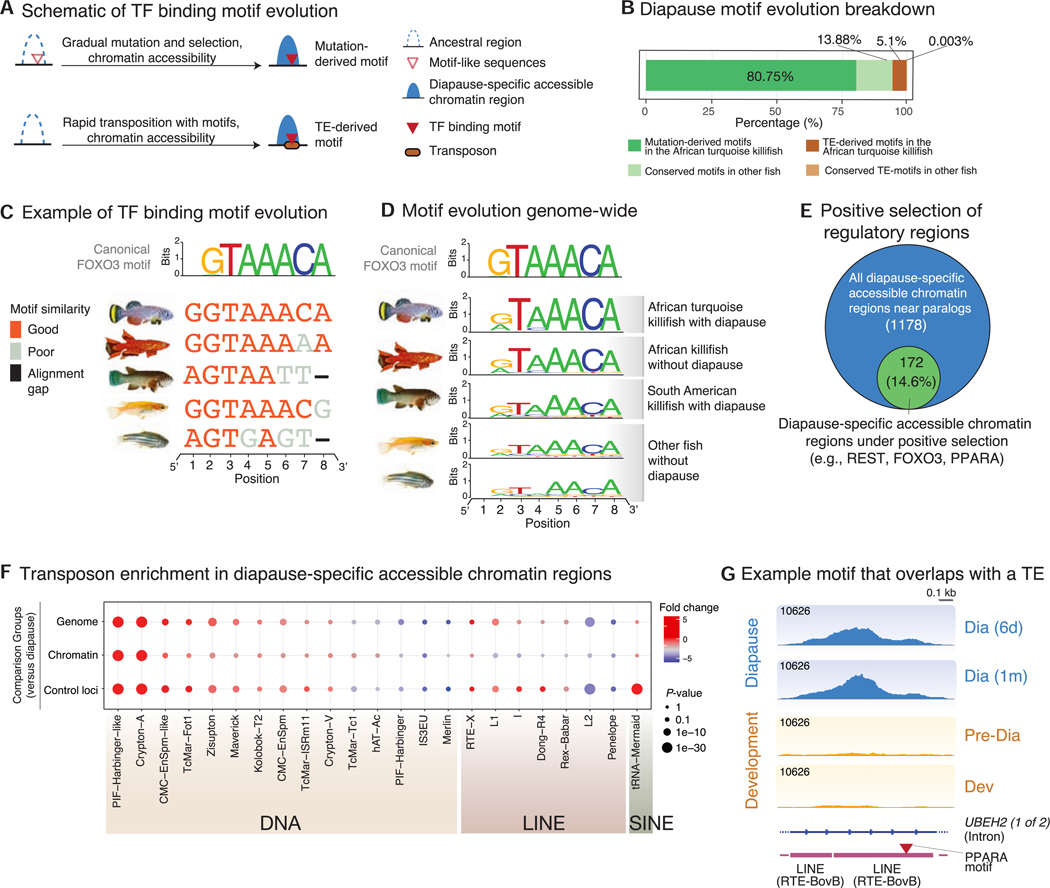
Mechanisms underlying the evolution of chromatin accessibility in diapause at specialized paralogs (A) Schematic of two possible mechanisms for the evolution of the diapause-specific transcription factor binding sites in the African turquoise killifish genome. Upper: gradual mutations paired with selective pressure lead to the formation of binding sites for specific transcription factors and accompanying chromatin accessibility. Lower: a site experiences a transposable element (TE) insertion event, providing a novel sequence that contains a binding site for a specific transcription factor and accompanying chromatin accessibility. (B) Percentage contribution of the two possible evolutionary mechanisms (mutation and TE insertion) for motif evolution in diapause-specific chromatin at specialized paralogs. Mutation-derived or TE-derived motifs are subdivided for their conservation either only in the African turquoise killifish (dark colors) or in at least one other species (light colors). Binding expectations are based on the HOMER log odds ratio binding criteria (see STAR Methods). The largest fraction of diapause-specific binding sites likely evolved through mutations exclusively in the African turquoise killifish. (C) Example of a transcription factor binding site that likely evolved via mutation: FOXO3 binding site in a diapause-specific chromatin-accessible region of the African turquoise killifish genome near *GPC3* (*LOC107379575*) and aligned regions in other fish species. Aligned sequences are colored based on the similarity to the canonical FOXO3 binding site from HOMER (top track). (D) Aggregated informational content across all FOXO3 transcription factor binding sites in diapause-specific accessible chromatin regions and aligned regions in other species. y axis is formatted using *informational content* (i.e., bits). The African turquoise killifish motif exhibits highest similarity to the canonical FOXO3 binding site. (E) Fraction of diapause-specific chromatin-accessible regions under positive selection in the African turquoise killifish (see STAR Methods) at false discovery rate (FDR) < 0.1. These regions are enriched for many TF binding sites (e.g., REST, FOXO3, and PPARA). (F) Enrichment or depletion of specific transposable elements (TEs) in the diapause-specific chromatin-accessible regions (ATAC-seq peaks) in the African turquoise killifish genome as compared with three sets of reference regions: the overall genomic abundance of the given TE (“Genome”), the abundance of the given TE in all chromatin-accessible regions (“Chromatin”), and the abundance of the given TE in size-matched control regions 10 kb away from ATAC-seq peak of interest (“Control loci”). (G) Example of a diapause-specific chromatin accessibility region containing a PPARA binding site overlapping with a TE. Almost all TE-derived motifs are specific to the African turquoise killifish. See also Figure S5.

Binding sites for TFs can arise *de novo* by point mutation or transposable element (TE) insertion^25,26^ (Figure 5A). A majority (81%) of the TF binding sites associated with diapause-specific accessible chromatin at specialized paralogs in the African turquoise killifish evolved *de novo* via mutation of the ancestral sequence (Figure 5B). For example, TF binding sites (e.g., FOXO3 motifs) were canonical binding sites (as defined by hypergeometric optimization of motif enrichment [HOMER]^27^) in the African turquoise killifish sequence but were slightly divergent in closely related fish without diapause and more divergent or even absent in more distant fish species (Figures 5C, 5D, S5A, and S5B). Importantly, we found a signature of positive selection^28^ at many of the diapause-specific accessible chromatin regions in the African turquoise killifish, including those in *cis* with specialized paralogs. These sites included enrichment for binding sites for REST, FOXO3, and PPAR (Figures 5E and S6A–S6C) and functions related to lipid metabolism and storage (Table S4A). Thus, the African turquoise killifish may have selected for canonical TF binding sites at regulatory regions of genes beneficial for diapause.

Intriguingly, 5% of TF binding sites associated with diapause in the African turquoise killifish paralogs overlapped with TEs and were unique to this species (Figure 5B). TEs can deliver TF binding sites to new regulatory neighborhoods faster than gradual mutation and selection, and they have exploded in the African turquoise killifish genome.^29^ They may represent a rapid evolutionary mechanism to co-opt genes into the diapause expression program. Several TE families (e.g., DNA transposons and long interspersed nuclear elements [LINEs]) were highly enriched at accessible chromatin regions in diapause in the African turquoise killifish (Figure 5F) and in some cases contained both a TE and a TF binding site (Figure 5G). Hence, TF binding sites underlying diapause-specialized paralogs have primarily originated through mutation and selection with some contribution from a recent burst of transposon-mediated reshuffling in the African turquoise killifish.

### A CRISPR-Cas9-based platform identifies functional regulators of the diapause program

**Figure 6.**
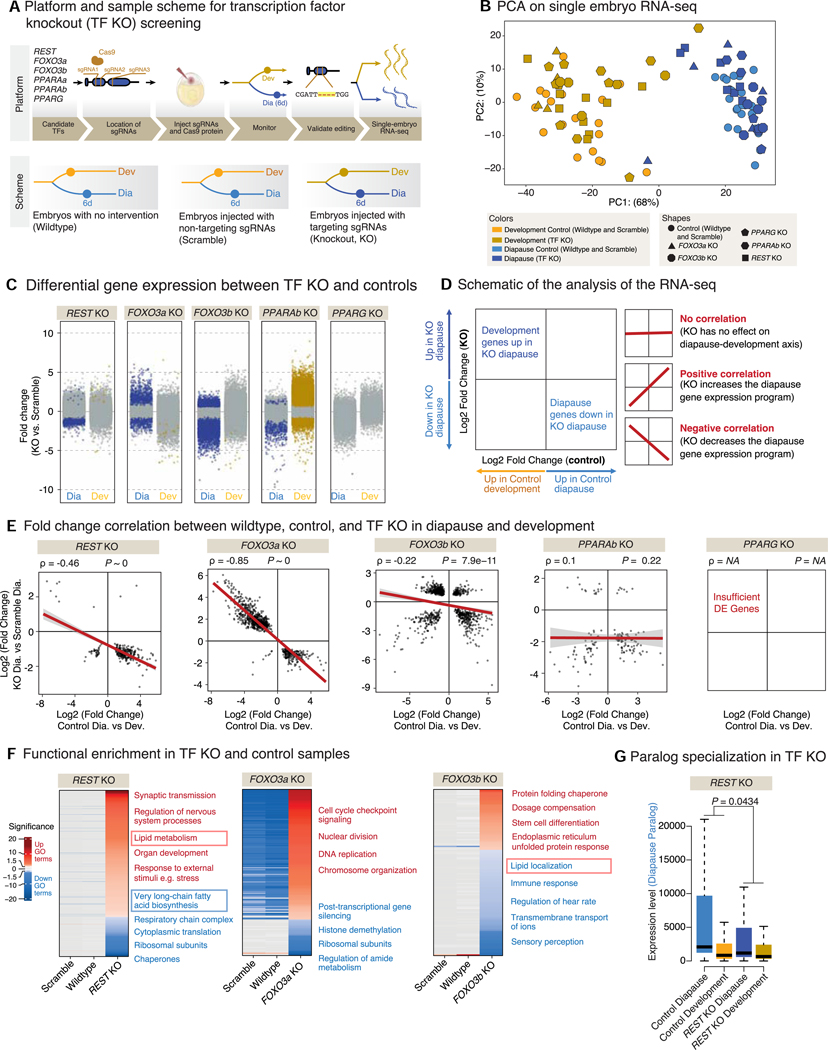
Functional assessment of key transcription factor knockouts on the diapause gene expression program (A) CRISPR-Cas9-based platform to assess the effect of transcription factor knockout on diapause and development gene expression programs in injected (F0) embryos in the African turquoise killifish (upper). Scheme of the stages for RNA-seq analysis (lower). Six transcription factors (TFs) were selected: REST, FOXO3a, FOXO3b, PPARAa, PPARAb, and PPARG. CRISPR-Cas9-mediated knockouts were conducted by injecting 3 single guide (sgRNAs) per gene in single-cell embryos. Non-injected embryos (wild type) and embryos injected with scrambled sgRNAs (scramble) were used as controls. Genotyping and RNA-seq on individual embryos were performed at two stages: diapause (Dia, blue, 6 days) or development (Dev, orange). A total of 130 single-embryo RNA-seq libraries were generated and analyzed. (B) Principal-component analysis (PCA) of transcription factor knockouts and control RNA-seq samples in the African turquoise killifish. Each shape represents transcriptome of a single embryo. Knockouts and wild-type/scramble controls are denoted by different shapes. *PPARAa* knockout was lethal, and no viable embryos were recovered. Color denotes diapause (blue) or development (orange) for each embryo. Percentage of variance explained by each principal component (PC) is shown in parentheses. (C) Fold changes of differentially expressed genes (DEGs) between diapause and development for each transcription factor knockout compared with scrambled sgRNAs-injected embryos (scramble). Each dot represents a single gene. Significantly differentially expressed genes (DEseq2, FDR < 0.1) are in color: diapause (dark blue) or development (dark orange). Genes not significantly changed are in gray. *PPARAa* knockout was embryonic lethal, and no viable embryos were recovered. (D) Schematic of possible results for the RNA-seq data analysis. Correlation plot where the x axis represents the fold change of DEGs in diapause vs. development control (wild type and scramble), and the y axis represents the fold change of DEGs in diapause embryos of transcription factor knockout (KO) vs. scramble. No correlation would indicate that the TF knockout has no effect on the diapause program (upper right). A positive correlation would indicate that the diapause program is enhanced upon TF knockout (middle right). A negative correlation would indicate that the diapause program is decreased by TF knockout and switches to a development-like state (bottom right). (E) Correlation plot between the fold change of DEGs in diapause vs. development control (wild type and scramble) (x axis) and the fold change of DEGs in TF knockout (KO) vs. scramble (in diapause) (y axis). Similar results were observed with either type of control (wild type or scramble). Dots represent individual DEGs with FDR < 0.1. Spearman’s correlation ρ and *p* values are displayed for each transcription factor knockout plot. (F) GO enrichment analysis of the diapause-specific changes observed for the three TF knockouts. Enriched GO terms from gene set enrichment analysis (GSEA) were compared for diapause embryos in non-injected samples (wild type), scrambled-sgRNA-injected samples (scramble), and knockout samples (KO), respectively. Representative GO functions are listed on the right, and functions related to lipid metabolism are highlighted in boxes (see also Tables S6A–S6C). (G) Paralog specialization in diapause and development upon *REST* knockout. Paralog specialization for diapause and development (light blue/light orange) is reduced in the context of *REST* knockout (dark blue/dark orange) compared with control (median expression in both wild-type and scramble samples). *p* value is from two-way ANOVA. See also Figures S5 and S6.

We asked if TFs identified by our evolutionary genomics analyses causally regulate the diapause transcriptional program. To this end, we developed a CRISPR-Cas9-based platform to knockout these TFs in injected killifish embryos (founder generation, F0) and assess the diapause program using single-embryo RNA-seq (Figure 6A). We focused on TFs whose binding sites are significantly enriched in accessible chromatin in diapause, whose expression is upregulated in diapause, and with clear orthologs in killifish (see STAR Methods and Table S5A). Among these TFs, we chose six candidates in the following categories: (1) TFs with previously unknown roles in diapause but association with longevity (REST),^30–32^ (2) TFs with known roles in diapause in other species (and roles in longevity and lipid metabolism) (FOXO3a, FOXO3b),^33–38^ and (3) TFs with roles in lipid metabolism (PPARAa, PPARAb, and PPARG)^39,40^ (Figure 6A).

For each candidate TF, we co-injected 3 single-guide RNAs (sgRNAs) spanning the first two exons of each gene in single-cell embryos and let them develop to the diapause state (Figure 6A; Table S5B). As negative controls, we used non-injected embryos (wild type) and embryos injected with scrambled sgRNAs (scramble) (Figure 6A; Table S5B). This CRISPR-Cas9-based platform allowed ~75% knockout efficiency in injected (F0) killifish embryos (Figures S6E and S6F; Table S5C). Five out of the six TF knockouts led to viable embryos (*PPARAa* knockout was early embryonic lethal) (Table S5D).

We used single-embryo RNA-seq to systematically assess the diapause and development program upon each TF knockout (Figures 6A and 6B; Table S5E). Interestingly, *REST*, *FOXO3a*, and *FOXO3b* knockouts resulted in many transcriptional changes in diapause and little expression changes in development (at least at this stage) (Figure 6C), suggesting that these TFs preferentially impact diapause. By contrast, *PPARAb* knockout led to a stronger effect on gene expression during development than in diapause, and *PPARG* knockout had no effect on either state (Figure 6C). Interestingly, *REST*, *FOXO3a*, and *FOXO3b* knockouts resulted in a shift in the diapause gene expression program away from the diapause state and toward a more “development-like” state (Figures 6D and 6E). Indeed, genes that were downregulated in diapause were upregulated in these knockouts (and vice versa) (Figures 6D and 6E). However, none of these knockouts was sufficient, on its own, to reverse the fate of embryos from diapause to development (Table S5D), possibly due to the complexity of the diapause program.

Functional enrichment analysis of the genes differentially regulated in the *REST*, *FOXO3a*, and *FOXO3b* knockouts highlighted many important functions, notably lipid metabolism (Figure 6F). For example, *REST* knockout led to the upregulation of genes enriched in synaptic transmission and nervous system function, consistent with its role in other species.^41,42^ *REST* knockout also resulted in the modulation of genes enriched in different aspects of lipid metabolism (Figure 6F; Table S6A). *FOXO3a* and *FOXO3b* knockouts led to upregulation of genes involved in cell cycle regulation and stem cell differentiation, which are associated with the developmental state (Figure 6F; Table S6B). *FOXO3b* knockout also resulted in changes in genes implicated in lipid metabolism (Figure 6F; Table S6C). Moreover, *REST* knockout, but not *FOXO3a* or *FOXO3b* knockouts, reduced expression specialization of paralogs (Figures 6G and S6G). Thus, three TFs—REST, FOXO3a, and FOXO3b—are functionally important for the diapause expression program and regulate genes involved in functions that are signatures of diapause such as lipid metabolism. Interestingly, REST had not previously been involved in diapause. In addition, although FOXO TFs were known to impact diapause in invertebrates,^36–38^ they had not been previously implicated in diapause in vertebrates.

This functional analysis identifies key TFs involved in the regulation of the diapause program.

### Lipidomics reveal specific lipids in the diapause state

Several lines of evidence point to lipid metabolism as a central feature of diapause. Gene expression and chromatin accessibility analysis at diapause-specific paralogs showed enrichment of functions related to lipid metabolism (e.g., lipid storage, very-long-chain fatty acid metabolism, and regulation of fatty acid beta oxidation) (Tables S4A and S7A). Moreover, upstream regulators of the diapause gene expression program showed enrichment of for lipid metabolism regulators (e.g., FOXO1 and FOXO3^43^) (Table S7B). Consistently, many of the genes impacted by *FOXO3* and *REST* knockout in diapause are involved in lipid metabolism (Tables S6A–S6C).

**Figure 7.**
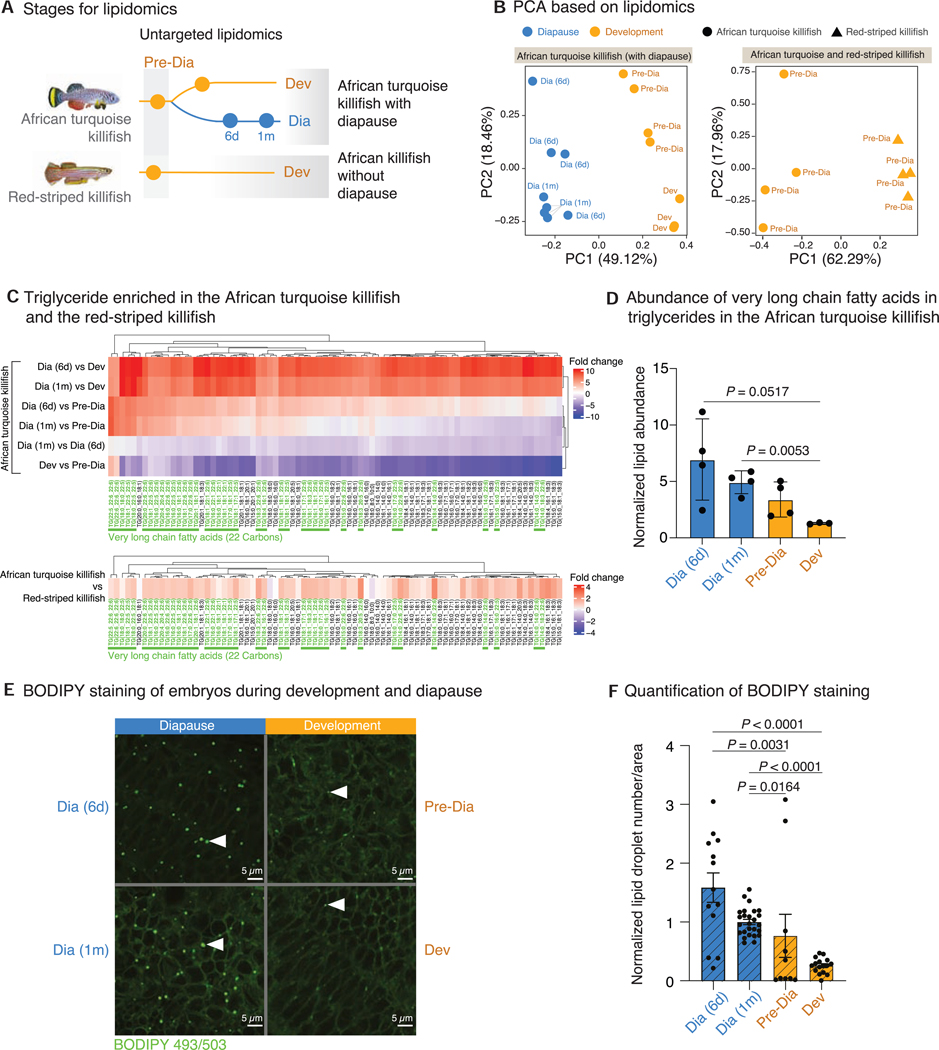
Functional enrichment and lipidomics reveal specific lipids in the diapause state (A) Experimental design for untargeted lipidomics in the African turquoise killifish (with diapause) and the red-striped killifish (without diapause). (B) Principal-component analysis (PCA) on the estimated concentrations for all detected lipids for the African turquoise killifish only (left) and for both killifishes (right). Each point represents an individual replicate from a given species at a given developmental or diapause stage. Variance explained by each principal component (PC) is shown in parentheses. (C) Heatmaps representing the fold change of significantly different triglycerides between diapause vs. development in the African turquoise killifish (upper) and between the African turquoise killifish vs. red-striped killifish (development only, lower). Fold change values are plotted between each pairwise comparison between diapause and development time points, or the two development or diapause time points. For most triglycerides, levels are higher at both 1-month and 6-day diapause relative to development. The bottom panel shows the fold change values of the same lipids in the African turquoise killifish compared with the red-striped killifish; levels of most triglycerides are higher in the African turquoise killifish. Very-long-chain fatty acids among displayed triglycerides species are highlighted in green. (D) Lipid abundance counts for very-long-chain triglycerides (TGs) in the African turquoise killifish during development (orange) and diapause (blue) time points. Bars represent mean ± SEM. Dots represent individual replicates. *p* values from Welch’s t test. (E) Representative images of BODIPY 493/503 staining in the African turquoise killifish embryos in diapause and development (63× objective). Diapause embryos (left) were either 6 days or 1 month in diapause (Dia). Development embryos (right) were either pre-diapause (pre-Dia) or in 1 day of development (Dev). Scale bars, 5 μm. White arrows highlight lipid droplets stained by BODIPY. (F) Quantification of lipid droplet number (normalized to the 1-month diapause time point) in African turquoise killifish embryos stained with BODIPY 493/503. Bars represent mean ± SEM. Each dot represents an individual embryo. Graph represents five experiments merged. *p* values from Welch’s t test (see Table S7D). See also Figure S7.

We therefore asked if lipid profiles differ in diapause. Although lipids and metabolites have been examined in killifish embryos and adults,^44–46^ systematic profiling of lipids in diapause vs. development in different killifish species has not been done. We performed untargeted lipidomics on African turquoise killifish embryos at different times: pre-diapause and diapause at different times (6 days and 1 month). As a comparison, we also performed lipidomics on embryos of another killifish species that does not undergo diapause (red-striped killifish) at a similar state of development just before the onset of diapause in the African turquoise killifish (Figure 7A). The lipidome separated diapause from development in the African turquoise killifish and development in the red-striped killifish by PCA (Figure 7B). Glycerophospholipids (e.g., phosphatidylcholines [PCs] and phosphatidylethanolamines [PEs]), which are membrane lipids, and triglycerides (TGs), which are storage lipids, were changed the most in diapause in comparison with development (Figure S7A; Table S7C). TG changes in diapause are consistent with expression differences of TG metabolism enzymes and regulators, including paralogs (Figure S7D).

Interestingly, we observed an enrichment of TGs containing very-long-chain fatty acids (fatty acids with a chain length of 22 carbons or more) in diapause compared with development, the majority of which are with 5 (docosapentaenoic acid [DPA]) and 6 (docosahexaenoic acid [DHA]) double bonds (Figures 7C, 7D, and S7B). The same TGs with very-long-chain fatty acids were also more abundant in African turquoise killifish embryos at pre-diapause than in red-striped killifish at the equivalent state of development (Figures S7C and S7E). TGs with very-long-chain fatty acids are processed by peroxisomes and subsequently by mitochondria to produce energy,^47^ and they may serve as a long-term energy reserve for diapause.

As many TGs can be incorporated into lipid droplets,^48^ we quantified lipid droplet number in embryos during diapause and development in the African turquoise killifish. We used BODIPY, a dye that stains for neutral lipids and marks lipid droplets.^49^ Lipid droplet number increased in diapause embryos compared with developing embryos (Figures 7E, 7F, and S7F; Table S7D). The lipid droplet number pattern mirrored that of very-long-chain fatty acid abundance in TGs (Figure 7D). Accordingly, expression of several genes involved in TG metabolism and lipid droplets (e.g., *LPIN1*) was also upregulated in diapause, with paralogs showing specialization in diapause (Figure S7D). Thus, TGs with very-long-chain fatty acids and lipid droplet number increase in diapause, which may be critical for long-term energy reserve and usage.

Finally, other lipids, such as many ether-linked glycerophospholipids (plasmalogens), which can protect brain and hearts from oxidative stress,^50–52^ are also more abundant in diapause than development (Figure S7E; Table S7C). Collectively, these data suggest that the African turquoise killifish has evolved to pack specific lipids, including very-long-chain fatty acids and membrane lipids with antioxidant properties, in their embryos. The rewiring of key TF binding sites (e.g., FOXO3 and REST) could modulate lipid metabolism for long-term protection and efficient storage and usage of specific fatty acids.

## DISCUSSION

Our study shows that although diapause evolved recently (less than 18 mya), the paralogs that specialized for diapause are ancestral and shared by most vertebrates (>473 mya). This paralog specialization in the African turquoise killifish diapause is likely achieved by recent co-opting of conserved TFs (such as REST, FOXOs, and PPAR) and repurposing of their regulatory landscape by mutations and selection and transposon element insertion. Building on previous studies,^53,54^ we developed a scalable CRISPR-Cas9-based platform to test the functional impact of TF knockouts on the diapause expression program. This platform reveals that TFs REST and FOXO3 are critical for the diapause transcriptional program and modulate genes involved in functions including lipid metabolism. Indeed, lipid metabolism is distinct in diapause, with accumulation of TGs with very-long-chain fatty acids. Although FOXO TFs were previously known to affect diapause in invertebrates,^36–38^ they were not known to play a role in vertebrate diapause. Interestingly, REST had not been previously implicated in the diapause state. These TFs are likely part of a complex network that controls the diapause program.

Our multi-omics analysis of the diapause state (transcriptomics, chromatin states, and lipidomics) and comparative analysis with several fish species suggest a model for diapause evolution via specialization of very ancient paralogs. After duplication in the ancestor of most vertebrates, these very ancient paralogs likely specialized in the transient response to harsh environment (e.g., transient lack of food, temperature, or other changes), notably by changing lipid metabolism, which ensures their long-term maintenance in the genome. When the ancestors of African turquoise killifish transitioned to ephemeral ponds 18 mya,^14^ these paralogs evolved new TF binding sites, driving further specialization for survival under extreme conditions in diapause. This specialization is most pronounced in very ancient paralogs, with each paralog likely retaining its ancestral molecular function but rapidly acquiring a new TF regulatory network.^55,56^

Elucidating the mechanisms underlying the origin of complex adaptations and phenotypes (e.g., “suspended animation,” novel cell types/tissues, etc.) is a central challenge of evolutionary biology.^57^ Gene duplication is the primary mechanism to generate new genes, and these act as substrate to evolve new functions. Ancient gene duplicates (paralogs) are specialized for expression in different tissues.^16,58^ They can also contribute to the evolution of new organs such as electric organ^59^ and placenta.^60^ Gene duplicates have also been correlated with longevity and exceptional resistance to cancer in long-lived species.^61–63^ Overall, the mechanisms of how divergence of duplicated genes or paralogs contributes to the evolution of complex adaptations are still poorly understood. Emerging evidence, including our study, suggests that complex adaptations can arise by rewiring gene expression by unique regulatory elements.^25,26,64,65^ Our results indicate that this rewiring can be achieved using *de novo* regulatory element evolution and, in some cases, transposon insertion.

*Cis*-regulatory elements such as enhancers and promoters are known to evolve rapidly, and they can in turn facilitate complex adaptations with the same set of conserved genes.^56,66–68^ Transposon insertion can be even faster in promoting the rearrangement of regulatory regions.^55,69^ Rapid reshuffling of regulatory regions by mutation or transposon insertion provides a framework for the evolution of complex trait in nature. Such a mechanism could extend to the mechanism of evolution of other complex traits, including regenerative capacity, which involves new enhancers in killifish.^70^

Our work points to lipid metabolism as being central to diapause. We identify specific lipid species, such as very-long-chain fatty acids, that could be critical for long-term survival in suspended development. Lipids that accumulate in a state of suspended animation (e.g., very-long-chain fatty acids) could serve as key substrates for long-lasting survival.^71,72^ Alternatively, they could signal specific aspects of the diapause state (in addition to known signals in other species, such as vitamin D or dafachronic acid in the South American killifish^73^ and in *C. elegans*, respectively).^74,75^ The pathways and regulatory mechanisms we identified are relevant for other states of suspended animation and even to adult longevity. For example, TFs whose sites are enriched in the diapause state (e.g., FOXOs and PPARs) are genetically required for suspended animation states, such as *C. elegans* dauer,^76–78^ and are expressed in mammalian hibernation.^79^ Furthermore, lipid and lipid metabolism genes are important for mammalian diapause^80,81^ as well as hibernation and torpor,^72,82,83^ and are under positive selection in exceptionally long-lived mammals.^84^ Several of the TFs we identified (e.g., FOXOs and REST) are genetically implicated in longevity and age-related diseases such as Alzheimer’s disease.^30,32,85–87^ Interestingly, TGs have been shown to increase in suspended animation states in other species,^80,88–95^ and lipid droplets are used during diapause and hibernation.^80,81,88^ It will be interesting to understand the functional importance of very-long-chain fatty acids in diapause and other states of longevity.

Our results reveal how selective pressure can lead to the co-optation of key metabolic programs to achieve extreme phenotypes. These observations also raise the possibility that a core program of lipid metabolism genes, regulated by specific TFs, can be deployed to achieve metabolic remodeling and stress resistance in diverse contexts, including in adults. Our study provides a new multi-omic resource for understanding the regulation and evolution of suspended animation states. It also opens the possibility for strategies, including lipid-based interventions, to promote long-term tissue preservation and counter age-related diseases.

### Limitations of the study

To facilitate the interrogation of diapause, we have performed bulk multi-omics at specific time points for diapause and the equivalent stages of development. Although this approach allows for global comparison between the two states, it represents only a snapshot and does not capture changes in cell composition in complex diapause embryos.

Individual knockouts of candidate TFs were not sufficient, on their own, to switch the diapause phenotype toward development. This lack of a strong phenotype could be due to the fact that transcriptomics analysis is performed directly on injected (F0) embryos, which show ~75% knockout. The absence of a strong phenotype could also be due to complexity of the extreme state of diapause or the redundancy of the diapause program. Although we did not observe a direct compensation of TF knockouts by increased expression of another candidate TFs, we cannot rule out compensation via other mechanisms, such as gene networks. Our platform could be expanded to test the combined impact of other TFs (or other genes) on diapause.

Although we have examined the functional role of specific TFs in the diapause gene expression program, it remains important to test their impact on other functions, notably specific lipid accumulation. In future studies, it will also be critical to identify and perturb selected enhancers and decode their regulatory impact on diapause.

## STAR★METHODS

### RESOURCE AVAILABILITY

#### Lead contact

- Further information and requests for resources and reagents should be directed to and will be fulfilled by the lead contact, Anne Brunet (abrunet1@stanford.edu).

#### Materials availability

- This study did not generate new unique reagents.

#### Data and code availability

- All the RNA-seq and ATAC-seq data generated in this study have been deposited to NCBI-GEO (accession # GSE185817) and are publicly available as of the date of publication. Accession numbers are listed in the key resources table. All the lipidomics data generated in this study have been deposited to the Metabolomic Workbench (Study ID ST001898) and are publicly available as of the date of publication. Accession numbers are listed in the key resources table. This paper analyzes existing, publicly available data. These accession numbers for the datasets are listed in the key resources table.
- All original code has been deposited at Github and is publicly available as of the date of publication. DOIs are listed in the key resources table.
- Any additional information required to reanalyze the data reported in this paper is available from the lead contact upon request.

### EXPERIMENTAL MODEL AND STUDY PARTICIPANT DETAILS

#### Killifish species and husbandry

The killifish and other outgroup species used in this study are listed in Table S1A. All the killifish species used for data generation were housed in the Stanford Research Animal Facility II under the approved protocol (protocol #APLAC-13645). Animals were housed in automated circulating water system with pH maintained at 6–7.5 and conductivity maintained between 3500 and 4500μS/cm with a 10% system water exchange every day by reverse osmosis treated water. Adult fish were manually fed Otohime fish diet (Reed Mariculture, Otohime C1 [Ep1 for the South American killifish]) twice a day during weekdays and once a day during weekends.

Newly hatched fries for all species were kept in 0.8-liter fry tanks at a density of 4–5 fries for first two weeks and then individually housed for next two weeks. Fries were fed newly hatched brine shrimps (Brine Shrimp Direct, 454GR) twice a day during weekdays, and once a day during weekends. Animals were sexed at 4 weeks of age and transferred to 2.8-liter tanks. For African turquoise killifish and South American killifish (with diapause), adult males and females were individually housed except for breeding. Red-striped killifish, and lyretail killifish adults were kept in pairs with one male and one female animal in each tank.

For breeding, African turquoise killifish and South American killifish (with diapause) males and females were transferred to breeding tanks for a period of ~5 hours. Breeding tanks had sand trays at the bottom for the African turquoise killifish and trays with extra coarse grade glass beads (30/40 Mesh, 425–560micron size, Kramer Industries Inc. USA) for the South American killifish as per the established protocols.^128–131^ After ~5 hours, sand or glass beads were filtered using a sieve to collect embryos. For the red-striped killifish and the lyretail killifish (without diapause), spawning mops constructed using green yarn were floated from the lid. The yarns were checked every day for embryos, and the embryos were carefully hand-picked.

We used young animals (1–3 months of age) for breeding and embryo collection. For each species, collected embryos were washed multiple times and live embryos were placed in Ringer’s solution (Sigma-Aldrich, 96724) with 0.01% methylene blue at 26°C. Embryos were checked under a stereoscope every day and any dead embryos were removed.

#### Staging of killifish embryos

Synchronized killifish embryos for African turquoise and South American killifish were collected within a tight (~5 hour) breeding window. Most collected embryos were at the 1–2 cell stage upon collection. We monitored embryos every day post-collection to observe the visual markers of diapause and development as previously described.^6^ Briefly, we used Kupffer’s vesicle (KV), which is a transient embryonic organ present from early to middle somitogenesis as a marker to stage embryos that are about to reach diapause. KV-positive embryos reach the end of somitogenesis in 1–2 days and the loss of KV roughly coincides with the onset of heartbeat in killifish, followed by either diapause or continue development.^6,132^ We counted the number of somites in KV-positive embryos and designated KV-positive embryos at 15–25 somites as our *“pre-diapause (Pre-Dia) stage”.* Embryo morphology for all the killifish species was similar at this stage. This mid-somitogenesis time point also coincides with the vertebrate phylotypic period (the period of the most conserved gene expression pattern during vertebrate development) with available gene expression and chromatin accessibility data from multiple other fish species.^24^

In killifish species with diapause, young mothers have most of their embryos develop directly, whereas more mature mothers (even before middle age) have an increased frequency of embryos in diapause.^6,133^ This feature allows us to collect *pre-diapause* embryos, even though there are no known markers, as of yet, to determine if embryos at an earlier stage are destined to diapause. Therefore, for the African turquoise and South American killifish, we collected *pre-diapause (pre-Dia)* embryos from the very first breeding session (first clutch) from young mothers and fathers (age 4–5 weeks) with most embryos expected to skip diapause and continue developing which ensured that we get development bound embryos at *pre-diapause (pre-Dia)* stage.

Among the first visual markers of diapause is the slowing of the rate of heartbeat after its onset.^6,134^ Therefore, we next monitored the onset of heartbeat, and stage diapause embryos at *6 days (Dia 6d)* and *1 month of diapause* (*Dia 1m*) as exhibiting a continuously decreasing heartbeat rate since diapause onset (<45 beat-per-minute (BPM)) as described in Hu et al.^6^ For embryos in 1 month diapause (*Dia 1m*), we additionally made sure that the heartbeat was less than 1 beat per minute by monitoring them under a stereoscope to verify that they were not prematurely exiting the diapause state. For embryos in development, embryos that had an increase in heartbeat rate 1 day after heartbeat onset (>45 BPM), but before the visual pigmentation in eyes was developed (i.e. before pharyngula stage) were designated as *developing embryos (Dev)*.^6^ All the diapause and development stages stage are identical to our previous study,^6^ except *Pre-Dia* stage which is roughly a day before the onset of heartbeat. For killifish species without diapause (red-striped and lyretail killifish), we followed the same staging procedure described above to collect embryos at *pre-diapause (pre-Dia) stage* (development embryos with 15–25 somites; ~1 day before the onset of heartbeat). Because there is no diapause in these killifish, development embryos were taken as 1 day after the onset of heartbeat to match to the *Dev* stage in the African turquoise killifish.

#### Killifish embryo collection

For each stage in each species, roughly 8–30 embryos were carefully dissected in ice-cold PBS using biological-grade tweezers (Electron Microscopy Sciences, 72700-D) to carefully remove the chorion, the enveloping layer, and the yolk without damaging the embryo body. Freshly dissected embryos were then quickly rinsed with ice-cold PBS, and all the PBS was carefully removed. Embryo bodies were then snap-frozen in liquid nitrogen and stored at −80°C. We used 8–10 snap-frozen embryos for RNA-seq and ATAC-seq and 25–30 embryos for lipidomics (see below). The details of all samples and stages used are in Table S1B.

### METHOD DETAILS

#### RNA-seq preparation for killifish species

To profile gene expression at pre-diapause stages in the African turquoise, red-striped and lyretail killifish, we constructed RNA-seq libraries (Table S1B, GSE185815, https://www.ncbi.nlm.nih.gov/geo/query/acc.cgi?acc=GSE185815). Snap frozen embryos at −80°C were thawed on ice for 1 minute and washed with 200μl ice-cold PBS. The embryos were then dissociated and homogenized with ~25 Zirconia/Silicon 0.5mm glass beads (RPI, Research Products International Corp, 9834) using FastPrep^®^ −24 homogenizer (MB Biomedicals, 116004500) for 20 seconds, followed by centrifugation (17000g for 3 minutes). After centrifugation, 10.5ml of the supernatant was used as input to the SMART-Seq^®^ v4 Ultra^®^ Low Input RNA Kit (Takara, 634890) for the cDNA synthesis followed by amplification with 12 cDNA amplification cycles. Amplified cDNA was validated with Agilent 2100 Bioanalyzer using Agilent’s High Sensitivity DNA Kit (Agilent, Cat. No. 5067–4626). The DNA libraries were then generated using the Nextera XT DNA Library Prep Kit (Illumina, FC-131–1096). Library quality and concentration were assessed by the Agilent 2100 Bioanalyzer and Agilent’s High Sensitivity DNA kit (Agilent Technologies, Cat. No. 5067–4626), followed by high throughput sequencing on Illumina HiSeq platform with 2 × 150bp paired end reads.

In addition, we also used available African turquoise killifish,^6^ South American killifish,^9,73^ medaka,^24^ zebrafish^24,96^ and mouse^22^ embryo RNA-seq data for our analysis (Table S1B), and processed them using the same pipeline described below. For medaka and zebrafish, we used mid-somitogenesis stages for our analysis that are expected to be the closest across vertebrates^24^ (Table S1B).

#### ATAC-seq library preparation

To identify diapause-specific regulatory regions in the genome of African turquoise killifish and how these have evolved, we performed the Assay of Transposase Accessible Chromatin followed by high throughput sequencing (ATAC-seq)^23,135^ in the embryos of multiple species. ATAC-seq is an unbiased and sensitive assay of genome-wide accessible chromatin landscape that requires very low input material. We performed ATAC-seq on embryos collected from five different killifish species with and without diapause, and at different stages of development and diapause (Table S1B). To generate nuclei-suspension for ATAC-seq libraries, snap frozen embryo samples (~10 embryos per sample) were thawed for 1 minute and resuspended at 4°C in 200μl EZ-lysis buffer (Sigma Aldritch No. 3408). Samples were then transferred to 250μl mini-douncers (DWK (Kimble) 885300–0000) and dounced 25 times with pestle A and B respectively. After a 2 minute incubation following douncing, samples were spun at 500g for 5 minutes to precipitate nuclei, and the EZ-lysis supernatant was removed. Nuclei were then resuspended in 250μl PBS (ThermoFisher No. AM9624) and an aliquot of 5μl of nuclei was incubated with 5μl of 0.4% trypan blue stain (ThermoFisher No. 15250061) for counting the total intact nuclei counts.

Samples of ~25,000 nuclei were then suspended in a Tn5 transposition mix (65μl of tagmentation DNA buffer (Illumina No. 20034197), 63μl of nuclease-free water, and 2.5μl of tagmentation DNA enzyme I (e.g Tn5 transposase) (Illumina No. 20034197) for 20 minutes at 37°C. Following incubation, the mix was purified using the Qiagen mini-elute kit (Qiagen No. 28206) to isolate tagmented DNA. PCR amplification and subsequent qPCR monitoring was performed as described in the original ATAC-seq protocol (~14–18 cycles of PCR ).^23^ Amplified DNA from the PCR reaction was purified using the Qiagen mini-elute kit (Qiagen No. 28206), as recommended by the manufacturer. Samples were subsequently pooled and sequenced using next-generation short-read sequencing on an Illumina Nextseq 550 (Illumina No. PE-410–1001) with 75bp paired-end reads.

#### Generation of F0 knockout embryos

African turquoise killifish embryos were collected from breeding tanks, each with 1 young GRZ male (1.5–3 months) and 3 young GRZ females (1.5–3 months), co-housed for only 3–4 hours to ensure the collection of embryos at the single-cell stage. The relatively young age for females was chosen so that there would be ~50%−85% of embryos destined for diapause and ~15%−50% of embryos destined for direct development.^6^ Once collected, viable embryos were washed with ~1ml of embryo solution (Ringer’s solution with 0.01% methylene blue). Once cleaned, embryos were mounted in a 2% agarose gel mount with ~1mm width and ~1mm deep grooves to hold embryos in place for injection.^136^ While in the gel mount, embryos were split into 3 groups per embryo collection: i) Wildtype (non-injected embryos); ii) Scramble (embryos injected with scrambled sgRNAs and Cas9). For these embryos, ~0.01μl Alt-R™ S.p. Cas9 Nuclease (22μM, IDT, Cat. No. 1081058) and three scrambled sgRNAs (~7.3μM each, 22μM total, IDT) were injected (Table S5B); or iii) F0 knockout embryos (injected with target transcription factor sgRNAs and Cas9). For these F0 knockout embryos, ~0.01μl of Cas9 (22μM) and three sgRNAs (~7.3μM each, 22μM total) targeting the first two exons of the candidate of interest (*REST*, *FOXO3a*, *FOXO3b*, *PPARAa*, *PPARAb*, or *PPARG*) were injected. For prioritization of candidates, see sections below: “TF knockout selection” and “sgRNA design”. Embryos from each embryo collection were divided roughly equally among these 3 groups. Injected embryos from these experiments (F0 generation) were monitored for the following 10–14 days until the desired stage of diapause/development was reached. During this period, both embryo survival and diapause entry rates were tracked (Table S5D). Embryo death was monitored on a daily basis. Entry in diapause was assessed by using the method previously described in,^6,132^ which involves heartbeat onset. Heartbeat onset and rate was assessed daily on a dissection scope manually. It represents a very robust method to distinguish embryos in diapause and embryos in development.^6,132^ One day after heartbeat onset was used to determine entry into diapause (heartbeat < ~20 beats per minute) or direct development (heartbeat > ~85 beats per minute). Embryos in development were processed on 1 day post heart beat onset. Embryos in diapause were incubated for an additional five days to reach the ‘6-days in diapause’ timepoint.

#### Knockout RNA-seq library generation

To generate single embryo RNA-seq libraries, individual injected (F0) embryos were dissected at the desired stages: development (1 day post-heartbeat onset, high heartbeat) and diapause (6-days post-heartbeat onset, low heartbeat). For dissection, the dissection scope and tools were treated with RNase Zap^™^ to prevent contamination during sample preparation. Dissection was carried out in 1x PBS chilled to 4°C, as previously described.^137^ Briefly, forceps were used to remove both the chorion and embryonic membrane, removing the yolk, and taking only the embryo body for collection into one 1.5ml Eppendorff^™^ DNA loBind microcentrifuge tube. Each embryo was placed in one single tube and excess dissection supernatant (4°C 1x PBS) was removed. Each injected (F0) embryo represents an independent sample and it is entirely used for RNA-seq and knockout validation. The dry single embryos were then centrifuged (14,000g for 1 minute at 4°C) and resuspended in 15ml of 4°C 1x PBS and were subsequently dissociated and homogenized with ~25 Zirconia/Silicon 0.5mm glass beads (RPI, Research Products International Corp, 9834) using FastPrep^®^ −24 homogenizer (MB Biomedicals, 116004500) for 1 minute, followed by centrifugation (10,000g for 1 minute at 4°C). After centrifugation, 10μl of the supernatant was used as input to the SMART-Seq^®^ v4 Ultra^®^ Low Input RNA kit (Takara, 634890) for cDNA synthesis followed by amplification with 12 cDNA amplification (PCR) cycles (1min at 95°C, 12 cycles of [10sec at 98°C, 30sec at 65°C, 3min at 68°C], 10min at 72°C, and held at 4°C). Amplified cDNA was validated with Agilent 2100 Bioanalyzer using Agilent’s High Sensitivity DNA Kit (Agilent, Cat. No. 5067–4626). The DNA libraries were then generated using 1ng of cDNA material from each prep, using the Nextera XT DNA Library Prep kit (Illumina, FC-131–1096), following the manufacturer’s instructions. Library quality and concentration were assessed by the Agilent 2100 Bioanalyzer and Agilent’s High Sensitivity DNA kit (Agilent Technologies, Cat. No. 5067–4626), followed by high throughput sequencing on Illumina Nova-Seq 6000 platform with 2 × 150bp paired-end reads.

#### CRISPR/Cas9 knockout validation

We used several independent methods to validate editing events using our single embryo lysates. After the glass bead homogenizing and centrifugation steps described above, we also used ~0.5–5μl of supernatant for genotyping as the embryo lysates should also contain genomic DNA. This supernatant was added to 20μl of PCR mix (10μl 2X DreamTaq PCR master mix, 8μl of water, and 1μl of custom forward and reverse primers for each gene of interest; Table S5B) and amplified for 40 cycles (2min – 95°C, 40 cycles of: 30sec – 95°C, 30sec – 59°C, 1min – 75°C). PCR products were submitted to Molecular Cloning Laboratories (MCLab) for PCR-cleanup (Cat. No. SEQ-CU) and sent for Easy Format^™^ Reactions for Sanger sequencing (Cat. No. SEQ-EZ).

Sanger sequencing chromatograms were visualized using SnapGene v7.0 to assess if they had biphasic peaks at sgRNA sites – a characteristic of the presence of different bases at one location. We also aligned each chromatogram to the reference gene in the African turquoise killifish genome to inspect the nature of the mutations (Nfu_20140520). Finally, the sequences were assessed for potential knockouts using the Synthego ICE analysis platform v1.0. The wildtype or control sgRNA-injected sample sequences with the highest quality score and best alignment to the locus of interest were used as the background, ensuring high quality ICE-scores for all sequences. The Synthego ICE platformed aligned all 122 samples’ Sanger-sequencing products (56 Wildtype/Scramble and 66 knockouts). From these samples, the mutant libraries had a median predicted knockout score, based on ICE alignment of chromatograms, of 75%, ranging from 45% (*PPARAb* knockout) to 94% (*FOXO3b* knockout) (Figures S6E and S6F; Table S5B). We used the combination of these metrics and validation of the RNA-seq reads to inform our downstream analysis (see Criteria below).

#### Untargeted lipidomics by LC-MS

Lipidomics experiments were performed using ~30 embryos for each stage of diapause and development from African turquoise and red-striped killifish (3–4 replicates for each stage) (Figure 7A) as previously described.^138,139^ We specifically chose the pre-diapause timepoint (day of heartbeat onset) as it is a well-conserved window of development across many species. This allows for comparison between species at this timepoint.

Lipids were extracted in a randomized order via biphasic separation with cold methyl tert-butyl ether (MTBE), methanol and water. Briefly, 260μl of methanol and 40μl of water were added to the embryos and vortexed for 20 seconds. A lipid internal standard mixture was spiked in each sample (EquiSPLASH LIPIDOMIX, Avanti Polar Lipids (cat #: 330731), and d17-Oleic acid, Cayman chemicals (cat #: 9000432) to control for extraction efficiency, evaluate LC-MS performance and estimate concentrations of individual lipids. Samples were diluted with 1,000μl of MTBE, vortexed for 10 seconds, sonicated for 30 seconds three times in a water bath, and incubated under agitation for 30 minutes at 4°C. After addition of 250μl of water, the samples were vortexed for 1 minute and centrifuged at 14,000g for 5 minutes at 20°C. The upper phase containing the lipids was collected and dried down under nitrogen. The dry extracts were reconstituted with 150μl of 9:1 methanol:toluene.

Lipid extracts were analyzed in a randomized order using an Ultimate 3000 RSLC system coupled with a Q Exactive mass spectrometer (Thermo Fisher Scientific) as previously described.^139^ Each sample was run twice in positive and negative ionization modes and lipids were separated using an Accucore C30 column 2.1×150mm, 2.6μm (Thermo Fisher Scientific) and mobile phase solvents consisted in 10mM ammonium acetate and 0.1% formic acid in 60/40 acetonitrile/water (A) and 10mM ammonium acetate and 0.1% formic acid in 90/10 isopropanol/acetonitrile (B). The gradient profile used was 30% B for 3min, 30–43% B over 5min, 43–50% B over 1min, 55–90% B over 9min, 90–99% B over 9min and 99% B for 5min. Lipids were eluted from the column at 0.2ml/min, the oven temperature was set at 30°C, and the injection volume was 5μl. Autosampler temperature was set at 15°C to prevent lipid aggregation.

LC-MS peak extraction, alignment, quantification, and annotation was performed using LipidSearch software version 4.2.21 (Thermo Fisher Scientific). Lipids were identified by matching the precursor ion mass to a database and the experimental MS/MS spectra to a spectral library containing theoretical fragmentation spectra. The following lipid ions were used for quantification: [M+H]+ for ceramides (Cer), (lysophosphatidylcholine) LPC, phosphatidylcholine (PC), monoglycerides (MG) and sphingomyelins (SM); [M-H]- for phosphatidylethanolamines (PE), phosphatidylinositols (PI), phosphatidylserines (PS), phosphatidylgylcerols (PG) and lysophosphatidylethanolamine (LPE); and [M+NH4]+ for cholesterol ester (ChE), diglycerides (DG) and triglycerides (TG). To reduce the risk of misidentification, MS/MS spectra from lipids of interest were validated as follows: 1) both positive and negative mode MS/MS spectra match the expected fragments, 2) the main lipid adduct forms detected in positive and negative modes agree with the lipid class identified, 3) the retention time is compatible with the lipid class identified and 4) the peak shape is acceptable. The fragmentation pattern of each lipid class was experimentally validated using lipid internal standards.

Single-point internal standard calibrations were used to estimate absolute concentrations for 431 unique lipids belonging to 14 classes using one internal standard for each lipid class. Importantly, we ensured linearity within the range of detected endogenous lipids using serial dilutions of internal standards spanning 4 orders of magnitude. Median normalization (excluding TG and DG) was employed on lipid molar concentrations to correct for differential quantity of starting material. Importantly, we verified that median lipid signal (excluding TG and DG) correlated well (Pearson’s correlation coefficient = 0.48, P = 0.005) with the total protein content in each sample as measured by the BCA Protein Assay Kit (Pierce, cat# 23225) from precipitated proteins following the biphasic separation, suggesting good sample quality. One development (diapause escape) sample had an unexpectedly low protein concentration and thus was discarded. Lipid molar concentrations for a given class were calculated by summing individual lipid species molar concentrations belonging to that class. Fatty acid composition analysis was performed in each lipid class. Fatty acid composition was calculated by taking the ratio of the sum molar concentration of a given fatty acid over the sum molar concentration across fatty acids found in the lipids of the class. Subsequently, saturated fatty acids (SFA), mono-unsaturated fatty acids (MUFA) and poly-unsaturated fatty acids (PUFA) were grouped together for comparative analysis.

#### Lipid droplet imaging

Embryos were imaged with a Zeiss confocal microscope (LSM900, Axio Observer) equipped with the Zen software (3.0, blue). Within each experiment, the same laser power and settings were used across all conditions. For whole embryo imaging a 5x air objective (Fluar 5x/0.25 M27) and a 22 μm pinhole were used to image the 20μm depth (5 slices, 5μm intervals, dorsal-to-ventral stack) of the fish. Z-stack projections were generated in Fiji version 2.0.0.^140^ For zoomed-in visualization of lipid droplets a 63x oil objective (Plan-Apochromat 63x/1.40 Oil DIC M27) and a 32 μm pinhole were used to image the lipid droplets over a range of 0.72 μm (5 slices, 0.18 μm). Z-stack projections were generated in Fiji version 2.0.0 (Figures 7E and S7F).

For lipid droplet quantification, a 20× air objective (Plan-Apochromat 20x/0.8 M27) and a 32 μm pinhole was used to image the embryos in at least 3 different positions along their body over a range of 4 μm (3 slices, 2 μm intervals, dorsal-to-ventral). Lipid droplet number was quantified by generating z-stack projections of 3 slices, subtracting the background, applying the same threshold to all images, and quantifying the lipid droplet number in a 100 × 100 μm^2^ area using the analyze particle function in Fiji version 2.0.0. The lipid droplet number was averaged across all locations imaged for one individual. For each condition, at least 3 embryos were imaged. Experiments were carried out at least two times independently. The lipid droplet number was normalized to the “Diapause 1 month” condition of the respective experiment, all experiments are plotted together in Prism 9 and statistically significant differences between samples were assessed using a Kruskal-Wallis test for differences in mean (Figure 7F). See Table S7D for unprocessed lipid droplet numbers and statistical differences (Mann-Whitney U-test) within one experiment.

### QUANTIFICATION AND STATISTICAL ANALYSIS

#### Identification and dating of paralogs

We focused our analysis on paralogs because i) gene duplication or paralogs are the primary mechanism by which new genes originate and specialize for new functions or states^12,13^; ii) paralogs also allow for a precise timing of the evolutionary origin of specific genes, and iii) the majority of genes in killifish are in paralog pairs owing to multiple rounds of genome duplicates. To generate a comprehensive resource of paralogs in multiple killifish species and to date their duplication time relative to other species, we used the OrthoFinder pipeline.^15,100^ To this end, we collected genome sequences from multiple killifish species with and without diapause from published reports and NCBI genome,^8,9,29^ other teleost fish, mammals, and non-vertebrate outgroups from Ensembl (version 100).^141^ Phylogenetic tree-based inference of orthologs, paralogs, and relative duplication timing of each paralog in all these species was done by OrthoFinder. OrthoFinder infers “groups” of genes or gene families including both ortholog and paralog for all species used in the analysis (called *orthogroups*). Gene trees were built for all these *orthogroups* and reconciled with the rooted species tree to identify gene duplication events and their relative duplication time based on a phylogenetic approach.^15,100^ Note that for a single species in an *orthogroup*, one gene can be a paralog partner with multiple other genes making groups of paralogs. We filtered out the paralog groups with >20 paralog partners for a gene to exclude large inter-connected paralog groups, which can inflate the overall pairwise analysis. Note also, that our results were not dependent on the paralog group or family size (Figures S1D–S1F; Table S1D). Duplication node and approximate timing of the duplication (in million years ago [mya]) for each paralog pair was annotated based on known phylogenetic tree from Ensembl for species covered in Ensembl version 100^141^ or published reports for killifish species.^14^ To ensure that our results were not affected by the choice of species and outgroups used, we used 3 different sets of species to run the complete OrthoFinder pipeline independently: a set of 71 species, 31 species, and 13 species. The three pipelines resulted in very similar estimates of relative duplication time for killifish paralogs and the results were qualitatively identical (Table S1D). We used paralogs identified by OrthoFinder analysis with 71 species for our study (20,091 paralog pairs in African turquoise killifish, *Nothobranchius furzeri*, 22,955 pairs in the South American killifish, *Austrofundulus limnaeus* genomes), and 13,437 pairs in mouse, *Mus musculus*, genome.

In addition to OrthoFinder, we also annotated the paralog duplication timings in the African turquoise killifish directly from Ensembl version 84 using an independent approach. To identify the paralog pairs in the African turquoise killifish genome, we first identified high confidence one–to-one orthologs (bi-directional best hits) between the African turquoise killifish and each of the 5 teleost fish species (zebrafish, *Danio rerio*; medaka, *Oryzias latipes*; stickleback, *Gasterosteus aculeatus*; tetraodon, *Tetraodon nigroviridis*; and fugu, *Takifugu rubripes*) using BLASTp (E-value 1e-03).^101^ We next identified paralogs in each of the five teleost fish for which both the genes had one-to-one orthologs in African turquoise killifish, and assigned their duplication time to the African turquoise killifish paralog. Because Ensembl did not have any killifish species, the paralogs duplicated in the killifish lineages after the divergence from medaka would be missed. Therefore, to identify such paralog pairs, we performed a protein family clustering using all the protein coding genes for multiple killifish species with and without diapause along with other teleost fish. We then annotated the duplication time for each of the potential paralogs that were not already identified using the ortholog analysis as “teleost” (if they were shared with the other teleost fish), “aplocheiloidei” (i.e. common ancestor of all killifish, if it was shared only by killifish species without diapause), and “nothobranchius” or “*Nothobranchius furzeri*” (shared by nothobranchius genus or only present in the African turquoise killifish, respectively). This independent pipeline also resulted in very similar estimates of relative duplication time for killifish paralogs and the results were qualitatively identical (Table S1D). A total of genes that were not observed in any of our four paralog analysis pipelines (OrthoFinder with 71, 31, or 13 species or with Ensembl) were classified as singleton genes.

To simplify the interpretation and analysis, the relative duplication nodes from each analysis were divided into 3 categories: *very ancient* (paralogs duplicated in the ancestor of jawed vertebrates at nodes Gnathostomata and earlier i.e. >473.3 mya), *ancient* (paralogs shared by most teleost fish species, duplicated between nodes Ovalentaria and Gnathostomata at 111–473.3 mya or earlier), *recent* (paralogs shared by most killifish species, duplicated between nodes Ovalentaria and *Nothobranchius furzeri* at < 111 mya)^141^ (Figures 1D and S1D–S1F). Diapause-specialized paralog numbers (see below) in each of the three categories were compared to the genome average in that category with 10,000 bootstraps resampling of 50% paralogs genome-wide (Figures 1E, 2E, S1D–S1F, and S2H). For mouse diapause, *very ancient* paralogs pairs were defined similar to killifish, *recent* paralogs were shared by all the mammals in our data and *recent* paralogs were shared only by eutherian mammals (Figure S2H).

#### Classifying paralogs specialized for diapause

To identify the African turquoise killifish paralog pairs that show signs of specialization of the gene expression pattern for diapause, we used the normalized RNA-seq expression from Hu et al.^6^ (see below). This dataset consists of two stages during African turquoise killifish development (heartbeat onset and diapause escaped embryos 1-day post heartbeat onset) and three time points during diapause (diapause embryos at 3 days, 6 days, and 1 month in diapause). We first identified differentially expressed genes in all three diapause time points with respect to both development time points using DESeq2 (version 1.30.1).^102^ A paralog gene pair was classified as having specialization of expression if one gene was significantly upregulated in one of the three diapause time points (FDR < 0.05) with respect to one of the two development stages, and the other partner gene was significantly downregulated in diapause or had a median expression in development higher than median expression in diapause. This resulted in 6,247 paralog pairs with expression specialization in diapause with the 71 vertebrate OrthoFinder pipeline (Table S1C). To test robustness, we used several different criteria to identify diapause-specialized paralogs (different FDR cutoffs, and different combinations of differentially expressed genes). To test robustness, we used several different criteria to identify diapause-specialized paralogs (different FDR cut-offs, and different combinations of differentially expressed genes), and our results were robust to the changes in FDR cutoffs.

We independently identified paralogs specialized for South American killifish diapause, using RNA-seq data of South American killifish embryos in diapause and development (4 days post diapause exit) from Wagner et al.^9^ Paralogs with one gene significantly expressed (i.e., upregulated) in diapause compared to development (FDR < 0.05), and the other gene significantly downregulated in diapause compared to development (FDR < 0.05) were classified as specialized paralogs (2,480 pairs). Note that the diapause and post-diapause development stages are not an exact match to the African killifish stages, these stages are within a similar timing window and separate together by PCA (see Figure S2A)

Paralogs specialized for mouse diapause were identified using RNA-seq data of mouse embryos in *diapause* (Pre-implantation, diapause blastocyst) and development (pre-implantation Inner Cell Mass (*ICM*), day 3.5 post-fertilization; and post-implantation epiblasts (*Epi*), day 6.5) from Hussein et al.^22^ The RNA-seq data was reanalyzed using the mouse reference genome^97^ and the same processing pipeline as the African and the South American killifish (see below). Paralogs with one gene significantly upregulated in diapause compared to *ICM* and *Epi* (FDR < 0.05), and the other gene down in diapause compared to both *ICM* and *Epi* were classified as specialized paralogs (201 pairs). The lower numbers of specialized pairs in diapause are likely due to less extreme nature of mouse diapause compared to killifishes.

#### Assessing paralog divergence and location

The set of paralog pairs described above were aligned and the rate of synonymous (dS) and non-synonymous (dN) mutations were evaluated using the PAML package (v4.8). The ratio dN/dS (or omega ratio; ω) was calculated between each pair, assessing their difference in sequence from one another as opposed to their changes from an outgroup species or common ancestor. We then used this list of single ω-ratio per-pair to evaluate any difference in sequence divergence between the genome-wide paralog set and paralog pairs identified with specialized expression for diapause. Diapause specialized paralogs were detected to have significantly less sequence divergence than their genome-wide counterparts (Mann-Whitney U test, *P* = 2.2e-16) (Figure S1J, left). This significant difference was also observed when subsetting the paralog pairs by time of duplication in both the very ancient (*P* = 2.12e-04) and ancient (*P* = 9.779e-09), and trending in the recent/very recent category (*P* = 0.0619) (Figure S1J, center-left to right, respectively).

Additionally, we assessed the chromosomal locational of each of our defined paralog pairs. The pairs were divided into two groups: 1) paralog pairs in which both members are located on the same chromosome and 2) the paralogs are on different chromosomes. When examining the genome-wide distribution, a majority of paralog pairs were found on separate chromosomes in the African turquoise killifish. However, there were significantly less diapause-specialized paralog pairs that are located on the same chromosome than the genome average (Mann-Whitney U-test, *P* =4.71e-12) (Figure S1G). This difference was also observed when partitioning the paralog pairs by age of duplication in both the very ancient (*P* = 2.257e-02) and ancient (*P* = 2.627e-07) (Figure S1H, left and center). However, this difference was not observed in the recent/very recent paralog group, which are roughly equally distributed in tandem and on separate chromosomes (*P* = 0.7498) (Figure S1H, right).

All these paralogs, along with their expression level, genomic locations, and specialization in diapause are included in Table S1C.

#### RNA-seq data processing pipeline

We first trimmed the adaptors from raw sequencing FastQ files using Trim Galore (version 0.4.5) followed by read quality assessment using FastQC^103^

(version 0.11.9) and MultiQC (version 1.8).^104^ Adaptor trimmed files were aligned to the respective genome (Table S1A) using STAR (version 2.7.1a).^105^ No reference genome is available for the red-striped killifish, so the reads from red-striped killifish RNA-seq libraries were aligned to the genome of its close relative, lyretail killifish. Identification of accurate gene expression values for paralogs can be challenging if the reads align to both the genes in the pair equally well. Therefore, we excluded all the reads that mapped to multiple locations in the genome, and only kept reads that align uniquely to a single genomic locus with samtools (version 1.5) using “*samtools view -q255*” command. Read counts were then assessed using featureCounts function in Subread package (version 2.0.1).^106,142^ Raw gene expression values were then normalized using DEseq2 (version 1.30.1).^102^ Because different RNA-seq datasets were generated separately, we performed separate normalization for each of the individual analyses.

#### ATAC-seq data processing pipeline

To process ATAC-seq, we first removed adaptors from FastQ files using TrimGalore (version 0.4.1), followed by read quality assessment with FastQC^103^

(version 0.11.9) and MultiQC (version 1.8).^104^ Reads were then aligned to their respective reference genomes (Table S1A) using BowTie2 (version 2.2.5)^107^ with “*–very-sensitive*” option. No reference genome is available for the red-striped killifish, so the reads from red-striped killifish ATAC-seq libraries were aligned to the genome of the closest sequenced species, lyretail killifish. Duplicates were marked using Picard (version 2.22.1). Duplicates, multimapping reads (MAPQ < 20), unmapped and mate-unmapped reads (only one read of the pair mapped), not primary alignments, and reads failing platform were then removed using SAMtools (version 1.5).^108^ Because the Tn5 transposase binds as a dimer and inserts two adaptors separated by 9bp, all aligned read positions on + strand were shifted by +4bp, and all reads aligning to the – strand were shifted by —5bp, using alignmentSieve in deepTools (version 3.2.1).^23,109^ We called peaks using MACS2 (version 2.1.1.20160309)^110,143^ using different effective genome size for each species (e.g., genome size after removal of gaps represented by Ns).

Library quality was assessed using metrices recommended by ENCODE consortium including fragment length distribution to assess nucleosome banding patterns and enrichment of ATAC-seq peaks at transcription start sites. We observed the nucleosome banding patterns strongly in many of ATAC-seq libraries, though some lacked strong indication of classical band spacing of nucleosomes. We believe this is due to the particularly fragile nuclei/chromatin structure of killifish embryos (requiring orders of magnitude less transposase enzyme to yield efficient cutting. This led to some libraires being ‘over-transposed’. To address if these libraries were still sufficient quality for downstream analysis, we evaluated other metric to assess library quality such as transcription start site read enrichment, PCR bottleneck coefficients (PBC1 and PBC2), and fraction of reads in peaks (FRiP) (Table S3A). There was a significant enrichment of ATAC-seq peaks at transcription start sites as expected (Figures S3F and S3G). Other quality metrices were also above the threshold recommended by the ENCODE consortium (Table S3A).

ATAC-seq data from medaka and zebrafish for corresponding development stages were obtained from Marlétaz et al.^24^ (Table S1B) and processed using the same pipeline described above. We used development stage 19 and 25 in medaka and 8-somites and 48 hours post fertilization in zebrafish, which are expected to correspond to pre-diapause and development in the African turquoise killifish respectively. These were used for chromatin accessibility conservation analysis presented in Figures 3, 4, 5, and S3–S5.

#### Multiple whole-genome alignment

To integrate ATAC-seq and RNA-seq datasets across species, we performed a 5-way multiple whole-genome alignment with African turquoise killifish (*Nfur: Nothobranchius furzeri*), lyretail killifish (*Aaus: Aphyosemion australe*), South American killifish (*Alim: Austrofundulus limnaeus*), medaka (*Olat: Oryzias latipes*) and zebrafish (*Drer: Danio rerio*) (Table S1A), using African turquoise killifish as the reference genome. For red-striped killifish (*Aphyosemion striatum*), genome of the closest sequenced species lyretail killifish was used for integrative analysis. For genomes with chromosome level assemblies, we discarded scaffolds not placed on chromosomes. First, we performed pairwise alignments between African turquoise killifish and each of the four other fish genomes using LASTZ^111^ (parameters: –gap=400,30 –gappedthresh=3000 –ydrop=6400 –inner=2000 –hspthresh=1500 –masking=50 –no-transition –step=20 –scores=HoxD55.q). Subsequent chaining and netting were performed using the suite of UCSC genome browser utilities.^112^ The percentage of aligned African turquoise genome to each of the other fish species decreased based on the distance to the last common ancestor as expected^144^ with 61.1%, 47.8%, 23.2%, 20.1% of the African turquoise killifish genome aligning to the lyretail killifish, South American killifish, medaka and zebrafish genomes respectively in a pair-wise manner.

These pairwise alignments were then merged using the multi-alignment tool Multic/TBA,^113^ using the command <*tba + E=Nfur ((((Nfur Aaus) Alim) Olat) Drer./pairwise_dir/*> to obtain a single, 5-way, multiple whole-genome alignment using the African turquoise killifish genome as the reference (specified by *E=Nfur*). The resulting multiple-whole genome alignment covered ~75.3% of the African turquoise killifish genome. Coverage of each of the aligned fish genome in the multi-alignment also diminished as time to the last common ancestor increased with 62.7%, 85.9%, 14.2%, and 23.7% of the genome being covered for lyretail killifish, South American killifish, medaka, and zebrafish genomes respectively.

To assess the quality of our genome alignment, we compared the length of aligned sequence blocks in multi-genome alignment with that of teleost fish 8-way multi-genome alignments available from the UCSC genome browser and generated using a similar approach^145^ (https://hgdownload.soe.ucsc.edu/goldenPath/danRer7/multiz8way/). We found that the aligned block lengths in both our and 8-way multi-genome alignment from UCSC were comparable. Most of the aligned blocks were either 10–99bp long (53% our vs 38.7% UCSC-fish) or 100–999bp long (33% our vs 26.5% 8-way alignment from UCSC) in both the alignments. Importantly, a vast majority of our ATAC-seq peaks (98.35% of chromosomal peaks) fall in the regions that are covered in our multi-genome alignment.

#### Integrating ATAC-seq across species

The 5-way multiple whole-genome alignment was used to compare ATAC-seq data across species. Bed files for each ATAC-seq library were cross-referenced to the alignment and the coordinates of ATAC-seq peaks for all species were converted to African turquoise killifish genome coordinates. During this process, peaks were tagged as “conserved” at three levels of stringency: relaxed (any base pair overlap between peaks), strict (25% of the African turquoise killifish peak must be covered by aligned peak region in other species), and very strict (50% of the African turquoise killifish peak must be covered by the aligned peak in other species). The differences in peak conservation between relaxed and strict definitions was qualitatively minimal. Thus, subsequent analysis was performed with the relaxed peak set. During coordinated conversion, some peaks for species other than African turquoise killifish became split between two or more locations in the African turquoise killifish genome. We also included these split location peaks in our analyses. However, split location peaks represent only a minority of recovered peaks (5.2%) and are unlikely to influence our analyses.

With this finalized peak set, we then categorized each peak in African turquoise killifish and its underlying sequence into one of three conservation categories: *ancient/very ancient, recent, and very recent*. 1) Peaks considered *very recent* had only a peak in the African turquoise killifish (likely originated after divergence from killifish species without diapause at < 17.79 mya)^14^). 2) Peaks considered *recent* had overlapping peaks in African turquoise killifish and at least one other African killifish (i.e. lyretail killifish or red-striped killifish), but not in outgroups (medaka and zebrafish; likely originated between 17.79–93.2 mya).^14,141^ 3) Peaks considered *ancient/very ancient* had overlapping peaks in African turquoise killifish, at least one other African killifish (i.e. lyretail killifish or red-striped killifish), and at least one outgroup fish (i.e. medaka or zebrafish; likely originated > 93.2 mya)^141^ (Tables S3B and S3C). To avoid confounding peaks within our *very recent* category, peaks present in the African turquoise killifish, absent in other African killifish, yet present in either zebrafish or medaka were subsequently added to the *ancient/very ancient* category despite being just outside of the above parameters. The same criteria were used to define sequence conservation. However, instead of requiring accessible-chromatin overlap, sequences were evaluated for having an aligned orthologous region in each species.

To visualize these peaks across species, we used the Integrative Genomics Viewer (IGV).^114^ For each species, RPKM-normalized read counts were used either directly (paralog displays) or summed across replicates and across developmental/diapause stages (for single displays) to create single coverage tracks for fish without diapause and two tracks (one diapause, one development) for fish with diapause. Tracks from each species were then anchored to each other via a single conserved base in the multiple-whole-genome-alignment and extended to the exact same window size in all species. The anchor point for each peak region was chosen based on its proximity to the summit of the peak in the African turquoise killifish. Track height for each species was set automatically by IGV using either the height of the peak of interest, or, in species without a conserved peak, to the height of the tallest peak within 40kb of the anchoring base pair. These visualizations illustrate the conservation and specialization states described above.

These analyses revealed that for the majority of peaks, the genome sequences under chromatin accessible peaks are ‘alignable’ (i.e. conserved enough to establish orthology at the genome-wide level), but chromatin accessibility at those regions evolved very recently and exclusively in the African turquoise killifish. This pattern was consistent for genome-wide chromatin, chromatin associated with singleton genes (Figures S3C and S3D). The sequence conservation is also strongest at coding sequence (exons) and decays as expected across promoters, UTRs, introns, and intergenic regions (Figure S3E).

#### ATAC-seq Principal Component Analysis (PCA)

To explore the global relationships between killifish ATAC-seq samples, we performed principal component analysis (PCA) using ATAC-seq peak intensities (normalized aligned read counts for each peak). To this end, we first generated peak intensity matrices for each of the following comparisons: 1) for the African turquoise killifish diapause and development samples (Figure 3B, upper-left); 2) for the South American killifish species (Figure 3B, upper-right), 3) for all killifish species (African turquoise killifish, South American killifish, lyretail killifish and red-striped killifish, Figure 3B, lower-left); 4) killifish with diapause (African turquoise killifish and South American killifish, Figure 3B, lower-right). For each comparison, the peak matrix contained VST-normalized peaks intensities for all consensus peaks detected in all the samples in that comparison. Cross-species comparison only included the peak(s) conserved in all samples. The total peaks used for PCA were 60,359 for the African turquoise killifish, 1,293 for all killifish, and 3,721 for killifish with diapause. PCA plots were done using autoplot command in ggfortify (version 0.4.11) package^115^ in R (version 3.6.2).

#### Diapause differential peak analysis

To identify ATAC-seq peaks that are specific to diapause in the African turquoise killifish genome, we performed a differential peak accessibility analysis pairwise between the two developmental conditions (pre-diapause and non-diapause) and the two diapause conditions (diapause at 6 days and 1 month time points) using DiffBind (version 2.16.2).^116,146^ We used both DESeq2^102^ and edgeR^117^ algorithms implemented in DiffBind for differential accessibility analysis. Diapause specific peaks were then identified as the peaks that were significantly up (chromatin more open) in any of the two diapause conditions with either DESeq2 or edgeR, but do not significantly change (up or down) between the two development conditions with both DESeq2 and edgeR. This led to 6,490 chromatin peaks genome-wide in African turquoise killifish and 6647 chromatin peaks genome-wide in South American killifish that are significantly up in diapause but do not change during development (Figure S3A; Tables S3B and S3C). Peaks were assigned to their nearest genes using ChIPseeker (version 1.28.3),^118^ to identify 1,880 diapause specific peaks at specialized paralogs in African turquoise killfish (Table S3B) and 8166 diapause specific peaks at specialized paralogs in South American killifish (Table S3C). Peak annotation with the genomic properties was also performed using ChIPseeker (Figure S3B). These peaks at specialized paralogs were used for motif enrichment and peak conservation analyses presented in Figures 3, 4, 5, and S3–S5.

#### Motif enrichment and conservation

HOMER (version 4.10), was used for transcription factor binding site enrichment analysis,^27^ using the ATAC-seq peaks that are significantly up in diapause and were in proximity to the diapause specific paralogs for the African turquoise killifish and their orthologous conserved peaks in other species. Genomes of all the species were added to HOMER using “*loadGenome.pl*” utility with the genome fasta and GFF files as input (Table S1A). We then used the genomic coordinates from the bed file for the diapause specific ATAC-seq peaks at paralogs as input to “*findMotifsGenome.pl*” and specified vertebrate motifs by “*-mset vertebrates*”. Known motifs in “*knownResults.txt*” generated by the HOMER output was used for all the analyses. To remove redundancy in motifs, we performed a motif clustering using tomtom utility in the MEME suit (version 5.3.0)^119,147^ using the following parameters: *-thresh 1e-5 –evalue -min-overlap 6*. The resulting clusters were manually curated, and motifs (binding sites) were assigned to the genes coding for the transcription factors.

#### TF binding sites across species

To assess the evolution and conservation of African turquoise killifish diapause-specific transcription factor binding sites at specialized paralogs in other species, we extracted sequences of these motifs from African turquoise killifish and the corresponding aligned sequences in other species from our 5-way multiple whole-genome alignment. We observed that a vast majority of transcription factor binding motifs that are enriched in ATAC-seq peaks up in diapause at specialized paralogs in the African turquoise killifish are aligned in other species with motif-like sequences (i.e. sequences similar to the canonical motifs). To assess if these motif-like sequences are likely to be bound by their respective transcription factors, we subjected motif or motif-like sequences to a binding likelihood calculation identical to that used by HOMER.^27^ We then determined if motif-like sequences in species other than African turquoise killifish met the log odds detection threshold (defined as the log(X_1_/0.25) + log(X_2_/0.25) +... log(X_n_/0.25) where X is the probability of a given base being present at a given location in a given motif) computed by HOMER^27^ during motif enrichment, which is used to determine likelihood of transcription factor bound vs. unbound sites. We also excluded motif sites in peaks where an identical motif was found near the aligned region in another species. This allowed us to detect cases where the sequence directly aligned to a motif is not conserved, but the motif is present nearby and possibly providing similar regulatory potential.

These analyses revealed that a very low number of motif-like sequences in other species are expected to bind the transcription factor at that position and can be considered as conserved transcription factor binding sites across species (4.77% on average). Thus, the vast majority of these motif-like sequences were likely used as ‘substrates’ during evolution for mutation and selection of canonical motif sequences for binding of transcription factors (Figures 5C, 5D, S5A, and S5B). The same approach was used to compare motifs in conserved accessible chromatin (Figure S4C).

To compare motif between the African and the South American killifish diapause in an alignment independent manner, we focused on diapause-specific accessible chromatin peaks at specialized paralogs in the two species. We performed two independent motif enrichment analyses using these peak sets independently and compared the significantly enriched motifs in at least one of the species (Figure S4D). To compare the convergent evolution of motifs in the African and the South American killifish in an alignment independent manner, we focused on the diapause-specific chromatin accessible peaks at specialized paralogs independently in the African and South American killifish. We then identified the one-to-one orthologs of these specialized genes in other killifish without diapause (lyretail killifish, red-striped killifish, medaka and zebrafish) and identified all the peaks closest to these ortholog genes. Because there is no diapause in these other killifish species, we down sampled these peaks to the same number as diapause-specialized peaks in the African turquoise killifish, keeping the same composition of peaks (e.g. promoter, intronic, intergenic etc.). We then performed the motif enrichment analysis and comparison in these peaks (Figure S4E).

To identify the subsets of paralogs controlled by each diapause-specific binding sites, we examined the subset of specialized paralog pairs that have a differentially accessible peak containing an enriched TF binding site (Figure S6D).

#### Transposable element analysis

To evaluate the contribution of Transposable Elements (TEs) for the evolution of diapause, we first developed a comprehensive map of abundance and genomic location of all TEs in the aforementioned teleost fish species used to construct the genome multi-alignment. We employed RepeatMasker (version 4.0)^120^ to identify repetitive sequences using the *Teleosti* suite of know repeat elements *<Repeatmasker* -*a* -*s -species ‘Teleostei’ Input.fa>* and *< processRepeats -xsmall* RMoutput.fa.gz> allowing for a standardized repetitive element set across species. We detected similar abundances of TE classes and families as previously reported by various sources.^148^ We then identified overlap between all ATAC-seq peak coordinates and TE coordinates in African turquoise killifish. We evaluated TE enrichment at ATAC-seq peaks up specifically in diapause as compared to: 1) ‘Genome’: TE representation genome-wide (Figure 5F, upper), 2) ‘Chromatin’: TE representation within all ATAC-seq peaks (Figure 5F, middle), 3) ‘Control loci’: size-matched regions 10kb downstream of ATAC-seq up specifically in diapause (Figure 5F, lower), using a binomial test (Mutational Patterns Package version 3.2.0).^121^ Several TE families showed enrichment specific to differentially accessible chromatin sites specific to diapause, such as Crypton-A (DNA), Zisupton (DNA), RTE-X (LINE), and tRNA-Mermaid (SINE) (Figure 5F).

We then evaluated the overlap between these TE instances and enriched transcription factor binding motifs detected in our analysis above. These chromatin-accessible TE-embedded motifs were also evaluated for conservation across species by assessing whether 1) the TE is present at aligned location in the genome alignment and contains the transcription factor binding motif sequence, 2) the TE is present at the aligned location in other species, but lacks the transcription factor binding motif sequence, 3) the TE is absent at the aligned location, but a transcription factor binding motif still exist at this location in the alignment, or 4) both the TE and transcription factor binding motif binding site are absent at the aligned location in the other species. This analysis revealed that a majority of TE sites are exclusive to African turquoise killifish, as can be expected given the rapid rate at which the TE landscape changes and given the recent TE expansion in the African turquoise killifish genome.^29,149^

#### Positive selection of regulatory regions

To evaluate whether diapause-accessible chromatin peaks show any signature of positive selection, we used a recently developed method to detect positive selection at transcription factor binding sites and accessible chromatin.^28,150^ We scanned for signature of positive selection at the genomic DNA underlying ATAC-seq peaks with respect to: 1) ancestor of all killifish species in our analysis (‘killifish ancestor’); and 2) ancestor of killifish and medaka (‘pre-medaka ancestor’) (Figure S6A). We first inferred ancestral sequences for these two nodes within the teleost lineage using the PAML package (version 4.8).^122^ Alignment blocks from our 5-way fish multiple whole-genome alignment that were at least 50bp long and covered at least 50% of the ATAC-seq peaks were used for the ancestor generation and positive selection analysis. We excluded ATAC-seq peaks that were in exons to focus on regulatory elements. The ancestral sequences and the African turquoise killifish sequences were used to generate Support Vector Machine (SVM) kmer weights and positive selection was detected using hightail test as recommended^28,150^ (https://github.com/ljljolinq1010/A-robust-method-for-detecting-positive-selection-on-regulatory-sequences/). The Benjamini-Hochberg procedure was used for multiple hypothesis correction, and ATAC-seq peaks with FDR < 0.1 for either pre-killifish or pre-medaka ancestors were considered to be under positive selection (Table S3B).

In total, we detected 3,836 and 3,928 ATAC-seq peaks with signature of positive selection using the ‘killifish ancestor’ and ‘pre-medaka ancestor’ inferred sequences respectively, with both having a strong overlap of 3,370 (76.7%) (Figures S6A and S6B). We used the union of the two groups for the downstream analysis. A total of 172 diapause-specific ATAC-seq peaks at specialized paralogs showed signature of positive selection (Figure 5E; Table S3B). These were enriched for several of the transcription factor binding motifs detected in our previous analysis, including REST, FOXO3 and PPARs (Figures 5E and S6C). The functional enrichment of ATAC-seq peaks also included several functions related to lipid metabolism (Table S4A). These results suggest that at least a portion of genomic loci underlying diapause-specific ATAC-seq peaks may have evolved due to positive selective pressure at these loci.

#### Positive selection on protein-coding genes

The protein-coding genes under positive selection in the African turquoise killifish were identified using phylogenetic analysis involving 19 fish species with and without diapause as described in Wagner et al.^9^ Briefly, protein sequences were clustered using Proteinortho (version 5.11),^123^ followed by filtering of clusters and alignment of coding sequences of the filtered clusters using PRANK v.140603.^124^ The resulting codon aware alignments were filtered with GUIDANCE v2.0^125^ to remove low quality regions. Proteins and individual amino acids under positive selection were then identified in either the ancestor of African killifish species with diapause (in the branch leading to the African killifish genus nothobranchius after separation from the African killifish without diapause *A. striatum*) or the branch leading to the African turquoise killifish only, using the branch-site model in CODEML implemented in the Phylogenetic Analysis by Maximum Likelihood package (PAML).^122^ Notably, the ancestral branch co-insides with the time period at which evolution of diapause likely occurred in African turquoise killifish (~18 mya). Proteins with a *P*-value of the branch-site test less than 0.05 (without any FDR correction to maximize the number of proteins with potential signals of selection) were then filtered. We used the union of proteins under positive selection identified using both the ancestral and the African turquoise killifish branch. This led to a list of 277 protein-coding genes under positive selection in the ancestor of killifish species with diapause after divergence from killifish species without diapause and outgroup fish species (Figure S1I).

#### Functional enrichment analysis

To perform functional enrichment analysis for diapause specific African turquoise killifish ATAC-seq peaks or upregulated genes in diapause, we used Gene Ontology (GO) analysis using GOstats package (version 2.56.0).^126^ GO terms from human and zebrafish were assigned to their killifish orthologs (best hit protein with BLASTp E-value >1e-3). For GO enrichment analysis using diapause specific ATAC-seq peaks, we used the non-redundant list of genes closest to the peaks (Table S3B) with all protein coding genes as background and performed a hypergeometric test implemented in GOstats. Similarly, for RNA-seq, we used genes upregulated in diapause (Table S1C). GO terms enriched in both diapause RNA-seq and ATAC-seq included many GO terms related to lipid metabolism (Tables S2A, S2B, S4A, and S7A). We also performed GO enrichment analysis for the subset of ATAC-seq peaks that show signatures of positive selection (see above, Table S4A), and observed that several lipid metabolism related functions are enriched in the genes next to the chromatin accessibility regions that have evolved under positive selection (Table S4A). For GO terms share across species, we performed independent enrichment analyses using the genes significantly upregulated during diapause in the African turquoise killifish, the South American killifish and mouse using the same approach (Table S2A).

To identify the upstream regulators of genes upregulated during diapause in the African turquoise killifish, we used Ingenuity Pathway Analysis (IPA) upstream regulator analysis (QIAGEN, March 2021 release) (Table S7B).

#### TF knockout selection

To select key transcription factors and test their functional role in the diapause program, we integrated both our ATAC-seq and RNA-seq data to generate a list of top candidates. A transcription factor was included in our list of top candidates if the binding site of this transcription factor was enriched in chromatin regions that become differentially accessible (e.g., ‘open chromatin’) during diapause (but not during development), and if the expression of this transcription factor was significantly higher in at least one diapause time-point compared to development. For each candidate, we verified that there was a clear ortholog between mammals and killifish. We then prioritized candidates, considering the novelty or conservation of a transcription factor for a role in a suspended animation phenotype as well as their connection to functions that could be relevant in diapause (e.g., lipid metabolism, stress response, etc.). We also included paralogs of the selected transcription factors, as they may target the same binding sites. Our final list had 6 candidates: REST, FOXO3a, FOXO3b, PPARAa, PPARAb, and PPARG (Table S5A). Other transcription factors such as NR2F2 and TEAD2 were not as strong candidates: while their binding sites are enriched in diapause-accessible chromatin, the *NR2F2* gene is actually downregulated in diapause and TEAD2 does not have a clear ortholog in killifish.

#### sgRNA design

We selected single guide RNAs (sgRNAs) for each of these genes using the CHOPCHOP^127^ online guide design platform (https://chopchop.cbu.uib.no/). For each gene, we selected 3 sgRNAs that fit 3 different criteria: 1) the sgRNA needed to target multiple exons in the beginning of the candidate gene to increase the chance for an early stop codon, 2) the sgRNA needed to have a high predicted cutting efficiency of above 60%, and 3) the sgRNA was predicted to have no off-target sites or no alternative sites within a hamming distance of 1 within the genome (Table S5B). These criteria were used generate the top three sgRNA candidates for each gene and the sequences were generated with canonical linker/Cas9 domain for a complete sgRNA (Table S5B). sgRNAs were then synthesized using Integrative DNA Technology’s (IDT) custom RNA-oligo ordering platform.

In addition to transcription factor targeting sgRNAs, we generated three GC content-balance sgRNAs whose sequence does not appear in the genome of the African turquoise killifish and is not within a hamming distance of 3 of any know genomic location (Table S5B). These scrambled sgRNAs were predicted to have no cutting sites and were synthesized using the same RNA-oligo generation service and gene-targeting guides.

#### Single embryo RNA-seq pipeline

We first trimmed the adaptors from raw sequencing FastQ files using Trim Galore (version 0.4.5) followed by read quality assessment using FastQC^103^ (version 0.11.9), and MultiQC (version 1.8).^104^ Adaptor trimmed files were aligned to the African turquoise killifish genome (Table S1A) using STAR (version 2.7.1a).^105^ For accurate assignment of reads across paralogs, we excluded all the reads that mapped to multiple locations in the genome, and we only kept reads that align uniquely to a single genomic locus with samtools (version 1.5) using “*samtools view -q255*” command.

#### Filtering of control and knockout RNA-seq

We used several criteria to filter uninformative RNA-seq libraries. For control libraries (non-injected [Wildtype] and scrambled sgRNAs [Scramble]), we used the results of Sanger-sequencing of embryo lysate described above for filtering. We removed the libraries that did not yield good sequences, as we could not evaluate genotypes; those represented a minority of samples (2 out of 58, e.g., 3.45%). Because no editing is expected in the control samples, we removed libraries for which the ICE knockout scores were predicted to be higher than the background expectation (>5% knockout prediction score in Wildtype/Scramble); these samples likely represent poor Sanger-sequencing quality or technical artifacts (Figure S6E). We also removed samples that had poor correlation with each other and likely represented technical differences between library preparation. These 3 filtering steps resulted 25 wildtype and 18 control samples, and this large number helps to overcome potential individual-to-individual variation.

For knockout libraries, we excluded libraries whose ICE knockout scores were below the cutoff for an expected ‘majority-knockout’ mosaic animal (<45% knockout prediction in Synthergo ICE for mutants) (Figure S6E). For knockout libraries, failure to generate Sanger-sequencing may also represent complex rearrangements, and we thus kept these samples for our final analysis. There were only 3 knockout samples (1 each for *REST* diapause, *REST* development and *FOXO3B* development) with a low correlation with other replicates. We did not filter them out, as the nature of mosaic knockout may lead to differences in transcriptional phenotypes, even when targeting the same gene. These filtering steps resulted in at least 3 samples per stage per genotype (minimum of 6 samples per knockout of transcription factor of interest). For these libraries, read counts were then assessed using ‘featureCounts’^142^ function in Subread package (version 2.0.1).^106^ Raw gene expression values were then normalized using DEseq2 (version 1.30.1).^102^ We further evaluated the status of mutations of the putative knockouts by assessing read pileup misalignment and split-read alignment at the cut sites in each mutant (example in Figure S6F) and their general expression patterns across samples (Table S5C).

#### TF knockout RNA-seq Analysis

PCA was performed on the normalized read counts for each library using DESeq2 (Figure 6B). In addition, to represent the effect that each gene knockout had on the transcriptome we identified DEGs between each condition in a pairwise manner between Wildtype (non-injected embryos), Scrambled (embryos injected with scrambled sgRNAs), and transcription factor knockout libraries (TF KO) for both diapause and development using DEseq2. No significant DEGs were detected between Scramble and Wildtype samples at FDR < 0.1 (after multiple hypothesis correction using Independent Hypothesis Weighting; IHW approach^151^). To eliminate the transcriptional impact of injection, we marked a gene as DEG if it was significantly differentially expressed at FDR < 0.1 in both “TF KO vs Scramble” and “TF KO vs Wildtype” samples (after multiple hypothesis correction using Independent Hypothesis Weighting; IHW approach). We use as Control the intersection of Wildtype and Scramble samples. There was no significant overlap between the DEGs for each TFKO and predicted target genes of each transcription factor as determined by our ATAC-seq data, indicating that all these genes may not be the direct targets of these transcription factors.

#### TF knockout correlation plots

To overlay the effect of gene knockouts on diapause, we assessed the correlation between i) the log fold-change of differentially expressed (DE) genes between control diapause and development and ii) the log fold-change of DE genes between gene knockout diapause and control diapause (Figure 6C). Each knockout was then assessed for its impact of the diapause transcriptional program: no effect (no correlation), accentuated diapause-like program (positive correlation), or switch to a more development-like program (negative correlation). Spearman’s ρ and *P*-values were calculated by the basic functions of R v3.6.2 (Figures 6D and 6E).

#### TF knockout GO enrichment

GO enrichment analysis for each TF-KO was performed using Gene Set Enrichment Analysis (GSEA) implemented in ClusterProfiler R package^152^ after ranking genes based on *significance* of enrichment defined as: -log_10_(P-value)*Fold Change (Figure 6F; Tables S6A–S6C).

#### TF knockout paralog pairs

Previously identified specialized paralog pairs were assessed for expression changes in the context of knockout embryos. The aggregated median expression distribution of diapause and development genes in each pair in Knockout samples were compared to their median expression in both Scrambled and Wildtype together (control). Degree of specialization (difference between diapause gene and development gene median expression) was different in the context of *REST* but not *FOXO3a* and *FOXO3b* knockout (Figures 6G and S6G, two-way ANOVA, *P* < 0.05).

#### Lipidomics analysis

Principal Component Analysis (PCA) was performed using all the lipids identified for: 1) African turquoise killifish diapause and development samples (Figure 7B); and 2) African turquoise and red-striped killifish pre-diapause samples (Figure S7C). The total of 431 filtered and normalized lipid intensities were used for PCA (see below), which were also plotted using autoplot function in ggfortify package (version 0.4.11) in R (version 4.0.5).

Discriminant analysis was performed using a Welch’s t-test that does not assume equal population variances for each lipid among the two diapause (6 days and 1 month) and the two development conditions (pre-diapause and diapause escape). Lipids that were significantly different (Welch’s t-test, *P* < 0.05 after multiple hypothesis correction using Benjamini-Hochberg method) between diapause and development but did not significantly change between the two development conditions were categorized as diapause specific lipids. These constitute lipids that go up or down when embryos enter diapause but do not change among the two development time points. This led to 350 diapause specific lipid changes, 80 of which were triglycerides, including very long chain fatty acid triglycerides (Figures 7C, 7D, S7B, and S7C; Table S7C).

#### Quantification of lipid droplets

To visualize lipid droplets in *N. furzeri*, embryos were collected using from the same mating cohorts used to generate CRISPR/Cas9-mediated knockout embryos for experiments described above (1 male to 3 females, ~1.5–3 months of age). Viable embryos were washed with ~1ml of embryo solution and then monitored until they had reached the proper stages of development and diapause. Embryos were segregated into four groups: 1) embryos in pre-diapause state (date of heartbeat onset) (Pre-Dia), 2) embryos in development (1 day post-heartbeat onset, heartbeat > ~85 beats per minutes) (Dev), 3) embryos in early diapause (6 days post heartbeat onset, heartbeat slowed to < ~20 beats per minutes) (Dia (6d)), and 4) embryos in late diapause (1 month post heartbeat onset, heartbeat slowed to < ~20 beats per minute) (Dia (1m)). Embryos were processed to visualize lipid droplets by staining with the neutral lipid dye BODIPY^™^ 493/503 (D3992, Invitrogen). Embryos were dissected at 4◦ C in 1x phosphate buffered saline (PBS), removing the chorion and embryonic membrane so the embryo body could be isolated. Following dissection, embryos were placed in a 9-well 1ml glass plate (PYREX^™^ Spot plate), with each well containing 5–7 embryo for the same condition. Embryos were fixed in ~1ml of freshly diluted 4% paraformaldehyde (PFA) (28906, Thermo Scientific) for 1 hour at room temperature, followed by three wash steps in ~1ml PBS to remove residual PFA. To stain for lipid droplets, embryos were incubated for 30 minutes at room temperature in the dark with 1.5μg/ml BODIPY^™^ 493/503 (D3992, Invitrogen) in ~1ml PBS. Embryos were washed with ~1ml PBS once to remove residual dye, mounted on a 2% agarose pad and covered with a glass cover slide using spacers for imaging.

## Supplementary Material

Supplemental

## Figures and Tables

**Table:** KEY RESOURCES TABLE

REAGENT or RESOURCE	SOURCE	IDENTIFIER

Chemicals, Peptides, and Recombinant Proteins		

EquiSPLASH LIPIDOMIX	Avanti Polar Lipids	Cat. No.330731
d17-Oleic acid	Cayman chemicals	Cat. No.9000432
Alt-R^™^ S.p. Cas9 Nuclease	Integrated DNA Technologies (IDT)	Cat. No. 1081058
BODIPY^™^ 493/503	Invitrogen	Cat. No. D3992
Otohime C1 Fish Pellets	Reed Mariculture	N/A
Otohime Ep1 Fish Pellets	Reed Mariculture	N/A
Ringers Solution	Sigma-Aldrich	Cat. No. 96724
Methylene Blue	Sigma-Aldrich	Cat. No. 319112
EZ-lysis buffer	Sigma-Aldrich	Cat. No.3408
Methyl tert-butyl ether (MTBE)	Sigma-Aldrich	Cat. No. 316466
Methanol	Sigma-Aldrich	Cat. No. MX0482
Toluene	Sigma-Aldrich	Cat. No. 244511
Phosphate Buffered Saline (PBS)	ThermoFisher	Cat. No. AM9624
Trypan blue stain	ThermoFisher	Cat. No.15250061
Nuclease-free water	ThermoFisher	Cat. No.AM9916
2X DreamTaq PCR Master Mix	ThermoFisher	Cat. No. K1081
RNase Zap^™^ RNase Decontamination Solution	ThermoFisher	Cat. No. AM9780

Critical Commercial Assays		

Agilent’s High Sensitivity DNA Kit	Agilent	Cat. No. 5067–4626
Nextera XT DNA Library Prep Kit	Illumina	Cat. No. FC-131–1096
Tn5 Transposition DNA Buffer and Enzyme kit	Illumina	Cat. No. 20034197
High Yield Short Read Nextseq Kit	Illumina	Cat. No. PE-410–1001
BCA Protein Assay Kit	Pierce	Cat. No.23225
Mini-elute kit	Qiagen	Cat. No. 28206
SMART-Seq^®^ v4 Ultra^®^ Low Input RNA Kit	Takara	Cat. No. 634890

Deposited Data		

Raw and analyzed data	This paper	GSE185817
Lipidomics data	This paper	ST001898
Additional African turquoise killifish RNA-seq data	Hu et al.^6^	PRJNA503701
African turquoise killifish reference genome: Nfu_20140520	Reichwald et al.^8^	https://www.ncbi.nlm.nih.gov/datasets/genome/GCF_001465895.1/
South American killifish diapause RNA-seq data	Wagner et al.^9^	PRJNA272154
South American killifish reference genome: Austrofundulus_limnaeus-1.0	Wagner et al.^9^	https://0-www-ncbi-nlm-nih-gov.brum.beds.ac.uk/datasets/genome/GCF_001266775.1/
Mouse RNA-seq data	Hussien et al.^22^	GSE143494
Medaka ATAC-seq data	Marlétaz et al.^24^	GSE106428
Zebrafish ATAC-seq data	Marlétaz et al.^24^	GSE106428
Zebrafish late development RNA-seq data	Marlétaz et al.^24^	GSE106430
Medaka developmental RNA-seq data	Marlétaz et al.^24^	GSE106430
Lyretail killifish reference genome: MPIBA_Aaus_1.0	Cui et al.^29^	https://www.ncbi.nlm.nih.gov/nuccore/SSNS00000000.1/
South American killifish developmental RNA-seq data	Romney et al.^73^	PRJNA272154
Zebrafish early developmental RNA-seq data	Pauli et al.^96^	GSE32898
Mouse reference genome: GCF_000001635.20 (mm10)	Church et al.^97^	https://www.ncbi.nlm.nih.gov/datasets/genome/GCF_000002035.6/
Medaka reference genome: ASM223467v1	Ichikawa et al.^98^	https://www.ncbi.nlm.nih.gov/datasets/genome/GCF_002234675.1/
Zebrafish reference genome: GRCz11	Howe et al.^99^	https://www.ncbi.nlm.nih.gov/datasets/genome/GCF_002234675.1/

Experimental Models: Organisms/Strains		

*Nothobranchius furzeri*	Brunet Lab	GRZ strain
Brine Shrimp Eggs	Brine Shrimp Direct	Cat. No. 454GR
*Austrofundulus limnaeus*	Podrabsky Lab	Quisiro strain
*Aphyosemion australe*	Wetspot Tropical Fish	Gold strain
*Aphyosemion striatum*	Wetspot Tropical Fish	Aquarium strain

Oligonucleotides		

Primers for *REST*, *FOXO3a*, *FOXO3b*,	This paper	N/A
*PPARAa*, *PPARAb*, and *PPARG*, see Table S5B		
sgRNA sequences for *REST*, *FOXO3a*,	This paper	N/A
*FOXO3b*, *PPARAa*, *PPARAb*, and *PPARG*, see Table S5B		

Software and Algorithms		

Original code generated for the study.	This paper	https://github.com/SinghLabUCSF/Diapause-multiomics
Picard Tools v2.22.1	Broad Institute	https://broadinstitute.github.io/picard/
SnapGene v7.0	Dotmatic	https://www.snapgene.com/
TrimGalore v0.4.1	Felix Krueger	https://www.bioinformatics.babraham.ac.uk/projects/trimgalore/
Fiji v2.0.0-rc-68/1.52h	FijiTeam	https://fiji.sc/
Ingenuity Pathway Analysis (IPA)	QIAGEN	https://digitalinsights.qiagen.com/products-overview/discovery-insights-portfolio/analysis-and-visualization/Qiagen-ipa/
R v3.6.2	R core team	https://www.r-project.org/
ICE Analysis v.1.0	Synthego	https://ice.synthego.com/#/
LipidSearch v4.2.21	ThermoFisher	https://www.thermofisher.com/us/en/home/industrial/mass-spectrometry/liquid-chromatography-mass-spectrometry-lc-ms/lc-ms-software/multi-omics-data-analysis/lipid-search-software.html
Zen Blue v3.4.0	Zeiss	https://www.zeiss.com/microscopy/en/products/software/zeiss-zen.html#zenversions
HOMER v4.10	Heinz et al.^27^	http://homer.ucsd.edu/homer/
OrthoFinder v2.5.4	Emms and Kelly^100^	https://github.com/davidemms/OrthoFinder
BLASTp v2.7.1+	Altschul et al.^101^	https://blast.ncbi.nlm.nih.gov/Blast.cgi?PAGE=Proteins
DEseq2 v1.30.1	Love et al.^102^	https://bioconductor.org/packages/release/bioc/html/DESeq2.html
FastQC v0.11.9	Andrew^103^	http://www.bioinformatics.babraham.ac.uk/projects/fastqc
MultiQC v1.8	Ewels et al.^104^	https://multiqc.info
STAR v2.7.1a	Dobin et al.^105^	https://github.com/alexdobin/STAR
Subread v2.0.1	Liao et al.^106^	https://sourceforge.net/projects/subread/
BowTie2 v2.2.5	Langmead and Salzberg^107^	http://bowtie-bio.sourceforge.net/bowtie2/index.shtml
Samtools v1.5	Danecek et al.^108^	http://www.htslib.org/
deepTools v3.2.1	Ramirez et al.^109^	https://deeptools.readthedocs.io/en/develop/
MACS2	Zhang et al.^110^	https://pypi.org/project/MACS2/#description
LASTz v1.04.00	Harris^111^	https://www.bx.psu.edu/rsharris/lastz/
UCSC genome utilities v15.6.0	Kuhn et al.^112^	http://hgdownload.soe.ucsc.edu/admin/exe/
Multic/TBA v10	Blanchette et al.^113^	https://www.bx.psu.edu/miller_lab/
Integrative Genomics Viewer (IGV) v2.4.18	Robinson et al.^114^	https://software.broadinstitute.org/software/igv/
ggfortify v0.4.11	Tang and Masaaki^115^	https://cran.r-project.org/web/packages/ggfortify/index.html
DiffBind v2.16.2	Stark and Brown^116^	https://hbctraining.github.io/Intro-to-ChIPseq/lessons/08_diffbind_differential_peaks.html
EdgeR v4.0.16	Robinson et al.^117^	https://bioconductor.org/packages/release/bioc/html/edgeR.html
CHIPseeker v1.28.3	Yu et al.^118^	https://bioconductor.org/packages/release/bioc/html/ChIPseeker.html
MEME suit v5.3.0	Bailey et al.^119^	https://meme-suite.org/meme/doc/download.html
RepeatMasker v4.0	Smit et al.^120^	https://www.repeatmasker.org/
Mutational Patterns Package v3.2.0	Blokzjl et al.^121^	https://bioconductor.org/packages/release/bioc/html/MutationalPatterns.html
PAML v4.8	Yang^122^	http://abacus.gene.ucl.ac.uk/software/paml.html
Proteinortho v5.11	Lechner et al.^123^	https://www.bioinf.uni-leipzig.de/Software/proteinortho/
PRANK v.140603	Loytynoja^124^	https://ariloytynoja.github.io/prank-msa/
GUIDANCE v2.0	Sela et al.^125^	http://guidance.tau.ac.il/source.php
GOstats package v2.56.0	Falcon and Gentleman^126^	https://bioconductor.org/packages/release/bioc/html/GOstats.html
CHOPCHOP v3.0.0	Labun et al.^127^	https://chopchop.cbu.uib.no/

Other		

Mini-douncers	DWK (Kimble)	Cat. No.885300–0000
Biological-grade tweezers	Electron Microscopy Sciences	Cat. No. 72700-D
1.5ml Eppendorf^™^ DNA loBind microcentrifuge tubes	Eppendorf	Cat. No. 13-698-791
PYREX^™^ Spot Plate	Fisher Scientific	Cat. No. 13–748B
Extra Coarse Glass Beads (30/40 Mesh, 425–560micron size)	Kramer Industries Inc.	N/A
FastPrep^®^ −24 homogenizer	MB Biomedicals	Cat. No. 116004500
Zirconia/Silicon 0.5mm glass beads	Research Products International Corp	Cat. No. 9834
Accucore C30 column 2.1×150mm, 2.6μm	ThermoFisher	Cat. No. TFS-27826-152130
LSM 900 Airyscan SR Confocal Microscope	Zeiss	N/A

## Highlights

- Paralogs that specialize for expression in diapause are evolutionarily very ancient
- The chromatin landscape at very ancient paralogs was recently rewired in diapause
- Rewiring of the chromatin landscape reveals binding sites for key transcription factors
- A central function affected by rewiring is lipid metabolism, which is unique in diapause

## References

[1] Cellerino A, Valenzano DR, and Reichard M. (2016). From the bush to the bench: the annual Nothobranchius fishes as a new model system in biology. Biol. Rev. Camb. Philos. Soc 91, 511–533. 10.1111/brv.12183. 25923786

[2] Platzer M., and Englert C. (2016). Nothobranchius furzeri: A Model for Aging Research and More. Trends Genet. 32, 543–552. 10.1016/j.tig.2016.06.006. 27427533

[3] Vrtílek M, Žák J, Pšenička M, and Reichard M. (2018). Extremely rapid maturation of a wild African annual fish. Curr. Biol 28, R822–R824. 10.1016/j.cub.2018.06.031. 30086311

[4] Hu CK, and Brunet A. (2018). The African turquoise killifish: A research organism to study vertebrate aging and diapause. Aging Cell 17, e12757. 10.1111/acel.12757. 29573324 PMC5946070

[5] Reichard M, and Polačik M. (2019). Nothobranchius furzeri, an ‘instant’ fish from an ephemeral habitat. eLife 8, e41548. 10.7554/eLife.41548. 30616713 PMC6324871

[6] Hu CK, Wang W, Brind’Amour J, Singh PP, Reeves GA, Lorincz MC, Alvarado AS, and Brunet A. (2020). Vertebrate diapause preserves organisms long term through Polycomb complex members. Science 367, 870–874. 10.1126/science.aaw2601. 32079766 PMC7532943

[7] Renfree MB, and Fenelon JC (2017). The enigma of embryonic diapause. Development 144, 3199–3210. 10.1242/dev.148213. 28928280

[8] Reichwald K, Petzold A, Koch P, Downie BR, Hartmann N, Pietsch S, Baumgart M, Chalopin D, Felder M, Bens M, (2015). Insights into sex chromosome evolution and aging from the genome of a short-lived fish. Cell 163, 1527–1538. 10.1016/j.cell.2015.10.071. 26638077

[9] Wagner JT, Singh PP, Romney AL, Riggs CL, Minx P, Woll SC, Roush J, Warren WC, Brunet A, and Podrabsky JE (2018). The genome of Austrofundulus limnaeus offers insights into extreme vertebrate stress tolerance and embryonic development. BMC Genomics 19, 155. 10.1186/s12864-018-4539-7. 29463212 PMC5819677

[10] Koonin EV (2005). Orthologs, paralogs, and evolutionary genomics. Annu. Rev. Genet 39, 309–338. 10.1146/annurev.genet.39.073003.114725. 16285863

[11] Chen S, Krinsky BH, and Long M. (2013). New genes as drivers of phenotypic evolution. Nat. Rev. Genet 14, 645–660. 10.1038/nrg3521. 23949544 PMC4236023

[12] Ohno S, Wolf U, and Atkin NB (1968). Evolution from fish to mammals by gene duplication. Hereditas 59, 169–187. 10.1111/j.1601-5223.1968.tb02169.x. 5662632

[13] Conant GC, and Wolfe KH (2008). Turning a hobby into a job: how duplicated genes find new functions. Nat. Rev. Genet 9, 938–950. 10.1038/nrg2482. 19015656

[14] Cui R, Tyers AM, Malubhoy ZJ, Wisotsky S, Valdesalici S, Henriette E, Kosakovsky Pond SL, and Valenzano DR (2021). Ancestral transoceanic colonization and recent population reduction in a nonannual killifish from the Seychelles archipelago. Mol. Ecol 30, 3610–3623. 10.1111/mec.15982. 33998095

[15] Emms DM, and Kelly S. (2019). OrthoFinder: phylogenetic orthology inference for comparative genomics. Genome Biol. 20, 238. 10.1186/s13059-019-1832-y. 31727128 PMC6857279

[16] Lan X, and Pritchard JK (2016). Coregulation of tandem duplicate genes slows evolution of subfunctionalization in mammals. Science 352, 1009–1013. 10.1126/science.aad8411. 27199432 PMC5182070

[17] Murphy WJ, and Collier GE (1997). A molecular phylogeny for aplocheiloid fishes (Atherinomorpha, Cyprinodontiformes): the role of vicariance and the origins of annualism. Mol. Biol. Evol 14, 790–799. 10.1093/oxfordjournals.molbev.a025819. 9254916

[18] Furness AI, Reznick DN, Springer MS, and Meredith RW (2015). Convergent evolution of alternative developmental trajectories associated with diapause in African and South American killifish. Proc. Biol. Sci 282, 20142189. 10.1098/rspb.2014.2189. 25631993 PMC4344141

[19] Martin KLM, and Podrabsky JE (2017). Hit pause: developmental arrest in annual killifishes and their close relatives. Dev. Dyn 246, 858–866. 10.1002/dvdy.24507. 28407437

[20] Podrabsky JE, and Arezo M. (2017). Annual killifishes as model systems for advancing understanding of evolution and developmental biology. Dev. Dyn 246, 778. 10.1002/dvdy.24594. 29044855

[21] Fenelon JC, and Renfree MB (2018). The history of the discovery of embryonic diapause in mammals. Biol. Reprod 99, 242–251. 10.1093/biolre/ioy112. 29741586

[22] Hussein AM, Wang Y, Mathieu J, Margaretha L, Song C, Jones DC, Cavanaugh C, Miklas JW, Mahen E, Showalter MR, (2020). Metabolic control over mTOR-dependent diapause-like state. Dev. Cell 52, 236–250.e7. 10.1016/j.devcel.2019.12.018. 31991105 PMC7204393

[23] Buenrostro JD, Giresi PG, Zaba LC, Chang HY, and Greenleaf WJ (2013). Transposition of native chromatin for fast and sensitive epigenomic profiling of open chromatin, DNA-binding proteins and nucleosome position. Nat. Methods 10, 1213–1218. 10.1038/nmeth.2688. 24097267 PMC3959825

[24] Marlétaz F, Firbas PN, Maeso I, Tena JJ, Bogdanovic O, Perry M, Wyatt CDR, de la Calle-Mustienes E, Bertrand S, Burguera D, (2018). Amphioxus functional genomics and the origins of vertebrate gene regulation. Nature 564, 64–70. 10.1038/s41586-018-0734-6. 30464347 PMC6292497

[25] Rebeiz M, and Tsiantis M. (2017). Enhancer evolution and the origins of morphological novelty. Curr. Opin. Genet. Dev 45, 115–123. 10.1016/j.gde.2017.04.006. 28527813 PMC5623808

[26] Chuong EB, Elde NC, and Feschotte C. (2017). Regulatory activities of transposable elements: from conflicts to benefits. Nat. Rev. Genet 18, 71–86. 10.1038/nrg.2016.139. 27867194 PMC5498291

[27] Heinz S, Benner C, Spann N, Bertolino E, Lin YC, Laslo P, Cheng JX, Murre C, Singh H, and Glass CK (2010). Simple combinations of lineage-determining transcription factors prime cis-regulatory elements required for macrophage and B cell identities. Mol. Cell 38, 576–589. 10.1016/j.molcel.2010.05.004. 20513432 PMC2898526

[28] Liu J, and Robinson-Rechavi M. (2020). Robust inference of positive selection on regulatory sequences in the human brain. Sci. Adv 6, eabc9863. 10.1126/sciadv.abc9863. PMC7695467 33246961

[29] Cui R, Medeiros T, Willemsen D, Iasi LNM, Collier GE, Graef M, Reichard M, and Valenzano DR (2019). Relaxed selection limits life-span by increasing mutation load. Cell 178, 385–399.e20. 10.1016/j.cell.2019.06.004. 31257025

[30] Lu T, Aron L, Zullo J, Pan Y, Kim H, Chen Y, Yang TH, Kim HM, Drake D, Liu XS, (2014). REST and stress resistance in ageing and Alzheimer’s disease. Nature 507, 448–454. 10.1038/nature13163. 24670762 PMC4110979

[31] Zhao Y, Zhu M, Yu Y, Qiu L, Zhang Y, He L, and Zhang J. (2017). Brain REST/NRSF is not only a silent repressor but also an active protector. Mol. Neurobiol 54, 541–550. 10.1007/s12035-015-9658-4. 26742529

[32] Zullo JM, Drake D, Aron L, O’Hern P, Dhamne SC, Davidsohn N, Mao CA, Klein WH, Rotenberg A, Bennett DA, (2019). Regulation of lifespan by neural excitation and REST. Nature 574, 359–364. 10.1038/s41586-019-1647-8. 31619788 PMC6893853

[33] Wang L, Zhu X, Sun X, Yang X, Chang X, Xia M, Lu Y, Xia P, Yan H, Bian H, and Gao X. (2019). FoxO3 regulates hepatic triglyceride metabolism via modulation of the expression of sterol regulatory-element binding protein 1c. Lipids Health Dis. 18, 197. 10.1186/s12944-019-1132-2. 31729980 PMC6857156

[34] Zečić A, and Braeckman BP (2020). DAF-16/FoxO in Caenorhabditis elegans and its role in metabolic remodeling. Cells 9, 109. 10.3390/cells9010109. 31906434 PMC7017163

[35] Salih DAM, and Brunet A. (2008). FoxO transcription factors in the maintenance of cellular homeostasis during aging. Curr. Opin. Cell Biol. 20, 126–136. 10.1016/j.ceb.2008.02.005. 18394876 PMC2387118

[36] Liu T., Zimmerman KK., and Patterson GI. (2004). Regulation of signaling genes by TGFbeta during entry into dauer diapause in C. elegans. BMC Dev. Biol 4, 11. 10.1186/1471-213X-4-11. 15380030 PMC524168

[37] Sim C, and Denlinger DL (2008). Insulin signaling and FOXO regulate the overwintering diapause of the mosquito Culex pipiens. Proc. Natl. Acad. Sci. USA 105, 6777–6781. 10.1073/pnas.0802067105. 18448677 PMC2373331

[38] Zhang XS, Wang ZH, Li WS, and Xu WH (2022). FoxO induces pupal diapause by decreasing TGFbeta signaling. Proc. Natl. Acad. Sci. USA 119, e2210404119. 10.1073/pnas.2210404119. 36442095 PMC9894235

[39] Harmon GS, Lam MT, and Glass CK (2011). PPARs and lipid ligands in inflammation and metabolism. Chem. Rev 111, 6321–6340. 10.1021/cr2001355. 21988241 PMC3437919

[40] Lee CH, Olson P, and Evans RM (2003). Minireview: lipid metabolism, metabolic diseases, and peroxisome proliferator-activated receptors. Endocrinology 144, 2201–2207. 10.1210/en.2003-0288. 12746275

[41] Hwang JY, and Zukin RS (2018). REST, a master transcriptional regulator in neurodegenerative disease. Curr. Opin. Neurobiol 48, 193–200. 10.1016/j.conb.2017.12.008. 29351877 PMC5892838

[42] Prestigio C, Ferrante D, Marte A, Romei A, Lignani G, Onofri F, Valente P, Benfenati F, and Baldelli P. (2021). REST/NRSF drives homeostatic plasticity of inhibitory synapses in a target-dependent fashion. eLife 10, e69058. 10.7554/eLife.69058. 34855580 PMC8639147

[43] Kousteni S. (2012). FoxO1, the transcriptional chief of staff of energy metabolism. Bone 50, 437–443. 10.1016/j.bone.2011.06.034. 21816244 PMC3228887

[44] Almaida-Pagan PF, Ortega-Sabater C, Lucas-Sanchez A, Gonzalez-Silvera D, Martinez-Nicolas A, Rol de Lama MA, Mendiola P, and de Costa J. (2019). Age-related changes in mitochondrial membrane composition of Nothobranchius furzeri.: comparison with a longer-living Nothobranchius species. Biogerontology 20, 83–92. 10.1007/s10522-018-9778-0. 30306289

[45] Dabrowski R, Ripa R, Latza C, Annibal A, and Antebi A. (2020). Optimization of mass spectrometry settings for steroidomic analysis in young and old killifish. Anal. Bioanal. Chem 412, 4089–4099. 10.1007/s00216-020-02640-6. 32333075 PMC7320053

[46] Zajic DE, and Podrabsky JE (2020). Metabolomics analysis of annual killifish (Austrofundulus limnaeus) embryos during aerial dehydration stress. Physiol. Genomics 52, 408–422. 10.1152/physiol-genomics.00072.2020. 32776802

[47] Nakamura MT, Yudell BE, and Loor JJ (2014). Regulation of energy metabolism by long-chain fatty acids. Prog. Lipid Res 53, 124–144. 10.1016/j.plipres.2013.12.001. 24362249

[48] Wang CW (2016). Lipid droplets, lipophagy, and beyond. Biochim. Biophys. Acta 1861, 793–805. 10.1016/j.bbalip.2015.12.010. 26713677

[49] Elle IC, Olsen LCB, Pultz D, Rødkaer SV, and Faergeman NJ (2010). Something worth dyeing for: molecular tools for the dissection of lipid metabolism in Caenorhabditis elegans. FEBS Lett. 584, 2183–2193. 10.1016/j.febslet.2010.03.046. 20371247

[50] Sindelar PJ, Guan Z, Dallner G, and Ernster L. (1999). The protective role of plasmalogens in iron-induced lipid peroxidation. Free Radic. Biol. Med 26, 318–324. 10.1016/s0891-5849(98)00221-4. 9895222

[51] Braverman NE, and Moser AB (2012). Functions of plasmalogen lipids in health and disease. Biochim. Biophys. Acta 1822, 1442–1452. 10.1016/j.bbadis.2012.05.008. 22627108

[52] Paul S, Lancaster GI, and Meikle PJ (2019). Plasmalogens: A potential therapeutic target for neurodegenerative and cardiometabolic disease. Prog. Lipid Res 74, 186–195. 10.1016/j.plipres.2019.04.003. 30974122

[53] Wu RS, Lam II, Clay H, Duong DN, Deo RC, and Coughlin SR (2018). A rapid method for directed gene knockout for screening in G0 zebrafish. Dev. Cell 46, 112–125.e4. 10.1016/j.devcel.2018.06.003. 29974860

[54] Kroll F, Powell GT, Ghosh M, Gestri G, Antinucci P, Hearn TJ, Tunbak H, Lim S, Dennis HW, Fernandez JM, (2021). A simple and effective F0 knockout method for rapid screening of behaviour and other complex phenotypes. eLife 10, e59683. 10.7554/eLife.59683. 33416493 PMC7793621

[55] Long HK, Prescott SL, and Wysocka J. (2016). Ever-changing landscapes: transcriptional enhancers in development and evolution. Cell 167, 1170–1187. 10.1016/j.cell.2016.09.018. 27863239 PMC5123704

[56] Villar D, Berthelot C, Aldridge S, Rayner TF, Lukk M, Pignatelli M, Park TJ, Deaville R, Erichsen JT, Jasinska AJ, (2015). Enhancer evolution across 20 mammalian species. Cell 160, 554–566. 10.1016/j.cell.2015.01.006. 25635462 PMC4313353

[57] Monteiro A, and Podlaha O. (2009). Wings, horns, and butterfly eyespots: how do complex traits evolve? PLoS Biol. 7, e37. 10.1371/journal.pbio.1000037. 19243218 PMC2652386

[58] Guschanski K, Warnefors M, and Kaessmann H. (2017). The evolution of duplicate gene expression in mammalian organs. Genome Res. 27, 1461–1474. 10.1101/gr.215566.116. 28743766 PMC5580707

[59] Arnegard ME, Zwickl DJ, Lu Y, and Zakon HH (2010). Old gene duplication facilitates origin and diversification of an innovative communication system–twice. Proc. Natl. Acad. Sci. USA 107, 22172–22177. 10.1073/pnas.1011803107. 21127261 PMC3009798

[60] Knox K, and Baker JC (2008). Genomic evolution of the placenta using co-option and duplication and divergence. Genome Res. 18, 695–705. 10.1101/gr.071407.107. 18340042 PMC2336813

[61] Doherty A, and de Magalhães JP (2016). Has gene duplication impacted the evolution of Eutherian longevity? Aging Cell 15, 978–980. 10.1111/acel.12503. 27378378 PMC5013011

[62] Vazquez JM, and Lynch VJ (2021). Pervasive duplication of tumor suppressors in Afrotherians during the evolution of large bodies and reduced cancer risk. eLife 10, e65041. 10.7554/eLife.65041. 33513090 PMC7952090

[63] Tejada-Martinez D, de Magalhães JP, and Opazo JC (2021). Positive selection and gene duplications in tumour suppressor genes reveal clues about how cetaceans resist cancer. Proc. Biol. Sci 288, 20202592. 10.1098/rspb.2020.2592. 33622125 PMC7935004

[64] Jones FC, Grabherr MG, Chan YF, Russell P, Mauceli E, Johnson J, Swofford R, Pirun M, Zody MC, White S, (2012). The genomic basis of adaptive evolution in threespine sticklebacks. Nature 484, 55–61. 10.1038/nature10944. 22481358 PMC3322419

[65] Villar D, Flicek P, and Odom DT (2014). Evolution of transcription factor binding in metazoans - mechanisms and functional implications. Nat. Rev. Genet 15, 221–233. 10.1038/nrg3481. 24590227 PMC4175440

[66] Vierstra J, Rynes E, Sandstrom R, Zhang M, Canfield T, Hansen RS, Stehling-Sun S, Sabo PJ, Byron R, Humbert R, (2014). Mouse regulatory DNA landscapes reveal global principles of cis-regulatory evolution. Science 346, 1007–1012. 10.1126/science.1246426. 25411453 PMC4337786

[67] Indjeian VB, Kingman GA, Jones FC, Guenther CA, Grimwood J, Schmutz J, Myers RM, and Kingsley DM (2016). Evolving new skeletal traits by cis-regulatory changes in bone morphogenetic proteins. Cell 164, 45–56. 10.1016/j.cell.2015.12.007. 26774823 PMC4759241

[68] Verta JP, and Jones FC (2019). Predominance of cis-regulatory changes in parallel expression divergence of sticklebacks. eLife 8, e43785. 10.7554/eLife.43785. 31090544 PMC6550882

[69] Sundaram V, and Wysocka J. (2020). Transposable elements as a potent source of diverse cis-regulatory sequences in mammalian genomes. Philos. Trans. R. Soc. Lond. B Biol. Sci 375, 20190347. 10.1098/rstb.2019.0347. 32075564 PMC7061989

[70] Wang W, Hu CK, Zeng A, Alegre D, Hu D, Gotting K, Ortega Granillo A, Wang Y, Robb S, Schnittker R, (2020). Changes in regeneration-responsive enhancers shape regenerative capacities in vertebrates. Science 369, eaaz3090. 10.1126/science.aaz3090. PMC9479427 32883834

[71] Vrtílek M, Polačik M, and Reichard M. (2017). The role of energetic reserves during embryonic development of an annual killifish. Dev. Dyn 246, 838–847. 10.1002/dvdy.24528. 28598522

[72] Olsen L, Thum E, and Rohner N. (2021). Lipid metabolism in adaptation to extreme nutritional challenges. Dev. Cell 56, 1417–1429. 10.1016/j.devcel.2021.02.024. 33730548

[73] Romney ALT, Davis EM, Corona MM, Wagner JT, and Podrabsky JE (2018). Temperature-dependent vitamin D signaling regulates developmental trajectory associated with diapause in an annual killifish. Proc. Natl. Acad. Sci. USA 115, 12763–12768. 10.1073/pnas.1804590115. 30446615 PMC6294931

[74] Antebi A, Yeh WH, Tait D, Hedgecock EM, and Riddle DL (2000). daf-12 encodes a nuclear receptor that regulates the dauer diapause and developmental age in C. elegans. Genes Dev. 14, 1512–1527. 10.1101/gad.14.12.1512. 10859169 PMC316684

[75] Gerisch B, Weitzel C, Kober-Eisermann C, Rottiers V, and Antebi A. (2001). A hormonal signaling pathway influencing C. elegans metabolism, reproductive development, and life span. Dev. Cell 1, 841–851. 10.1016/s1534-5807(01)00085-5. 11740945

[76] Kenyon C, Chang J, Gensch E, Rudner A, and Tabtiang R. (1993). A C. elegans mutant that lives twice as long as wild type. Nature 366, 461–464. 10.1038/366461a0. 8247153

[77] Larsen PL, Albert PS, and Riddle DL (1995). Genes that regulate both development and longevity in Caenorhabditis elegans. Genetics 139, 1567–1583. 10.1093/genetics/139.4.1567. 7789761 PMC1206485

[78] Gottlieb S, and Ruvkun G. (1994). daf-2, daf-16 and daf-23: genetically interacting genes controlling Dauer formation in Caenorhabditis elegans. Genetics 137, 107–120. 10.1093/genetics/137.1.107. 8056303 PMC1205929

[79] Nelson CJ, Otis JP, and Carey HV (2009). A role for nuclear receptors in mammalian hibernation. J. Physiol 587, 1863–1870. 10.1113/jphysiol.2008.167692. 19289545 PMC2689326

[80] Arena R, Bisogno S, Gąsior q., Rudnicka J, Bernhardt Ł, Haaf T, Zacchini F, Bochenek M, Fic K, Bik E, (2021). Lipid droplets in mammalian eggs are utilized during embryonic diapause. Proc. Natl. Acad. Sci. USA 118, e2018362118. 10.1073/pnas.2018362118. 33649221 PMC7958255

[81] van der Weijden VA, Stötzel M, Iyer DP, Fauler B, Gralinska E, Shahraz M, Meierhofer D, Vingron M, Rulands S, Alexandrov T, (2024). FOXO1-mediated lipid metabolism maintains mammalian embryos in dormancy. Nat. Cell Biol. 26, 181–193. 10.1038/s41556-023-01325-3. 38177284 PMC10866708

[82] Jansen HT, Trojahn S, Saxton MW, Quackenbush CR, Evans Hutzenbiler BD, Nelson OL, Cornejo OE, Robbins CT, and Kelley JL (2019). Hibernation induces widespread transcriptional remodeling in metabolic tissues of the grizzly bear. Commun. Biol 2, 336. 10.1038/s42003-019-0574-4. 31531397 PMC6744400

[83] Xu Y, Shao C, Fedorov VB, Goropashnaya AV, Barnes BM, and Yan J. (2013). Molecular signatures of mammalian hibernation: comparisons with alternative phenotypes. BMC Genomics 14, 567. 10.1186/1471-2164-14-567. 23957789 PMC3751779

[84] Zhou X, Dou Q, Fan G, Zhang Q, Sanderford M, Kaya A, Johnson J, Karlsson EK, Tian X, Mikhalchenko A, (2020). Beaver and naked mole rat genomes reveal common paths to longevity. Cell Rep. 32, 107949. 10.1016/j.celrep.2020.107949. 32726638 PMC9385191

[85] Lin K, Dorman JB, Rodan A, and Kenyon C. (1997). daf-16: an HNF-3/forkhead family member that can function to double the life-span of Caenorhabditis elegans. Science 278, 1319–1322. 10.1126/science.278.5341.1319. 9360933

[86] Ogg S, Paradis S, Gottlieb S, Patterson GI, Lee L, Tissenbaum HA, and Ruvkun G. (1997). The Fork head transcription factor DAF-16 transduces insulin-like metabolic and longevity signals in C. elegans. Nature 389, 994–999. 10.1038/40194. 9353126

[87] Hwangbo DS, Gershman B, Tu MP, Palmer M, and Tatar M. (2004). Drosophila dFOXO controls lifespan and regulates insulin signalling in brain and fat body. Nature 429, 562–566. 10.1038/nature02549. 15175753

[88] Huang Y, Chen H, Yang P, Bai X, Shi Y, Vistro WA, Tarique I, Haseeb A, and Chen Q. (2019). Hepatic lipid droplet breakdown through lipolysis during hibernation in Chinese Soft-Shelled Turtle (Pelodiscus sinensis). Aging (Albany, NY) 11, 1990–2002. 10.18632/aging.101887. 30926766 PMC6503876

[89] Laplaud PM, Beaubatie L, Rall SC Jr., Luc G, and Saboureau M. (1988). Lipoprotein[a] is the major apoB-containing lipoprotein in the plasma of a hibernator, the hedgehog (Erinaceus europaeus). J. Lipid Res. 29, 1157–1170. 10.1016/S0022-2275(20)38452-2. 2972788

[90] Otis JP, Sahoo D, Drover VA, Yen CLE, and Carey HV (2011). Cholesterol and lipoprotein dynamics in a hibernating mammal. PLoS One 6, e29111. 10.1371/journal.pone.0029111. 22195001 PMC3240636

[91] Pedrelli M, Parini P, Kindberg J, Arnemo JM, Bjorkhem I, Aasa U, Westerståhl M, Walentinsson A, Pavanello C, Turri M, (2021). Vasculoprotective properties of plasma lipoproteins from brown bears (Ursus arctos). J. Lipid Res. 62, 100065. 10.1016/j.jlr.2021.100065. 33713671 PMC8131316

[92] Ren Y, Song S, Liu X, and Yang M. (2022). Phenotypic changes in the metabolic profile and adiponectin activity during seasonal fattening and hibernation in female Daurian ground squirrels (Spermophilus dauricus). Integr. Zool 17, 297–310. 10.1111/1749-4877.12504. 33190391

[93] Sommer F, Ståhlman M, Ilkayeva O, Arnemo JM, Kindberg J, Josefsson J, Newgard CB, Fröbert O, and Bäckhed F. (2016). The gut microbiota modulates energy metabolism in the hibernating brown bear Ursus arctos. Cell Rep. 14, 1655–1661. 10.1016/j.celrep.2016.01.026. 26854221

[94] Vella CA, Nelson OL, Jansen HT, Robbins CT, Jensen AE, Constantinescu S, Abbott MJ, and Turcotte LP (2020). Regulation of metabolism during hibernation in brown bears (Ursus arctos): involvement of cortisol, PGC-1alpha and AMPK in adipose tissue and skeletal muscle. Comp. Biochem. Physiol. A Mol. Integr. Physiol 240, 110591. 10.1016/j.cbpa.2019.110591. 31669707

[95] Batz ZA, and Armbruster PA (2018). Diapause-associated changes in the lipid and metabolite profiles of the Asian tiger mosquito, Aedes albopictus. J. Exp. Biol 221, jeb189480. 10.1242/jeb.189480. PMC6307873 30385483

[96] Pauli A, Valen E, Lin MF, Garber M, Vastenhouw NL, Levin JZ, Fan L, Sandelin A, Rinn JL, Regev A, and Schier AF (2012). Systematic identification of long noncoding RNAs expressed during zebrafish embryogenesis. Genome Res. 22, 577–591. 10.1101/gr.133009.111. 22110045 PMC3290793

[97] Church DM, Goodstadt L, Hillier LW, Zody MC, Goldstein S, She X, Bult CJ, Agarwala R, Cherry JL, DiCuccio M, (2009). Line-age-specific biology revealed by a finished genome assembly of the mouse. PLoS Biol. 7, e1000112. 10.1371/journal.pbio.1000112. 19468303 PMC2680341

[98] Ichikawa K, Tomioka S, Suzuki Y, Nakamura R, Doi K, Yoshimura J, Kumagai M, Inoue Y, Uchida Y, Irie N, (2017). Centromere evolution and CpG methylation during vertebrate speciation. Nat. Commun 8, 1833. 10.1038/s41467-017-01982-7. 29184138 PMC5705604

[99] Howe K, Clark MD, Torroja CF, Torrance J, Berthelot C, Muffato M, Collins JE, Humphray S, McLaren K, Matthews L, (2013). The zebrafish reference genome sequence and its relationship to the human genome. Nature 496, 498–503. 10.1038/nature12111. 23594743 PMC3703927

[100] Emms DM, and Kelly S. (2015). OrthoFinder: solving fundamental biases in whole genome comparisons dramatically improves orthogroup inference accuracy. Genome Biol. 16, 157. 10.1186/s13059-015-0721-2. 26243257 PMC4531804

[101] Altschul SF., Gish W., Miller W., Myers EW., and Lipman DJ. (1990). Basic local alignment search tool. J. Mol. Biol 215, 403–410. 10.1016/S0022-2836(05)80360-2. 2231712

[102] Love MI, Huber W, and Anders S. (2014). Moderated estimation of fold change and dispersion for RNA-seq data with DESeq2. Genome Biol. 15, 550. 10.1186/s13059-014-0550-8. 25516281 PMC4302049

[103] Andrew S. (2010). FastQC: a Quality Control Tool for High Throughput Sequence Data. https://www.bioinformatics.babraham.ac.uk/projects/fastqc/.

[104] Ewels P, Magnusson M, Lundin S, and Käller M. (2016). MultiQC: summarize analysis results for multiple tools and samples in a single report. Bioinformatics 32, 3047–3048. 10.1093/bioinformatics/btw354. 27312411 PMC5039924

[105] Dobin A, Davis CA, Schlesinger F, Drenkow J, Zaleski C, Jha S, Batut P, Chaisson M, and Gingeras TR (2013). STAR: ultrafast universal RNA-seq aligner. Bioinformatics 29, 15–21. 10.1093/bioinformatics/bts635. 23104886 PMC3530905

[106] Liao Y, Smyth GK, and Shi W. (2013). The Subread aligner: fast, accurate and scalable read mapping by seed-and-vote. Nucleic Acids Res. 41, e108. 10.1093/nar/gkt214. 23558742 PMC3664803

[107] Langmead B, and Salzberg SL (2012). Fast gapped-read alignment with Bowtie 2. Nat. Methods 9, 357–359. 10.1038/nmeth.1923. 22388286 PMC3322381

[108] Danecek P, Bonfield JK, Liddle J, Marshall J, Ohan V, Pollard MO, Whitwham A, Keane T, McCarthy SA, Davies RM, and Li H. (2021). Twelve years of SAMtools and BCFtools. GigaScience 10, giab008. 10.1093/gigascience/giab008. PMC7931819 33590861

[109] Ramírez F, Ryan DP, Grüning B, Bhardwaj V, Kilpert F, Richter AS, Heyne S, Dündar F, and Manke T. (2016). deepTools2: a next generation web server for deep-sequencing data analysis. Nucleic Acids Res. 44, W160–W165. 10.1093/nar/gkw257. 27079975 PMC4987876

[110] Zhang Y, Liu T, Meyer CA, Eeckhoute J, Johnson DS, Bernstein BE, Nusbaum C, Myers RM, Brown M, Li W, and Liu XS (2008). Model-based analysis of ChIP-Seq (MACS). Genome Biol. 9, R137. 10.1186/gb-2008-9-9-r137. 18798982 PMC2592715

[111] Harris RS (2007). Improved Pairwise Alignment of Genomic DNA. Ph.D. Thesis (Pennsylvania State University).

[112] Kuhn RM, Haussler D, and Kent WJ (2013). The UCSC genome browser and associated tools. Brief. Bioinform 14, 144–161. 10.1093/bib/bbs038. 22908213 PMC3603215

[113] Blanchette M, Kent WJ, Riemer C, Elnitski L, Smit AFA, Roskin KM, Baertsch R, Rosenbloom K, Clawson H, Green ED, (2004). Aligning multiple genomic sequences with the threaded blockset aligner. Genome Res. 14, 708–715. 10.1101/gr.1933104. 15060014 PMC383317

[114] Robinson JT, Thorvaldsdóttir H, Winckler W, Guttman M, Lander ES, Getz G, and Mesirov JP (2011). Integrative genomics viewer. Nat. Biotechnol 29, 24–26. 10.1038/nbt.1754. 21221095 PMC3346182

[115] Tang YH, and Masaaki L. (2016). Wenxuan ggfortify: unified Interface to Visualize Statistical Results of Popular R Packages. R J. 8/2, 474–485. 10.32614/RJ-2016-060.

[116] Stark R, and Brown G. (2011). DiffBind: differential binding analysis of ChIP-Seq peak data. http://bioconductor.org/packages/release/bioc/vignettes/DiffBind/inst/doc/DiffBind.pdf.

[117] Robinson MD, McCarthy DJ, and Smyth GK (2010). edgeR: a Bioconductor package for differential expression analysis of digital gene expression data. Bioinformatics 26, 139–140. 10.1093/bioinformatics/btp616. 19910308 PMC2796818

[118] Yu G, Wang LG, and He QY (2015). ChIPseeker: an R/Bioconductor package for ChIP peak annotation, comparison and visualization. Bioinformatics 31, 2382–2383. 10.1093/bioinformatics/btv145. 25765347

[119] Bailey TL, Boden M, Buske FA, Frith M, Grant CE, Clementi L, Ren J, Li WW, and Noble WS (2009). MEME SUITE: tools for motif discovery and searching. Nucleic Acids Res. 37, W202–W208. 10.1093/nar/gkp335. 19458158 PMC2703892

[120] Smit AFA, Hubley R, and Green P. (2013–2015). RepeatMasker Open-4.0. http://www.repeatmasker.org.

[121] Blokzijl F, Janssen R, van Boxtel R, and Cuppen E. (2018). Mutational-Patterns: comprehensive genome-wide analysis of mutational processes. Genome Med. 10, 33. 10.1186/s13073-018-0539-0. 29695279 PMC5922316

[122] Yang Z. (2007). PAML 4: phylogenetic analysis by maximum likelihood. Mol. Biol. Evol 24, 1586–1591. 10.1093/molbev/msm088. 17483113

[123] Lechner M, Findeiss S, Steiner L, Marz M, Stadler PF, and Prohaska SJ (2011). Proteinortho: detection of (co-)orthologs in large-scale analysis. BMC Bioinformatics 12, 124. 10.1186/1471-2105-12-124. 21526987 PMC3114741

[124] Löytynoja A. (2014). Phylogeny-aware alignment with PRANK. Methods Mol. Biol 1079, 155–170. 10.1007/978-1-62703-646-7_10. 24170401

[125] Sela I, Ashkenazy H, Katoh K, and Pupko T. (2015). GUIDANCE2: accurate detection of unreliable alignment regions accounting for the uncertainty of multiple parameters. Nucleic Acids Res. 43, W7–W14. 10.1093/nar/gkv318. 25883146 PMC4489236

[126] Falcon S, and Gentleman R. (2007). Using GOstats to test gene lists for GO term association. Bioinformatics 23, 257–258. 10.1093/bioinformatics/btl567. 17098774

[127] Labun K, Montague TG, Krause M, Torres Cleuren YN, Tjeldnes H, and Valen E. (2019). CHOPCHOP v3: expanding the CRISPR web toolbox beyond genome editing. Nucleic Acids Res. 47, W171–W174. 10.1093/nar/gkz365. 31106371 PMC6602426

[128] Podrabsky JE (1999). Husbandry of the annual killifish Austrofundulus limnaeus with special emphasis on the collection and rearing of embryos. Environmental Biology of Fishes 54, 421–431. 10.1023/A:1007598320759.

[129] Polačik M, Blăzek R, and Reichard M. (2016). Laboratory breeding of the short-lived annual killifish Nothobranchius furzeri. Nat. Protoc 11, 1396–1413. 10.1038/nprot.2016.080. 27388556

[130] Dodzian J, Kean S, Seidel J, and Valenzano DR (2018). A Protocol for Laboratory Housing of Turquoise killifish (Nothobranchius furzeri). J. Vis. Exp 10.3791/57073. PMC5933446 29708537

[131] Nath RD, Bedbrook CN, Nagvekar R, and Brunet A. (2023). Husbandry of the African Turquoise killifish Nothobranchius furzeri. Cold Spring Harb. Protoc. 2023, pdb.prot107738. 10.1101/pdb.prot107738. 36863854

[132] Podrabsky JE, Riggs CL, Romney AL, Woll SC, Wagner JT, Culpepper KM, and Cleaver TG (2017). Embryonic development of the annual killifish Austrofundulus limnaeus: an emerging model for ecological and evolutionary developmental biology research and instruction. Dev. Dyn 246, 779–801. 10.1002/dvdy.24513. 28481428

[133] Podrabsky JE, Garrett IDF, and Kohl ZF (2010). Alternative developmental pathways associated with diapause regulated by temperature and maternal influences in embryos of the annual killifish Austrofundulus limnaeus. J. Exp. Biol 213, 3280–3288. 10.1242/jeb.045906. 20833920 PMC2936969

[134] Podrabsky JE, and Hand SC (1999). The bioenergetics of embryonic diapause in an annual killifish, austrofundulus limnaeus. J. Exp. Biol 202, 2567–2580. 10.1242/jeb.202.19.2567. 10482717

[135] Doganli C, Sandoval M, Thomas S, and Hart D. (2017). Assay for transposase-accessible chromatin with high-throughput sequencing (ATAC-Seq) protocol for zebrafish embryos. Methods Mol. Biol 1507, 59–66. 10.1007/978-1-4939-6518-2_5. 27832532

[136] Harel I, Valenzano DR, and Brunet A. (2016). Efficient genome engineering approaches for the short-lived African turquoise killifish. Nat. Protoc 11, 2010–2028. 10.1038/nprot.2016.103. 27658015

[137] Reeves GA, Singh PP, and Brunet A. (2024). Chromatin Accessibility Profiling and Data Analysis Using ATAC-seq in Nothobranchius furzeri. Cold Spring Harb. Protoc. 2024, pdb.prot107747. 10.1101/pdb.prot107747. 37100469

[138] Contrepois K, Mahmoudi S, Ubhi BK, Papsdorf K, Hornburg D, Brunet A, and Snyder M. (2018). Cross-platform comparison of untargeted and targeted lipidomics approaches on aging mouse plasma. Sci. Rep 8, 17747. 10.1038/s41598-018-35807-4. 30532037 PMC6288111

[139] Yang AC, Stevens MY, Chen MB, Lee DP, Stähli D, Gate D, Contrepois K, Chen W, Iram T, Zhang L, (2020). Physiological blood-brain transport is impaired with age by a shift in transcytosis. Nature 583, 425–430. 10.1038/s41586-020-2453-z. 32612231 PMC8331074

[140] Schindelin J, Arganda-Carreras I, Frise E, Kaynig V, Longair M, Pietzsch T, Preibisch S, Rueden C, Saalfeld S, Schmid B, (2012). Fiji: an open-source platform for biological-image analysis. Nat. Methods 9, 676–682. 10.1038/nmeth.2019. 22743772 PMC3855844

[141] Yates AD, Achuthan P, Akanni W, Allen J, Allen J, Alvarez-Jarreta J, Amode MR, Armean IM, Azov AG, Bennett R, (2020). Ensembl 2020. Nucleic Acids Res. 48, D682–D688. 10.1093/nar/gkz966. 31691826 PMC7145704

[142] Liao Y, Smyth GK, and Shi W. (2014). featureCounts: an efficient general purpose program for assigning sequence reads to genomic features. Bioinformatics 30, 923–930. 10.1093/bioinformatics/btt656. 24227677

[143] Liu T. (2014). Use model-based analysis of ChIP-Seq (MACS) to analyze short reads generated by sequencing protein-DNA interactions in embryonic stem cells. Methods Mol. Biol 1150, 81–95. 10.1007/978-1-4939-0512-6_4. 24743991

[144] Margulies EH, Vinson JP, NISC; Comparative; Sequencing Program, Miller W, Jaffe DB, Lindblad-Toh K, Chang JL, Green ED, Lander ES, Mullikin JC, and Clamp M. (2005). An initial strategy for the systematic identification of functional elements in the human genome by low-redundancy comparative sequencing. Proc. Natl. Acad. Sci. USA 102, 4795–4800. 10.1073/pnas.0409882102. 15778292 PMC555705

[145] Miller W, Rosenbloom K, Hardison RC, Hou M, Taylor J, Raney B, Burhans R, King DC, Baertsch R, Blankenberg D, (2007). 28-way vertebrate alignment and conservation track in the UCSC Genome Browser. Genome Res. 17, 1797–1808. 10.1101/gr.6761107. 17984227 PMC2099589

[146] Ross-Innes CS, Stark R, Teschendorff AE, Holmes KA, Ali HR, Dunning MJ, Brown GD, Gojis O, Ellis IO, Green AR, (2012). Differential oestrogen receptor binding is associated with clinical outcome in breast cancer. Nature 481, 389–393. 10.1038/nature10730. 22217937 PMC3272464

[147] Gupta S, Stamatoyannopoulos JA, Bailey TL, and Noble WS (2007). Quantifying similarity between motifs. Genome Biol. 8, R24. 10.1186/gb-2007-8-2-r24. 17324271 PMC1852410

[148] Gao B, Shen D, Xue S, Chen C, Cui H, and Song C. (2016). The contribution of transposable elements to size variations between four teleost genomes. Mobile DNA 7, 4. 10.1186/s13100-016-0059-7. 26862351 PMC4746887

[149] Sotero-Caio CG, Platt RN 2nd, Suh A, and Ray DA (2017). Evolution and diversity of transposable elements in vertebrate genomes. Genome Biol. Evol 9, 161–177. 10.1093/gbe/evw264. 28158585 PMC5381603

[150] Jialin L, V.R.R., Khoueiry P, Reddington JP, Girardot C, Furlong EEM, and Robinson-Rechavi M. (2021). The hourglass model of evolutionary conservation during embryogenesis extends to developmental enhancers with signatures of positive selection. Preprint at bioRxiv. 10.1101/2020.11.02.364505. PMC8415374 34266978

[151] Ignatiadis N, Klaus B, Zaugg JB, and Huber W. (2016). Data-driven hypothesis weighting increases detection power in genome-scale multiple testing. Nat. Methods 13, 577–580. 10.1038/nmeth.3885. 27240256 PMC4930141

[152] Wu T, Hu E, Xu S, Chen M, Guo P, Dai Z, Feng T, Zhou L, Tang W, Zhan L, (2021). clusterProfiler 4.0: A universal enrichment tool for interpreting omics data. Innovation (Camb) 2, 100141. 10.1016/j.xinn.2021.100141. 34557778 PMC8454663

