# Supplementary material for: Evolution of diapause in the African turquoise killifish by remodeling the ancient gene regulatory landscape
Source: Cell. Author manuscript; Available in PMC 2025 Apr 4. (PMC11970524; doi:10.1016/j.cell.2024.04.048)
Supplement: Supplemental [file NIHMS2059281-supplement-Supplemental.pdf]

CellPress
OPEN ACCESS

STAR★METHODS

KEY RESOURCES TABLE

REAGENT or RESOURCE	SOURCE	IDENTIFIER
Chemicals, Peptides, and Recombinant Proteins		
EquiSPLASH LIPIDOMIX	Avanti Polar Lipids	Cat. No.330731
d17-Oleic acid	Cayman chemicals	Cat. No.9000432
Alt-R™ S.p. Cas9 Nuclease	Integrated DNA Technologies (IDT)	Cat. No. 1081058
BODIPY™ 493/503	Invitrogen	Cat. No. D3992
Otohime C1 Fish Pellets	Reed Mariculture	N/A
Otohime Ep1 Fish Pellets	Reed Mariculture	N/A
Ringers Solution	Sigma-Aldrich	Cat. No. 96724
Methylene Blue	Sigma-Aldrich	Cat. No. 319112
EZ-lysis buffer	Sigma-Aldrich	Cat. No.3408
Methyl tert-butyl ether (MTBE)	Sigma-Aldrich	Cat. No. 316466
Methanol	Sigma-Aldrich	Cat. No. MX0482
Toluene	Sigma-Aldrich	Cat. No. 244511
Phosphate Buffered Saline (PBS)	ThermoFisher	Cat. No. AM9624
Trypan blue stain	ThermoFisher	Cat. No.15250061
Nuclease-free water	ThermoFisher	Cat. No.AM9916
2X DreamTaq PCR Master Mix	ThermoFisher	Cat. No. K1081
RNase Zap™ RNase Decontamination Solution	ThermoFisher	Cat. No. AM9780
Critical Commercial Assays		
Agilent's High Sensitivity DNA Kit	Agilent	Cat. No. 5067-4626
Nextera XT DNA Library Prep Kit	Illumina	Cat. No. FC-131-1096
Tn5 Transposition DNA Buffer and Enzyme kit	Illumina	Cat. No. 20034197
High Yield Short Read Nextseq Kit	Illumina	Cat. No. PE-410-1001
BCA Protein Assay Kit	Pierce	Cat. No.23225
Mini-elute kit	Qiagen	Cat. No. 28206
SMART-Seq® v4 Ultra® Low Input RNA Kit	Takara	Cat. No. 634890
Deposited Data		
Raw and analyzed data	This paper	GSE185817
Lipidomics data	This paper	ST001898
Additional African turquoise killifish RNA-seq data	Hu et al.[6]	PRJNA503701
African turquoise killifish reference genome: Nfu_20140520	Reichwald et al.[8]	https://www.ncbi.nlm.nih.gov/datasets/genome/GCF_001465895.1/
South American killifish diapause RNA-seq data	Wagner et al.[9]	PRJNA272154
South American killifish reference genome: Austrofundulus_limnaeus-1.0	Wagner et al.[9]	https://0-www-ncbi-nlm-nih-gov.brum.beds.ac.uk/datasets/genome/GCF_001266775.1/
Mouse RNA-seq data	Hussien et al.[22]	GSE143494
Medaka ATAC-seq data	Marlétaz et al.[24]	GSE106428
Zebrafish ATAC-seq data	Marlétaz et al.[24]	GSE106428
Zebrafish late development RNA-seq data	Marlétaz et al.[24]	GSE106430
Medaka developmental RNA-seq data	Marlétaz et al.[24]	GSE106430

(*Continued on next page*)

Continued

REAGENT or RESOURCE	SOURCE	IDENTIFIER
Lyretail killifish reference genome: MPIBA_Aaus_1.0	Cui et al.[29]	https://www.ncbi.nlm.nih.gov/nuccore/SSNS00000000.1/
South American killifish developmental RNA-seq data	Romney et al.[73]	PRJNA272154
Zebrafish early developmental RNA-seq data	Pauli et al.[96]	GSE32898
Mouse reference genome: GCF_000001635.20 (mm10)	Church et al.[97]	https://www.ncbi.nlm.nih.gov/datasets/genome/GCF_000002035.6/
Medaka reference genome: ASM223467v1	Ichikawa et al.[98]	https://www.ncbi.nlm.nih.gov/datasets/genome/GCF_002234675.1/
Zebrafish reference genome: GRCz11	Howe et al.[99]	https://www.ncbi.nlm.nih.gov/datasets/genome/GCF_002234675.1/
Experimental Models: Organisms/Strains		
Nothobranchius furzeri	Brunet Lab	GRZ strain
Brine Shrimp Eggs	Brine Shrimp Direct	Cat. No. 454GR
Austrofundulus limnaeus	Podrabsky Lab	Quisiro strain
Aphyosemion australe	Wetspot Tropical Fish	Gold strain
Aphyosemion striatum	Wetspot Tropical Fish	Aquarium strain
Oligonucleotides		
Primers for *REST*, *FOXO3a*, *FOXO3b*, *PPARAa*, *PPARAb*, and *PPARG*, see Table S5B	This paper	N/A
sgRNA sequences for *REST*, *FOXO3a*, *FOXO3b*, *PPARAa*, *PPARAb*, and *PPARG*, see Table S5B	This paper	N/A
Software and Algorithms		
Original code generated for the study.	This paper	https://github.com/SinghLabUCSF/Diapause-multiomics
Picard Tools v2.22.1	Broad Institute	https://broadinstitute.github.io/picard/
SnapGene v7.0	Dotmatic	https://www.snapgene.com/
TrimGalore v0.4.1	Felix Krueger	https://www.bioinformatics.babraham.ac.uk/projects/trimgalore/
Fiji v2.0.0-rc-68/1.52h	FijiTeam	https://fiji.sc/
Ingenuity Pathway Analysis (IPA)	QIAGEN	https://digitalinsights.qiagen.com/products-overview/discovery-insights-portfolio/analysis-and-visualization/Qiagen-ipa/
R v3.6.2	R core team	https://www.r-project.org/
ICE Analysis v.1.0	Synthego	https://ice.synthego.com/#/
LipidSearch v4.2.21	ThermoFisher	https://www.thermofisher.com/us/en/home/industrial/mass-spectrometry/liquid-chromatography-mass-spectrometry-lc-ms/lc-ms-software/multi-omics-data-analysis/lipid-search-software.html
Zen Blue v3.4.0	Zeiss	https://www.zeiss.com/microscopy/en/products/software/zeiss-zen.html#zenversions
HOMER v4.10	Heinz et al.[27]	http://homer.ucsd.edu/homer/
OrthoFinder v2.5.4	Emms and Kelly[100]	https://github.com/davidemms/OrthoFinder
BLASTp v2.7.1+	Altschul et al.[101]	https://blast.ncbi.nlm.nih.gov/Blast.cgi?PAGE=Proteins

(*Continued on next page*)

Continued

REAGENT or RESOURCE	SOURCE	IDENTIFIER
DEseq2 v1.30.1	Love et al.[102]	https://bioconductor.org/packages/release/bioc/html/DESeq2.html
FastQC v0.11.9	Andrew[103]	http://www.bioinformatics.babraham.ac.uk/projects/fastqc
MultiQC v1.8	Ewels et al.[104]	https://multiqc.info
STAR v2.7.1a	Dobin et al.[105]	https://github.com/alexdobin/STAR
Subread v2.0.1	Liao et al.[106]	https://sourceforge.net/projects/subread/
BowTie2 v2.2.5	Langmead and Salzberg[107]	http://bowtie-bio.sourceforge.net/bowtie2/index.shtml
Samtools v1.5	Danecek et al.[108]	http://www.htslib.org/
deepTools v3.2.1	Ramirez et al.[109]	https://deeptools.readthedocs.io/en/develop/
MACS2	Zhang et al.[110]	https://pypi.org/project/MACS2/#description
LASTz v1.04.00	Harris[111]	https://www.bx.psu.edu/~rsharris/lastz/
UCSC genome utilities v15.6.0	Kuhn et al.[112]	http://hgdownload.soe.ucsc.edu/admin/exe/
Multic/TBA v10	Blanchette et al.[113]	https://www.bx.psu.edu/miller_lab/
Integrative Genomics Viewer (IGV) v2.4.18	Robinson et al.[114]	https://software.broadinstitute.org/software/igv/
ggfortify v0.4.11	Tang and Masaaki[115]	https://cran.r-project.org/web/packages/ggfortify/index.html
DiffBind v2.16.2	Stark and Brown[116]	https://hbctraining.github.io/Intro-to-ChIPseq/lessons/08_diffbind_differential_peaks.html
EdgeR v4.0.16	Robinson et al.[117]	https://bioconductor.org/packages/release/bioc/html/edgeR.html
CHIPseeker v1.28.3	Yu et al.[118]	https://bioconductor.org/packages/release/bioc/html/ChIPseeker.html
MEME suit v5.3.0	Bailey et al.[119]	https://meme-suite.org/meme/doc/download.html
RepeatMasker v4.0	Smit et al.[120]	https://www.repeatmasker.org/
Mutational Patterns Package v3.2.0	Blokzjl et al.[121]	https://bioconductor.org/packages/release/bioc/html/MutationalPatterns.html
PAML v4.8	Yang[122]	http://abacus.gene.ucl.ac.uk/software/paml.html
Proteinortho v5.11	Lechner et al.[123]	https://www.bioinf.uni-leipzig.de/Software/proteinortho/
PRANK v.140603	Loytynoja[124]	https://ariloytynoja.github.io/prank-msa/
GUIDANCE v2.0	Sela et al.[125]	http://guidance.tau.ac.il/source.php
GOstats package v2.56.0	Falcon and Gentleman[126]	https://bioconductor.org/packages/release/bioc/html/GOstats.html
CHOPCHOP v3.0.0	Labun et al.[127]	https://chopchop.cbu.uib.no/
Other		
Mini-douncers	DWK (Kimble)	Cat. No.885300-0000
Biological-grade tweezers	Electron Microscopy Sciences	Cat. No. 72700-D
1.5ml Eppendorf™ DNA loBind microcentrifuge tubes	Eppendorf	Cat. No. 13-698-791
PYREX™ Spot Plate	Fisher Scientific	Cat. No. 13-748B
Extra Coarse Glass Beads (30/40 Mesh, 425-560micron size)	Kramer Industries Inc.	N/A
FastPrep® -24 homogenizer	MB Biomedicals	Cat. No. 116004500

(*Continued on next page*)

Continued		
REAGENT or RESOURCE	SOURCE	IDENTIFIER
Zirconia/Silicon 0.5mm glass beads	Research Products International Corp	Cat. No. 9834
Accucore C30 column 2.1x150mm, 2.6μm	ThermoFisher	Cat. No. TFS-27826-152130
LSM 900 Airyscan SR Confocal Microscope	Zeiss	N/A

RESOURCE AVAILABILITY

Lead contact

- Further information and requests for resources and reagents should be directed to and will be fulfilled by the lead contact, Anne Brunet (abrunet1@stanford.edu).

Materials availability

- This study did not generate new unique reagents.

Data and code availability

- All the RNA-seq and ATAC-seq data generated in this study have been deposited to NCBI-GEO (accession # GSE185817) and are publicly available as of the date of publication. Accession numbers are listed in the key resources table. All the lipidomics data generated in this study have been deposited to the Metabolomic Workbench (Study ID ST001898) and are publicly available as of the date of publication. Accession numbers are listed in the key resources table. This paper analyzes existing, publicly available data. These accession numbers for the datasets are listed in the key resources table.
- All original code has been deposited at Github and is publicly available as of the date of publication. DOIs are listed in the key resources table.
- Any additional information required to reanalyze the data reported in this paper is available from the lead contact upon request.

EXPERIMENTAL MODEL AND STUDY PARTICIPANT DETAILS

Killifish species and husbandry

The killifish and other outgroup species used in this study are listed in Table S1A. All the killifish species used for data generation were housed in the Stanford Research Animal Facility II under the approved protocol (protocol #APLAC-13645). Animals were housed in automated circulating water system with pH maintained at 6-7.5 and conductivity maintained between 3500 and 4500μS/cm with a 10% system water exchange every day by reverse osmosis treated water. Adult fish were manually fed Otohime fish diet (Reed Mariculture, Otohime C1 [Ep1 for the South American killifish]) twice a day during weekdays and once a day during weekends.

Newly hatched fries for all species were kept in 0.8-liter fry tanks at a density of 4-5 fries for first two weeks and then individually housed for next two weeks. Fries were fed newly hatched brine shrimps (Brine Shrimp Direct, 454GR) twice a day during weekdays, and once a day during weekends. Animals were sexed at 4 weeks of age and transferred to 2.8-liter tanks. For African turquoise killifish and South American killifish (with diapause), adult males and females were individually housed except for breeding. Red-striped killifish, and lyretail killifish adults were kept in pairs with one male and one female animal in each tank.

For breeding, African turquoise killifish and South American killifish (with diapause) males and females were transferred to breeding tanks for a period of ~5 hours. Breeding tanks had sand trays at the bottom for the African turquoise killifish and trays with extra coarse grade glass beads (30/40 Mesh, 425-560micron size, Kramer Industries Inc. USA) for the South American killifish as per the established protocols.[128–131] After ~5 hours, sand or glass beads were filtered using a sieve to collect embryos. For the red-striped killifish and the lyretail killifish (without diapause), spawning mops constructed using green yarn were floated from the lid. The yarns were checked every day for embryos, and the embryos were carefully hand-picked.

We used young animals (1-3 months of age) for breeding and embryo collection. For each species, collected embryos were washed multiple times and live embryos were placed in Ringer's solution (Sigma-Aldrich, 96724) with 0.01% methylene blue at 26°C. Embryos were checked under a stereoscope every day and any dead embryos were removed.

Staging of killifish embryos

Synchronized killifish embryos for African turquoise and South American killifish were collected within a tight (~5 hour) breeding window. Most collected embryos were at the 1-2 cell stage upon collection. We monitored embryos every day post-collection to observe the visual markers of diapause and development as previously described.[6] Briefly, we used Kupffer's vesicle (KV), which is a transient embryonic organ present from early to middle somitogenesis as a marker to stage embryos that are about to reach diapause. KV-positive embryos reach the end of somitogenesis in 1-2 days and the loss of KV roughly coincides with the onset of heartbeat in

killifish, followed by either diapause or continue development.[6,132] We counted the number of somites in KV-positive embryos and designated KV-positive embryos at 15-25 somites as our *"pre-diapause (Pre-Dia) stage".* Embryo morphology for all the killifish species was similar at this stage. This mid-somitogenesis time point also coincides with the vertebrate phylotypic period (the period of the most conserved gene expression pattern during vertebrate development) with available gene expression and chromatin accessibility data from multiple other fish species.[24]

In killifish species with diapause, young mothers have most of their embryos develop directly, whereas more mature mothers (even before middle age) have an increased frequency of embryos in diapause.[6,133] This feature allows us to collect *pre-diapause* embryos, even though there are no known markers, as of yet, to determine if embryos at an earlier stage are destined to diapause. Therefore, for the African turquoise and South American killifish, we collected *pre-diapause (pre-Dia)* embryos from the very first breeding session (first clutch) from young mothers and fathers (age 4–5 weeks) with most embryos expected to skip diapause and continue developing which ensured that we get development bound embryos at *pre-diapause (pre-Dia)* stage.

Among the first visual markers of diapause is the slowing of the rate of heartbeat after its onset.[6,134] Therefore, we next monitored the onset of heartbeat, and stage diapause embryos at *6 days (Dia 6d)* and *1 month of diapause* (*Dia 1m*) as exhibiting a continuously decreasing heartbeat rate since diapause onset (<45 beat-per-minute (BPM)) as described in Hu et al.[6] For embryos in 1 month diapause (*Dia 1m*), we additionally made sure that the heartbeat was less than 1 beat per minute by monitoring them under a stereoscope to verify that they were not prematurely exiting the diapause state. For embryos in development, embryos that had an increase in heartbeat rate 1 day after heartbeat onset (>45 BPM), but before the visual pigmentation in eyes was developed (i.e. before pharyngula stage) were designated as *developing embryos (Dev)*.[6] All the diapause and development stages stage are identical to our previous study,[6] except *Pre-Dia* stage which is roughly a day before the onset of heartbeat. For killifish species without diapause (red-striped and lyretail killifish), we followed the same staging procedure described above to collect embryos at *pre-diapause (pre-Dia) stage* (development embryos with 15-25 somites; ~1 day before the onset of heartbeat). Because there is no diapause in these killifish, development embryos were taken as 1 day after the onset of heartbeat to match to the *Dev* stage in the African turquoise killifish.

Killifish embryo collection

For each stage in each species, roughly 8-30 embryos were carefully dissected in ice-cold PBS using biological-grade tweezers (Electron Microscopy Sciences, 72700-D) to carefully remove the chorion, the enveloping layer, and the yolk without damaging the embryo body. Freshly dissected embryos were then quickly rinsed with ice-cold PBS, and all the PBS was carefully removed. Embryo bodies were then snap-frozen in liquid nitrogen and stored at -80°C. We used 8-10 snap-frozen embryos for RNA-seq and ATAC-seq and 25-30 embryos for lipidomics (see below). The details of all samples and stages used are in Table S1B.

METHOD DETAILS

RNA-seq preparation for killifish species

To profile gene expression at pre-diapause stages in the African turquoise, red-striped and lyretail killifish, we constructed RNA-seq libraries (Table S1B, GSE185815, https://www.ncbi.nlm.nih.gov/geo/query/acc.cgi?acc=GSE185815). Snap frozen embryos at -80°C were thawed on ice for 1 minute and washed with 200μl ice-cold PBS. The embryos were then dissociated and homogenized with ~25 Zirconia/Silicon 0.5mm glass beads (RPI, Research Products International Corp, 9834) using FastPrep® -24 homogenizer (MB Biomedicals, 116004500) for 20 seconds, followed by centrifugation (17000g for 3 minutes). After centrifugation, 10.5μl of the supernatant was used as input to the SMART-Seq® v4 Ultra® Low Input RNA Kit (Takara, 634890) for the cDNA synthesis followed by amplification with 12 cDNA amplification cycles. Amplified cDNA was validated with Agilent 2100 Bioanalyzer using Agilent's High Sensitivity DNA Kit (Agilent, Cat. No. 5067-4626). The DNA libraries were then generated using the Nextera XT DNA Library Prep Kit (Illumina, FC-131-1096). Library quality and concentration were assessed by the Agilent 2100 Bioanalyzer and Agilent's High Sensitivity DNA kit (Agilent Technologies, Cat. No. 5067-4626), followed by high throughput sequencing on Illumina HiSeq platform with 2 x 150bp paired end reads.

In addition, we also used available African turquoise killifish,[6] South American killifish,[9,73] medaka,[24] zebrafish[24,96] and mouse[22] embryo RNA-seq data for our analysis (Table S1B), and processed them using the same pipeline described below. For medaka and zebrafish, we used mid-somitogenesis stages for our analysis that are expected to be the closest across vertebrates[24] (Table S1B).

ATAC-seq library preparation

To identify diapause-specific regulatory regions in the genome of African turquoise killifish and how these have evolved, we performed the Assay of Transposase Accessible Chromatin followed by high throughput sequencing (ATAC-seq)[23,135] in the embryos of multiple species. ATAC-seq is an unbiased and sensitive assay of genome-wide accessible chromatin landscape that requires very low input material. We performed ATAC-seq on embryos collected from five different killifish species with and without diapause, and at different stages of development and diapause (Table S1B). To generate nuclei-suspension for ATAC-seq libraries, snap frozen embryo samples (~10 embryos per sample) were thawed for 1 minute and resuspended at 4°C in 200μl EZ-lysis buffer (Sigma Aldritch No. 3408). Samples were then transferred to 250μl mini-douncers (DWK (Kimble) 885300-0000) and dounced 25 times with pestle A

and B respectively. After a 2 minute incubation following douncing, samples were spun at 500g for 5 minutes to precipitate nuclei, and the EZ-lysis supernatant was removed. Nuclei were then resuspended in 250μl PBS (ThermoFisher No. AM9624) and an aliquot of 5μl of nuclei was incubated with 5μl of 0.4% trypan blue stain (ThermoFisher No. 15250061) for counting the total intact nuclei counts.

Samples of ~25,000 nuclei were then suspended in a Tn5 transposition mix (65μl of tagmentation DNA buffer (Illumina No. 20034197), 63μl of nuclease-free water, and 2.5μl of tagmentation DNA enzyme I (e.g Tn5 transposase) (Illumina No. 20034197) for 20 minutes at 37°C. Following incubation, the mix was purified using the Qiagen mini-elute kit (Qiagen No. 28206) to isolate tagmented DNA. PCR amplification and subsequent qPCR monitoring was performed as described in the original ATAC-seq protocol (~14-18 cycles of PCR).[23] Amplified DNA from the PCR reaction was purified using the Qiagen mini-elute kit (Qiagen No. 28206), as recommended by the manufacturer. Samples were subsequently pooled and sequenced using next-generation short-read sequencing on an Illumina Nextseq 550 (Illumina No. PE-410-1001) with 75bp paired-end reads.

Generation of F0 knockout embryos

African turquoise killifish embryos were collected from breeding tanks, each with 1 young GRZ male (1.5-3 months) and 3 young GRZ females (1.5-3 months), co-housed for only 3-4 hours to ensure the collection of embryos at the single-cell stage. The relatively young age for females was chosen so that there would be ~50%-85% of embryos destined for diapause and ~15%-50% of embryos destined for direct development.[6] Once collected, viable embryos were washed with ~1ml of embryo solution (Ringer's solution with 0.01% methylene blue). Once cleaned, embryos were mounted in a 2% agarose gel mount with ~1mm width and ~1mm deep grooves to hold embryos in place for injection.[136] While in the gel mount, embryos were split into 3 groups per embryo collection: i) Wildtype (non-injected embryos); ii) Scramble (embryos injected with scrambled sgRNAs and Cas9). For these embryos, ~0.01μl Alt-R™ S.p. Cas9 Nuclease (22μM, IDT, Cat. No. 1081058) and three scrambled sgRNAs (~7.3μM each, 22μM total, IDT) were injected (Table S5B); or iii) F0 knockout embryos (injected with target transcription factor sgRNAs and Cas9). For these F0 knockout embryos, ~0.01μl of Cas9 (22μM) and three sgRNAs (~7.3μM each, 22μM total) targeting the first two exons of the candidate of interest (*REST*, *FOXO3a*, *FOXO3b*, *PPARAa*, *PPARAb*, or *PPARG*) were injected. For prioritization of candidates, see sections below: "TF knockout selection" and "sgRNA design". Embryos from each embryo collection were divided roughly equally among these 3 groups. Injected embryos from these experiments (F0 generation) were monitored for the following 10-14 days until the desired stage of diapause/development was reached. During this period, both embryo survival and diapause entry rates were tracked (Table S5D). Embryo death was monitored on a daily basis. Entry in diapause was assessed by using the method previously described in,[6,132] which involves heartbeat onset. Heartbeat onset and rate was assessed daily on a dissection scope manually. It represents a very robust method to distinguish embryos in diapause and embryos in development.[6,132] One day after heartbeat onset was used to determine entry into diapause (heartbeat < ~20 beats per minute) or direct development (heartbeat > ~85 beats per minute). Embryos in development were processed on 1 day post heart beat onset. Embryos in diapause were incubated for an additional five days to reach the '6-days in diapause' timepoint.

Knockout RNA-seq library generation

To generate single embryo RNA-seq libraries, individual injected (F0) embryos were dissected at the desired stages: development (1 day post-heartbeat onset, high heartbeat) and diapause (6-days post-heartbeat onset, low heartbeat). For dissection, the dissection scope and tools were treated with RNase Zap™ to prevent contamination during sample preparation. Dissection was carried out in 1x PBS chilled to 4°C, as previously described.[137] Briefly, forceps were used to remove both the chorion and embryonic membrane, removing the yolk, and taking only the embryo body for collection into one 1.5ml Eppendorff™ DNA loBind microcentrifuge tube. Each embryo was placed in one single tube and excess dissection supernatant (4°C 1x PBS) was removed. Each injected (F0) embryo represents an independent sample and it is entirely used for RNA-seq and knockout validation. The dry single embryos were then centrifuged (14,000g for 1 minute at 4°C) and resuspended in 15ml of 4°C 1x PBS and were subsequently dissociated and homogenized with ~25 Zirconia/Silicon 0.5mm glass beads (RPI, Research Products International Corp, 9834) using FastPrep® -24 homogenizer (MB Biomedicals, 116004500) for 1 minute, followed by centrifugation (10,000g for 1 minute at 4°C). After centrifugation, 10μl of the supernatant was used as input to the SMART-Seq® v4 Ultra® Low Input RNA kit (Takara, 634890) for cDNA synthesis followed by amplification with 12 cDNA amplification (PCR) cycles (1min at 95°C, 12 cycles of [10sec at 98°C, 30sec at 65°C, 3min at 68°C], 10min at 72°C, and held at 4°C). Amplified cDNA was validated with Agilent 2100 Bioanalyzer using Agilent's High Sensitivity DNA Kit (Agilent, Cat. No. 5067-4626). The DNA libraries were then generated using 1ng of cDNA material from each prep, using the Nextera XT DNA Library Prep kit (Illumina, FC-131-1096), following the manufacturer's instructions. Library quality and concentration were assessed by the Agilent 2100 Bioanalyzer and Agilent's High Sensitivity DNA kit (Agilent Technologies, Cat. No. 5067-4626), followed by high throughput sequencing on Illumina Nova-Seq 6000 platform with 2 x 150bp paired-end reads.

CRISPR/Cas9 knockout validation

We used several independent methods to validate editing events using our single embryo lysates. After the glass bead homogenizing and centrifugation steps described above, we also used ~0.5–5μl of supernatant for genotyping as the embryo lysates should also contain genomic DNA. This supernatant was added to 20μl of PCR mix (10μl 2X DreamTaq PCR master mix, 8μl of water, and 1μl of custom forward and reverse primers for each gene of interest; Table S5B) and amplified for 40 cycles (2min – 95°C, 40 cycles

of: 30sec – 95°C, 30sec – 59°C, 1min – 75°C). PCR products were submitted to Molecular Cloning Laboratories (MCLab) for PCR-cleanup (Cat. No. SEQ-CU) and sent for Easy Format™ Reactions for Sanger sequencing (Cat. No. SEQ-EZ).

Sanger sequencing chromatograms were visualized using SnapGene v7.0 to assess if they had biphasic peaks at sgRNA sites – a characteristic of the presence of different bases at one location. We also aligned each chromatogram to the reference gene in the African turquoise killifish genome to inspect the nature of the mutations (Nfu_20140520). Finally, the sequences were assessed for potential knockouts using the Synthego ICE analysis platform v1.0. The wildtype or control sgRNA-injected sample sequences with the highest quality score and best alignment to the locus of interest were used as the background, ensuring high quality ICE-scores for all sequences. The Synthego ICE platformed aligned all 122 samples' Sanger-sequencing products (56 Wildtype/Scramble and 66 knockouts). From these samples, the mutant libraries had a median predicted knockout score, based on ICE alignment of chromatograms, of 75%, ranging from 45% (*PPARAb* knockout) to 94% (*FOXO3b* knockout) (Figures S6E and S6F; Table S5B). We used the combination of these metrics and validation of the RNA-seq reads to inform our downstream analysis (see Criteria below).

Untargeted lipidomics by LC-MS

Lipidomics experiments were performed using ~30 embryos for each stage of diapause and development from African turquoise and red-striped killifish (3-4 replicates for each stage) (Figure 7A) as previously described.[138,139] We specifically chose the pre-diapause timepoint (day of heartbeat onset) as it is a well-conserved window of development across many species. This allows for comparison between species at this timepoint.

Lipids were extracted in a randomized order via biphasic separation with cold methyl tert-butyl ether (MTBE), methanol and water. Briefly, 260µl of methanol and 40µl of water were added to the embryos and vortexed for 20 seconds. A lipid internal standard mixture was spiked in each sample (EquiSPLASH LIPIDOMIX, Avanti Polar Lipids (cat #: 330731), and d17-Oleic acid, Cayman chemicals (cat #: 9000432) to control for extraction efficiency, evaluate LC-MS performance and estimate concentrations of individual lipids. Samples were diluted with 1,000µl of MTBE, vortexed for 10 seconds, sonicated for 30 seconds three times in a water bath, and incubated under agitation for 30 minutes at 4°C. After addition of 250µl of water, the samples were vortexed for 1 minute and centrifuged at 14,000g for 5 minutes at 20°C. The upper phase containing the lipids was collected and dried down under nitrogen. The dry extracts were reconstituted with 150µl of 9:1 methanol:toluene.

Lipid extracts were analyzed in a randomized order using an Ultimate 3000 RSLC system coupled with a Q Exactive mass spectrometer (Thermo Fisher Scientific) as previously described.[139] Each sample was run twice in positive and negative ionization modes and lipids were separated using an Accucore C30 column 2.1x150mm, 2.6µm (Thermo Fisher Scientific) and mobile phase solvents consisted in 10mM ammonium acetate and 0.1% formic acid in 60/40 acetonitrile/water (A) and 10mM ammonium acetate and 0.1% formic acid in 90/10 isopropanol/acetonitrile (B). The gradient profile used was 30% B for 3min, 30–43% B over 5min, 43–50% B over 1min, 55–90% B over 9min, 90-99% B over 9min and 99% B for 5min. Lipids were eluted from the column at 0.2ml/min, the oven temperature was set at 30°C, and the injection volume was 5µl. Autosampler temperature was set at 15°C to prevent lipid aggregation.

LC-MS peak extraction, alignment, quantification, and annotation was performed using LipidSearch software version 4.2.21 (Thermo Fisher Scientific). Lipids were identified by matching the precursor ion mass to a database and the experimental MS/MS spectra to a spectral library containing theoretical fragmentation spectra. The following lipid ions were used for quantification: [M+H]+ for ceramides (Cer), (lysophosphatidylcholine) LPC, phosphatidylcholine (PC), monoglycerides (MG) and sphingomyelins (SM); [M-H]- for phosphatidylethanolamines (PE), phosphatidylinositols (PI), phosphatidylserines (PS), phosphatidylgylcerols (PG) and lysophosphatidylethanolamine (LPE); and [M+NH4]+ for cholesterol ester (ChE), diglycerides (DG) and triglycerides (TG). To reduce the risk of misidentification, MS/MS spectra from lipids of interest were validated as follows: 1) both positive and negative mode MS/MS spectra match the expected fragments, 2) the main lipid adduct forms detected in positive and negative modes agree with the lipid class identified, 3) the retention time is compatible with the lipid class identified and 4) the peak shape is acceptable. The fragmentation pattern of each lipid class was experimentally validated using lipid internal standards.

Single-point internal standard calibrations were used to estimate absolute concentrations for 431 unique lipids belonging to 14 classes using one internal standard for each lipid class. Importantly, we ensured linearity within the range of detected endogenous lipids using serial dilutions of internal standards spanning 4 orders of magnitude. Median normalization (excluding TG and DG) was employed on lipid molar concentrations to correct for differential quantity of starting material. Importantly, we verified that median lipid signal (excluding TG and DG) correlated well (Pearson's correlation coefficient = 0.48, P = 0.005) with the total protein content in each sample as measured by the BCA Protein Assay Kit (Pierce, cat# 23225) from precipitated proteins following the biphasic separation, suggesting good sample quality. One development (diapause escape) sample had an unexpectedly low protein concentration and thus was discarded. Lipid molar concentrations for a given class were calculated by summing individual lipid species molar concentrations belonging to that class. Fatty acid composition analysis was performed in each lipid class. Fatty acid composition was calculated by taking the ratio of the sum molar concentration of a given fatty acid over the sum molar concentration across fatty acids found in the lipids of the class. Subsequently, saturated fatty acids (SFA), mono-unsaturated fatty acids (MUFA) and poly-unsaturated fatty acids (PUFA) were grouped together for comparative analysis.

Lipid droplet imaging

Embryos were imaged with a Zeiss confocal microscope (LSM900, Axio Observer) equipped with the Zen software (3.0, blue). Within each experiment, the same laser power and settings were used across all conditions. For whole embryo imaging a 5x air objective (Fluar 5x/0.25 M27) and a 22 μm pinhole were used to image the 20μm depth (5 slices, 5μm intervals, dorsal-to-ventral stack) of the fish. Z-stack projections were generated in Fiji version 2.0.0.[140] For zoomed-in visualization of lipid droplets a 63x oil objective (Plan-Apochromat 63x/1.40 Oil DIC M27) and a 32 μm pinhole were used to image the lipid droplets over a range of 0.72 μm (5 slices, 0.18 μm). Z-stack projections were generated in Fiji version 2.0.0 (Figures 7E and S7F).

For lipid droplet quantification, a 20x air objective (Plan-Apochromat 20x/0.8 M27) and a 32 μm pinhole was used to image the embryos in at least 3 different positions along their body over a range of 4 μm (3 slices, 2 μm intervals, dorsal-to-ventral). Lipid droplet number was quantified by generating z-stack projections of 3 slices, subtracting the background, applying the same threshold to all images, and quantifying the lipid droplet number in a 100 x 100 μm^2 area using the analyze particle function in Fiji version 2.0.0. The lipid droplet number was averaged across all locations imaged for one individual. For each condition, at least 3 embryos were imaged. Experiments were carried out at least two times independently. The lipid droplet number was normalized to the "Diapause 1 month" condition of the respective experiment, all experiments are plotted together in Prism 9 and statistically significant differences between samples were assessed using a Kruskal-Wallis test for differences in mean (Figure 7F). See Table S7D for unprocessed lipid droplet numbers and statistical differences (Mann-Whitney U-test) within one experiment.

QUANTIFICATION AND STATISTICAL ANALYSIS

Identification and dating of paralogs

We focused our analysis on paralogs because i) gene duplication or paralogs are the primary mechanism by which new genes originate and specialize for new functions or states[12,13]; ii) paralogs also allow for a precise timing of the evolutionary origin of specific genes, and iii) the majority of genes in killifish are in paralog pairs owing to multiple rounds of genome duplicates. To generate a comprehensive resource of paralogs in multiple killifish species and to date their duplication time relative to other species, we used the OrthoFinder pipeline.[15,100] To this end, we collected genome sequences from multiple killifish species with and without diapause from published reports and NCBI genome,[8,9,29] other teleost fish, mammals, and non-vertebrate outgroups from Ensembl (version 100).[141] Phylogenetic tree-based inference of orthologs, paralogs, and relative duplication timing of each paralog in all these species was done by OrthoFinder. OrthoFinder infers "groups" of genes or gene families including both ortholog and paralog for all species used in the analysis (called *orthogroups*). Gene trees were built for all these *orthogroups* and reconciled with the rooted species tree to identify gene duplication events and their relative duplication time based on a phylogenetic approach.[15,100] Note that for a single species in an *orthogroup*, one gene can be a paralog partner with multiple other genes making groups of paralogs. We filtered out the paralog groups with >20 paralog partners for a gene to exclude large inter-connected paralog groups, which can inflate the overall pairwise analysis. Note also, that our results were not dependent on the paralog group or family size (Figures S1D–S1F; Table S1D). Duplication node and approximate timing of the duplication (in million years ago [mya]) for each paralog pair was annotated based on known phylogenetic tree from Ensembl for species covered in Ensembl version 100[141] or published reports for killifish species.[14] To ensure that our results were not affected by the choice of species and outgroups used, we used 3 different sets of species to run the complete OrthoFinder pipeline independently: a set of 71 species, 31 species, and 13 species. The three pipelines resulted in very similar estimates of relative duplication time for killifish paralogs and the results were qualitatively identical (Table S1D). We used paralogs identified by OrthoFinder analysis with 71 species for our study (20,091 paralog pairs in African turquoise killifish, *Nothobranchius furzeri*, 22,955 pairs in the South American killifish, *Austrofundulus limnaeus* genomes), and 13,437 pairs in mouse, *Mus musculus*, genome.

In addition to OrthoFinder, we also annotated the paralog duplication timings in the African turquoise killifish directly from Ensembl version 84 using an independent approach. To identify the paralog pairs in the African turquoise killifish genome, we first identified high confidence one–to-one orthologs (bi-directional best hits) between the African turquoise killifish and each of the 5 teleost fish species (zebrafish, *Danio rerio*; medaka, *Oryzias latipes*; stickleback, *Gasterosteus aculeatus*; tetraodon, *Tetraodon nigroviridis*; and fugu, *Takifugu rubripes*) using BLASTp (E-value 1e-03).[101] We next identified paralogs in each of the five teleost fish for which both the genes had one-to-one orthologs in African turquoise killifish, and assigned their duplication time to the African turquoise killifish paralog. Because Ensembl did not have any killifish species, the paralogs duplicated in the killifish lineages after the divergence from medaka would be missed. Therefore, to identify such paralog pairs, we performed a protein family clustering using all the protein coding genes for multiple killifish species with and without diapause along with other teleost fish. We then annotated the duplication time for each of the potential paralogs that were not already identified using the ortholog analysis as "teleost" (if they were shared with the other teleost fish), "aplocheiloidei" (i.e. common ancestor of all killifish, if it was shared only by killifish species without diapause), and "nothobranchius" or "*Nothobranchius furzeri*" (shared by nothobranchius genus or only present in the African turquoise killifish, respectively). This independent pipeline also resulted in very similar estimates of relative duplication time for killifish paralogs and the results were qualitatively identical (Table S1D). A total of genes that were not observed in any of our four paralog analysis pipelines (OrthoFinder with 71, 31, or 13 species or with Ensembl) were classified as singleton genes.

To simplify the interpretation and analysis, the relative duplication nodes from each analysis were divided into 3 categories: *very ancient* (paralogs duplicated in the ancestor of jawed vertebrates at nodes Gnathostomata and earlier i.e. >473.3 mya), *ancient*

(paralogs shared by most teleost fish species, duplicated between nodes Ovalentaria and Gnathostomata at 111-473.3 mya or earlier), *recent* (paralogs shared by most killifish species, duplicated between nodes Ovalentaria and *Nothobranchius furzeri* at < 111 mya)[141] (Figures 1D and S1D–S1F). Diapause-specialized paralog numbers (see below) in each of the three categories were compared to the genome average in that category with 10,000 bootstraps resampling of 50% paralogs genome-wide (Figures 1E, 2E, S1D–S1F, and S2H). For mouse diapause, *very ancient* paralogs pairs were defined similar to killifish, *recent* paralogs were shared by all the mammals in our data and *recent* paralogs were shared only by eutherian mammals (Figure S2H).

Classifying paralogs specialized for diapause

To identify the African turquoise killifish paralog pairs that show signs of specialization of the gene expression pattern for diapause, we used the normalized RNA-seq expression from Hu et al.[6] (see below). This dataset consists of two stages during African turquoise killifish development (heartbeat onset and diapause escaped embryos 1-day post heartbeat onset) and three time points during diapause (diapause embryos at 3 days, 6 days, and 1 month in diapause). We first identified differentially expressed genes in all three diapause time points with respect to both development time points using DESeq2 (version 1.30.1).[102] A paralog gene pair was classified as having specialization of expression if one gene was significantly upregulated in one of the three diapause time points (FDR < 0.05) with respect to one of the two development stages, and the other partner gene was significantly downregulated in diapause or had a median expression in development higher than median expression in diapause. This resulted in 6,247 paralog pairs with expression specialization in diapause with the 71 vertebrate OrthoFinder pipeline (Table S1C). To test robustness, we used several different criteria to identify diapause-specialized paralogs (different FDR cutoffs, and different combinations of differentially expressed genes). To test robustness, we used several different criteria to identify diapause-specialized paralogs (different FDR cutoffs, and different combinations of differentially expressed genes), and our results were robust to the changes in FDR cutoffs.

We independently identified paralogs specialized for South American killifish diapause, using RNA-seq data of South American killifish embryos in diapause and development (4 days post diapause exit) from Wagner et al.[9] Paralogs with one gene significantly expressed (i.e., upregulated) in diapause compared to development (FDR < 0.05), and the other gene significantly downregulated in diapause compared to development (FDR < 0.05) were classified as specialized paralogs (2,480 pairs). Note that the diapause and post-diapause development stages are not an exact match to the African killifish stages, these stages are within a similar timing window and separate together by PCA (see Figure S2A)

Paralogs specialized for mouse diapause were identified using RNA-seq data of mouse embryos in *diapause* (Pre-implantation, diapause blastocyst) and development (pre-implantation Inner Cell Mass (*ICM*), day 3.5 post-fertilization; and post-implantation epiblasts (*Epi*), day 6.5) from Hussein et al.[22] The RNA-seq data was reanalyzed using the mouse reference genome[97] and the same processing pipeline as the African and the South American killifish (see below). Paralogs with one gene significantly upregulated in diapause compared to *ICM* and *Epi* (FDR < 0.05), and the other gene down in diapause compared to both *ICM* and *Epi* were classified as specialized paralogs (201 pairs). The lower numbers of specialized pairs in diapause are likely due to less extreme nature of mouse diapause compared to killifishes.

Assessing paralog divergence and location

The set of paralog pairs described above were aligned and the rate of synonymous (dS) and non-synonymous (dN) mutations were evaluated using the PAML package (v4.8). The ratio dN/dS (or omega ratio; ω) was calculated between each pair, assessing their difference in sequence from one another as opposed to their changes from an outgroup species or common ancestor. We then used this list of single ω-ratio per-pair to evaluate any difference in sequence divergence between the genome-wide paralog set and paralog pairs identified with specialized expression for diapause. Diapause specialized paralogs were detected to have significantly less sequence divergence than their genome-wide counterparts (Mann-Whitney U test, $P = 2.2e-16$) (Figure S1J, left). This significant difference was also observed when subsetting the paralog pairs by time of duplication in both the very ancient ($P = 2.12e-04$) and ancient ($P = 9.779e-09$), and trending in the recent/very recent category ($P = 0.0619$) (Figure S1J, center-left to right, respectively).

Additionally, we assessed the chromosomal locational of each of our defined paralog pairs. The pairs were divided into two groups: 1) paralog pairs in which both members are located on the same chromosome and 2) the paralogs are on different chromosomes. When examining the genome-wide distribution, a majority of paralog pairs were found on separate chromosomes in the African turquoise killifish. However, there were significantly less diapause-specialized paralog pairs that are located on the same chromosome than the genome average (Mann-Whitney U-test, $P = 4.71e-12$) (Figure S1G). This difference was also observed when partitioning the paralog pairs by age of duplication in both the very ancient ($P = 2.257e-02$) and ancient ($P = 2.627e-07$) (Figure S1H, left and center). However, this difference was not observed in the recent/very recent paralog group, which are roughly equally distributed in tandem and on separate chromosomes ($P = 0.7498$) (Figure S1H, right).

All these paralogs, along with their expression level, genomic locations, and specialization in diapause are included in Table S1C.

RNA-seq data processing pipeline

We first trimmed the adaptors from raw sequencing FastQ files using Trim Galore (version 0.4.5) followed by read quality assessment using FastQC[103]

(version 0.11.9) and MultiQC (version 1.8).[104] Adaptor trimmed files were aligned to the respective genome (Table S1A) using STAR (version 2.7.1a).[105] No reference genome is available for the red-striped killifish, so the reads from red-striped killifish RNA-seq libraries were aligned to the genome of its close relative, lyretail killifish. Identification of accurate gene expression values for paralogs can be challenging if the reads align to both the genes in the pair equally well. Therefore, we excluded all the reads that mapped to multiple locations in the genome, and only kept reads that align uniquely to a single genomic locus with samtools (version 1.5) using "*samtools view -q255*" command. Read counts were then assessed using featureCounts function in Subread package (version 2.0.1).[106,142] Raw gene expression values were then normalized using DEseq2 (version 1.30.1).[102] Because different RNA-seq datasets were generated separately, we performed separate normalization for each of the individual analyses.

ATAC-seq data processing pipeline

To process ATAC-seq, we first removed adaptors from FastQ files using TrimGalore (version 0.4.1), followed by read quality assessment with FastQC[103]

(version 0.11.9) and MultiQC (version 1.8).[104] Reads were then aligned to their respective reference genomes (Table S1A) using BowTie2 (version 2.2.5)[107] with "*–very-sensitive*" option. No reference genome is available for the red-striped killifish, so the reads from red-striped killifish ATAC-seq libraries were aligned to the genome of the closest sequenced species, lyretail killifish. Duplicates were marked using Picard (version 2.22.1). Duplicates, multimapping reads (MAPQ < 20), unmapped and mate-unmapped reads (only one read of the pair mapped), not primary alignments, and reads failing platform were then removed using SAMtools (version 1.5).[108] Because the Tn5 transposase binds as a dimer and inserts two adaptors separated by 9bp, all aligned read positions on + strand were shifted by +4bp, and all reads aligning to the – strand were shifted by −5bp, using alignmentSieve in deepTools (version 3.2.1).[23,109] We called peaks using MACS2 (version 2.1.1.20160309)[110,143] using different effective genome size for each species (e.g., genome size after removal of gaps represented by Ns).

Library quality was assessed using metrices recommended by ENCODE consortium including fragment length distribution to assess nucleosome banding patterns and enrichment of ATAC-seq peaks at transcription start sites. We observed the nucleosome banding patterns strongly in many of ATAC-seq libraries, though some lacked strong indication of classical band spacing of nucleosomes. We believe this is due to the particularly fragile nuclei/chromatin structure of killifish embryos (requiring orders of magnitude less transposase enzyme to yield efficient cutting. This led to some libraires being 'over-transposed'. To address if these libraries were still sufficient quality for downstream analysis, we evaluated other metric to assess library quality such as transcription start site read enrichment, PCR bottleneck coefficients (PBC1 and PBC2), and fraction of reads in peaks (FRiP) (Table S3A). There was a significant enrichment of ATAC-seq peaks at transcription start sites as expected (Figures S3F and S3G). Other quality metrices were also above the threshold recommended by the ENCODE consortium (Table S3A).

ATAC-seq data from medaka and zebrafish for corresponding development stages were obtained from Marlétaz et al.[24] (Table S1B) and processed using the same pipeline described above. We used development stage 19 and 25 in medaka and 8-somites and 48 hours post fertilization in zebrafish, which are expected to correspond to pre-diapause and development in the African turquoise killifish respectively. These were used for chromatin accessibility conservation analysis presented in Figures 3, 4, 5, and S3–S5.

Multiple whole-genome alignment

To integrate ATAC-seq and RNA-seq datasets across species, we performed a 5-way multiple whole-genome alignment with African turquoise killifish (*Nfur: Nothobranchius furzeri*), lyretail killifish (*Aaus: Aphyosemion australe*), South American killifish (*Alim: Austrofundulus limnaeus*), medaka (*Olat: Oryzias latipes*) and zebrafish (*Drer: Danio rerio*) (Table S1A), using African turquoise killifish as the reference genome. For red-striped killifish (*Aphyosemion striatum*), genome of the closest sequenced species lyretail killifish was used for integrative analysis. For genomes with chromosome level assemblies, we discarded scaffolds not placed on chromosomes. First, we performed pairwise alignments between African turquoise killifish and each of the four other fish genomes using LASTZ[111] (parameters: –gap=400,30 –gappedthresh=3000 –ydrop=6400 –inner=2000 –hspthresh=1500 –masking=50 –no-transition –step=20 –scores=HoxD55.q). Subsequent chaining and netting were performed using the suite of UCSC genome browser utilities.[112] The percentage of aligned African turquoise genome to each of the other fish species decreased based on the distance to the last common ancestor as expected[144] with 61.1%, 47.8%, 23.2%, 20.1% of the African turquoise killifish genome aligning to the lyretail killifish, South American killifish, medaka and zebrafish genomes respectively in a pair-wise manner.

These pairwise alignments were then merged using the multi-alignment tool Multic/TBA,[113] using the command *<tba + E=Nfur ((((Nfur Aaus) Alim) Olat) Drer./pairwise_dir/>* to obtain a single, 5-way, multiple whole-genome alignment using the African turquoise killifish genome as the reference (specified by *E=Nfur*). The resulting multiple-whole genome alignment covered ~75.3% of the African turquoise killifish genome. Coverage of each of the aligned fish genome in the multi-alignment also diminished as time to the last common ancestor increased with 62.7%, 85.9%, 14.2%, and 23.7% of the genome being covered for lyretail killifish, South American killifish, medaka, and zebrafish genomes respectively.

To assess the quality of our genome alignment, we compared the length of aligned sequence blocks in multi-genome alignment with that of teleost fish 8-way multi-genome alignments available from the UCSC genome browser and generated using a similar approach[145] (https://hgdownload.soe.ucsc.edu/goldenPath/danRer7/multiz8way/). We found that the aligned block lengths in both our and 8-way multi-genome alignment from UCSC were comparable. Most of the aligned blocks were either 10-99bp long

(53% our vs 38.7% UCSC-fish) or 100–999bp long (33% our vs 26.5% 8-way alignment from UCSC) in both the alignments. Importantly, a vast majority of our ATAC-seq peaks (98.35% of chromosomal peaks) fall in the regions that are covered in our multi-genome alignment.

Integrating ATAC-seq across species

The 5-way multiple whole-genome alignment was used to compare ATAC-seq data across species. Bed files for each ATAC-seq library were cross-referenced to the alignment and the coordinates of ATAC-seq peaks for all species were converted to African turquoise killifish genome coordinates. During this process, peaks were tagged as "conserved" at three levels of stringency: relaxed (any base pair overlap between peaks), strict (25% of the African turquoise killifish peak must be covered by aligned peak region in other species), and very strict (50% of the African turquoise killifish peak must be covered by the aligned peak in other species). The differences in peak conservation between relaxed and strict definitions was qualitatively minimal. Thus, subsequent analysis was performed with the relaxed peak set. During coordinated conversion, some peaks for species other than African turquoise killifish became split between two or more locations in the African turquoise killifish genome. We also included these split location peaks in our analyses. However, split location peaks represent only a minority of recovered peaks (5.2%) and are unlikely to influence our analyses.

With this finalized peak set, we then categorized each peak in African turquoise killifish and its underlying sequence into one of three conservation categories: *ancient/very ancient, recent, and very recent*. 1) Peaks considered *very recent* had only a peak in the African turquoise killifish (likely originated after divergence from killifish species without diapause at < 17.79 mya)[14]). 2) Peaks considered *recent* had overlapping peaks in African turquoise killifish and at least one other African killifish (i.e. lyretail killifish or red-striped killifish), but not in outgroups (medaka and zebrafish; likely originated between 17.79–93.2 mya).[14,141] 3) Peaks considered *ancient/very ancient* had overlapping peaks in African turquoise killifish, at least one other African killifish (i.e. lyretail killifish or red-striped killifish), and at least one outgroup fish (i.e. medaka or zebrafish; likely originated > 93.2 mya)[141] (Tables S3B and S3C). To avoid confounding peaks within our *very recent* category, peaks present in the African turquoise killifish, absent in other African killifish, yet present in either zebrafish or medaka were subsequently added to the *ancient/very ancient* category despite being just outside of the above parameters. The same criteria were used to define sequence conservation. However, instead of requiring accessible-chromatin overlap, sequences were evaluated for having an aligned orthologous region in each species.

To visualize these peaks across species, we used the Integrative Genomics Viewer (IGV).[114] For each species, RPKM-normalized read counts were used either directly (paralog displays) or summed across replicates and across developmental/diapause stages (for single displays) to create single coverage tracks for fish without diapause and two tracks (one diapause, one development) for fish with diapause. Tracks from each species were then anchored to each other via a single conserved base in the multiple-whole-genome-alignment and extended to the exact same window size in all species. The anchor point for each peak region was chosen based on its proximity to the summit of the peak in the African turquoise killifish. Track height for each species was set automatically by IGV using either the height of the peak of interest, or, in species without a conserved peak, to the height of the tallest peak within 40kb of the anchoring base pair. These visualizations illustrate the conservation and specialization states described above.

These analyses revealed that for the majority of peaks, the genome sequences under chromatin accessible peaks are 'alignable' (i.e. conserved enough to establish orthology at the genome-wide level), but chromatin accessibility at those regions evolved very recently and exclusively in the African turquoise killifish. This pattern was consistent for genome-wide chromatin, chromatin associated with singleton genes (Figures S3C and S3D). The sequence conservation is also strongest at coding sequence (exons) and decays as expected across promoters, UTRs, introns, and intergenic regions (Figure S3E).

ATAC-seq Principal Component Analysis (PCA)

To explore the global relationships between killifish ATAC-seq samples, we performed principal component analysis (PCA) using ATAC-seq peak intensities (normalized aligned read counts for each peak). To this end, we first generated peak intensity matrices for each of the following comparisons: 1) for the African turquoise killifish diapause and development samples (Figure 3B, upper-left); 2) for the South American killifish species (Figure 3B, upper-right), 3) for all killifish species (African turquoise killifish, South American killifish, lyretail killifish and red-striped killifish, Figure 3B, lower-left); 4) killifish with diapause (African turquoise killifish and South American killifish, Figure 3B, lower-right). For each comparison, the peak matrix contained VST-normalized peaks intensities for all consensus peaks detected in all the samples in that comparison. Cross-species comparison only included the peak(s) conserved in all samples. The total peaks used for PCA were 60,359 for the African turquoise killifish, 1,293 for all killifish, and 3,721 for killifish with diapause. PCA plots were done using autoplot command in ggfortify (version 0.4.11) package[115] in R (version 3.6.2).

Diapause differential peak analysis

To identify ATAC-seq peaks that are specific to diapause in the African turquoise killifish genome, we performed a differential peak accessibility analysis pairwise between the two developmental conditions (pre-diapause and non-diapause) and the two diapause conditions (diapause at 6 days and 1 month time points) using DiffBind (version 2.16.2).[116,146] We used both DESeq2[102] and edgeR[117] algorithms implemented in DiffBind for differential accessibility analysis. Diapause specific peaks were then identified as the peaks that were significantly up (chromatin more open) in any of the two diapause conditions with either DESeq2 or edgeR, but do not significantly change (up or down) between the two development conditions with both DESeq2 and edgeR. This led to 6,490 chromatin

peaks genome-wide in African turquoise killifish and 6647 chromatin peaks genome-wide in South American killifish that are significantly up in diapause but do not change during development (Figure S3A; Tables S3B and S3C). Peaks were assigned to their nearest genes using ChIPseeker (version 1.28.3),[118] to identify 1,880 diapause specific peaks at specialized paralogs in African turquoise killifish (Table S3B) and 8166 diapause specific peaks at specialized paralogs in South American killifish (Table S3C). Peak annotation with the genomic properties was also performed using ChIPseeker (Figure S3B). These peaks at specialized paralogs were used for motif enrichment and peak conservation analyses presented in Figures 3, 4, 5, and S3–S5.

Motif enrichment and conservation

HOMER (version 4.10), was used for transcription factor binding site enrichment analysis,[27] using the ATAC-seq peaks that are significantly up in diapause and were in proximity to the diapause specific paralogs for the African turquoise killifish and their orthologous conserved peaks in other species. Genomes of all the species were added to HOMER using "*loadGenome.pl*" utility with the genome fasta and GFF files as input (Table S1A). We then used the genomic coordinates from the bed file for the diapause specific ATAC-seq peaks at paralogs as input to "*findMotifsGenome.pl*" and specified vertebrate motifs by "*-mset vertebrates*". Known motifs in "*knownResults.txt*" generated by the HOMER output was used for all the analyses. To remove redundancy in motifs, we performed a motif clustering using tomtom utility in the MEME suit (version 5.3.0)[119,147] using the following parameters: *-thresh 1e-5 -evalue -min-overlap 6*. The resulting clusters were manually curated, and motifs (binding sites) were assigned to the genes coding for the transcription factors.

TF binding sites across species

To assess the evolution and conservation of African turquoise killifish diapause-specific transcription factor binding sites at specialized paralogs in other species, we extracted sequences of these motifs from African turquoise killifish and the corresponding aligned sequences in other species from our 5-way multiple whole-genome alignment. We observed that a vast majority of transcription factor binding motifs that are enriched in ATAC-seq peaks up in diapause at specialized paralogs in the African turquoise killifish are aligned in other species with motif-like sequences (i.e. sequences similar to the canonical motifs). To assess if these motif-like sequences are likely to be bound by their respective transcription factors, we subjected motif or motif-like sequences to a binding likelihood calculation identical to that used by HOMER.[27] We then determined if motif-like sequences in species other than African turquoise killifish met the log odds detection threshold (defined as the $\log(X_1/0.25) + \log(X_2/0.25) +\ldots \log(X_n/0.25)$ where X is the probability of a given base being present at a given location in a given motif) computed by HOMER[27] during motif enrichment, which is used to determine likelihood of transcription factor bound vs. unbound sites. We also excluded motif sites in peaks where an identical motif was found near the aligned region in another species. This allowed us to detect cases where the sequence directly aligned to a motif is not conserved, but the motif is present nearby and possibly providing similar regulatory potential.

These analyses revealed that a very low number of motif-like sequences in other species are expected to bind the transcription factor at that position and can be considered as conserved transcription factor binding sites across species (4.77% on average). Thus, the vast majority of these motif-like sequences were likely used as 'substrates' during evolution for mutation and selection of canonical motif sequences for binding of transcription factors (Figures 5C, 5D, S5A, and S5B). The same approach was used to compare motifs in conserved accessible chromatin (Figure S4C).

To compare motif between the African and the South American killifish diapause in an alignment independent manner, we focused on diapause-specific accessible chromatin peaks at specialized paralogs in the two species. We performed two independent motif enrichment analyses using these peak sets independently and compared the significantly enriched motifs in at least one of the species (Figure S4D). To compare the convergent evolution of motifs in the African and the South American killifish in an alignment independent manner, we focused on the diapause-specific chromatin accessible peaks at specialized paralogs independently in the African and South American killifish. We then identified the one-to-one orthologs of these specialized genes in other killifish without diapause (lyretail killifish, red-striped killifish, medaka and zebrafish) and identified all the peaks closest to these ortholog genes. Because there is no diapause in these other killifish species, we down sampled these peaks to the same number as diapause-specialized peaks in the African turquoise killifish, keeping the same composition of peaks (e.g. promoter, intronic, intergenic etc.). We then performed the motif enrichment analysis and comparison in these peaks (Figure S4E).

To identify the subsets of paralogs controlled by each diapause-specific binding sites, we examined the subset of specialized paralog pairs that have a differentially accessible peak containing an enriched TF binding site (Figure S6D).

Transposable element analysis

To evaluate the contribution of Transposable Elements (TEs) for the evolution of diapause, we first developed a comprehensive map of abundance and genomic location of all TEs in the aforementioned teleost fish species used to construct the genome multi-alignment. We employed RepeatMasker (version 4.0)[120] to identify repetitive sequences using the *Teleosti* suite of know repeat elements <*Repeatmasker -a -s -species 'Teleostei' Input.fa*> and < *processRepeats -xsmall* RMoutput.fa.gz> allowing for a standardized repetitive element set across species. We detected similar abundances of TE classes and families as previously reported by various sources.[148] We then identified overlap between all ATAC-seq peak coordinates and TE coordinates in African turquoise killifish. We evaluated TE enrichment at ATAC-seq peaks up specifically in diapause as compared to: 1) 'Genome': TE representation genome-wide (Figure 5F, upper), 2) 'Chromatin': TE representation within all ATAC-seq peaks (Figure 5F, middle), 3)

CellPress
OPEN ACCESS

'Control loci': size-matched regions 10kb downstream of ATAC-seq up specifically in diapause (Figure 5F, lower), using a binomial test (Mutational Patterns Package version 3.2.0).[121] Several TE families showed enrichment specific to differentially accessible chromatin sites specific to diapause, such as Crypton-A (DNA), Zisupton (DNA), RTE-X (LINE), and tRNA-Mermaid (SINE) (Figure 5F).

We then evaluated the overlap between these TE instances and enriched transcription factor binding motifs detected in our analysis above. These chromatin-accessible TE-embedded motifs were also evaluated for conservation across species by assessing whether 1) the TE is present at aligned location in the genome alignment and contains the transcription factor binding motif sequence, 2) the TE is present at the aligned location in other species, but lacks the transcription factor binding motif sequence, 3) the TE is absent at the aligned location, but a transcription factor binding motif still exist at this location in the alignment, or 4) both the TE and transcription factor binding motif binding site are absent at the aligned location in the other species. This analysis revealed that a majority of TE sites are exclusive to African turquoise killifish, as can be expected given the rapid rate at which the TE landscape changes and given the recent TE expansion in the African turquoise killifish genome.[29,149]

Positive selection of regulatory regions

To evaluate whether diapause-accessible chromatin peaks show any signature of positive selection, we used a recently developed method to detect positive selection at transcription factor binding sites and accessible chromatin.[28,150] We scanned for signature of positive selection at the genomic DNA underlying ATAC-seq peaks with respect to: 1) ancestor of all killifish species in our analysis ('killifish ancestor'); and 2) ancestor of killifish and medaka ('pre-medaka ancestor') (Figure S6A). We first inferred ancestral sequences for these two nodes within the teleost lineage using the PAML package (version 4.8).[122] Alignment blocks from our 5-way fish multiple whole-genome alignment that were at least 50bp long and covered at least 50% of the ATAC-seq peaks were used for the ancestor generation and positive selection analysis. We excluded ATAC-seq peaks that were in exons to focus on regulatory elements. The ancestral sequences and the African turquoise killifish sequences were used to generate Support Vector Machine (SVM) kmer weights and positive selection was detected using hightail test as recommended[28,150] (https://github.com/ljljolinq1010/A-robust-method-for-detecting-positive-selection-on-regulatory-sequences/). The Benjamini-Hochberg procedure was used for multiple hypothesis correction, and ATAC-seq peaks with FDR < 0.1 for either pre-killifish or pre-medaka ancestors were considered to be under positive selection (Table S3B).

In total, we detected 3,836 and 3,928 ATAC-seq peaks with signature of positive selection using the 'killifish ancestor' and 'pre-medaka ancestor' inferred sequences respectively, with both having a strong overlap of 3,370 (76.7%) (Figures S6A and S6B). We used the union of the two groups for the downstream analysis. A total of 172 diapause-specific ATAC-seq peaks at specialized paralogs showed signature of positive selection (Figure 5E; Table S3B). These were enriched for several of the transcription factor binding motifs detected in our previous analysis, including REST, FOXO3 and PPARs (Figures 5E and S6C). The functional enrichment of ATAC-seq peaks also included several functions related to lipid metabolism (Table S4A). These results suggest that at least a portion of genomic loci underlying diapause-specific ATAC-seq peaks may have evolved due to positive selective pressure at these loci.

Positive selection on protein-coding genes

The protein-coding genes under positive selection in the African turquoise killifish were identified using phylogenetic analysis involving 19 fish species with and without diapause as described in Wagner et al.[9] Briefly, protein sequences were clustered using Proteinortho (version 5.11),[123] followed by filtering of clusters and alignment of coding sequences of the filtered clusters using PRANK v.140603.[124] The resulting codon aware alignments were filtered with GUIDANCE v2.0[125] to remove low quality regions. Proteins and individual amino acids under positive selection were then identified in either the ancestor of African killifish species with diapause (in the branch leading to the African killifish genus nothobranchius after separation from the African killifish without diapause *A. striatum*) or the branch leading to the African turquoise killifish only, using the branch-site model in CODEML implemented in the Phylogenetic Analysis by Maximum Likelihood package (PAML).[122] Notably, the ancestral branch co-insides with the time period at which evolution of diapause likely occurred in African turquoise killifish (~18 mya). Proteins with a *P*-value of the branch-site test less than 0.05 (without any FDR correction to maximize the number of proteins with potential signals of selection) were then filtered. We used the union of proteins under positive selection identified using both the ancestral and the African turquoise killifish branch. This led to a list of 277 protein-coding genes under positive selection in the ancestor of killifish species with diapause after divergence from killifish species without diapause and outgroup fish species (Figure S1I).

Functional enrichment analysis

To perform functional enrichment analysis for diapause specific African turquoise killifish ATAC-seq peaks or upregulated genes in diapause, we used Gene Ontology (GO) analysis using GOstats package (version 2.56.0).[126] GO terms from human and zebrafish were assigned to their killifish orthologs (best hit protein with BLASTp E-value >1e-3). For GO enrichment analysis using diapause specific ATAC-seq peaks, we used the non-redundant list of genes closest to the peaks (Table S3B) with all protein coding genes as background and performed a hypergeometric test implemented in GOstats. Similarly, for RNA-seq, we used genes upregulated in diapause (Table S1C). GO terms enriched in both diapause RNA-seq and ATAC-seq included many GO terms related to lipid metabolism (Tables S2A, S2B, S4A, and S7A). We also performed GO enrichment analysis for the subset of ATAC-seq peaks that show signatures of positive selection (see above, Table S4A), and observed that several lipid metabolism related functions are enriched in the genes next to the chromatin accessibility regions that have evolved under positive selection (Table S4A). For GO terms share

across species, we performed independent enrichment analyses using the genes significantly upregulated during diapause in the African turquoise killifish, the South American killifish and mouse using the same approach (Table S2A).

To identify the upstream regulators of genes upregulated during diapause in the African turquoise killifish, we used Ingenuity Pathway Analysis (IPA) upstream regulator analysis (QIAGEN, March 2021 release) (Table S7B).

TF knockout selection

To select key transcription factors and test their functional role in the diapause program, we integrated both our ATAC-seq and RNA-seq data to generate a list of top candidates. A transcription factor was included in our list of top candidates if the binding site of this transcription factor was enriched in chromatin regions that become differentially accessible (e.g., 'open chromatin') during diapause (but not during development), and if the expression of this transcription factor was significantly higher in at least one diapause time-point compared to development. For each candidate, we verified that there was a clear ortholog between mammals and killifish. We then prioritized candidates, considering the novelty or conservation of a transcription factor for a role in a suspended animation phenotype as well as their connection to functions that could be relevant in diapause (e.g., lipid metabolism, stress response, etc.). We also included paralogs of the selected transcription factors, as they may target the same binding sites. Our final list had 6 candidates: REST, FOXO3a, FOXO3b, PPARAa, PPARAb, and PPARG (Table S5A). Other transcription factors such as NR2F2 and TEAD2 were not as strong candidates: while their binding sites are enriched in diapause-accessible chromatin, the *NR2F2* gene is actually downregulated in diapause and TEAD2 does not have a clear ortholog in killifish.

sgRNA design

We selected single guide RNAs (sgRNAs) for each of these genes using the CHOPCHOP[127] online guide design platform (https://chopchop.cbu.uib.no/). For each gene, we selected 3 sgRNAs that fit 3 different criteria: 1) the sgRNA needed to target multiple exons in the beginning of the candidate gene to increase the chance for an early stop codon, 2) the sgRNA needed to have a high predicted cutting efficiency of above 60%, and 3) the sgRNA was predicted to have no off-target sites or no alternative sites within a hamming distance of 1 within the genome (Table S5B). These criteria were used generate the top three sgRNA candidates for each gene and the sequences were generated with canonical linker/Cas9 domain for a complete sgRNA (Table S5B). sgRNAs were then synthesized using Integrative DNA Technology's (IDT) custom RNA-oligo ordering platform.

In addition to transcription factor targeting sgRNAs, we generated three GC content-balance sgRNAs whose sequence does not appear in the genome of the African turquoise killifish and is not within a hamming distance of 3 of any know genomic location (Table S5B). These scrambled sgRNAs were predicted to have no cutting sites and were synthesized using the same RNA-oligo generation service and gene-targeting guides.

Single embryo RNA-seq pipeline

We first trimmed the adaptors from raw sequencing FastQ files using Trim Galore (version 0.4.5) followed by read quality assessment using FastQC[103]

(version 0.11.9), and MultiQC (version 1.8).[104] Adaptor trimmed files were aligned to the African turquoise killifish genome (Table S1A) using STAR (version 2.7.1a).[105] For accurate assignment of reads across paralogs, we excluded all the reads that mapped to multiple locations in the genome, and we only kept reads that align uniquely to a single genomic locus with samtools (version 1.5) using "*samtools view -q255*" command.

Filtering of control and knockout RNA-seq

We used several criteria to filter uninformative RNA-seq libraries. For control libraries (non-injected [Wildtype] and scrambled sgRNAs [Scramble]), we used the results of Sanger-sequencing of embryo lysate described above for filtering. We removed the libraries that did not yield good sequences, as we could not evaluate genotypes; those represented a minority of samples (2 out of 58, e.g., 3.45%). Because no editing is expected in the control samples, we removed libraries for which the ICE knockout scores were predicted to be higher than the background expectation (>5% knockout prediction score in Wildtype/Scramble); these samples likely represent poor Sanger-sequencing quality or technical artifacts (Figure S6E). We also removed samples that had poor correlation with each other and likely represented technical differences between library preparation. These 3 filtering steps resulted 25 wildtype and 18 control samples, and this large number helps to overcome potential individual-to-individual variation.

For knockout libraries, we excluded libraries whose ICE knockout scores were below the cutoff for an expected 'majority-knockout' mosaic animal (<45% knockout prediction in Synthergo ICE for mutants) (Figure S6E). For knockout libraries, failure to generate Sanger-sequencing may also represent complex rearrangements, and we thus kept these samples for our final analysis. There were only 3 knockout samples (1 each for *REST* diapause, *REST* development and *FOXO3B* development) with a low correlation with other replicates. We did not filter them out, as the nature of mosaic knockout may lead to differences in transcriptional phenotypes, even when targeting the same gene. These filtering steps resulted in at least 3 samples per stage per genotype (minimum of 6 samples per knockout of transcription factor of interest). For these libraries, read counts were then assessed using 'featureCounts'[142] function in Subread package (version 2.0.1).[106] Raw gene expression values were then normalized using DEseq2 (version 1.30.1).[102] We further evaluated the status of mutations of the putative knockouts by assessing read pileup misalignment

and split-read alignment at the cut sites in each mutant (example in Figure S6F) and their general expression patterns across samples (Table S5C).

TF knockout RNA-seq Analysis

PCA was performed on the normalized read counts for each library using DESeq2 (Figure 6B). In addition, to represent the effect that each gene knockout had on the transcriptome we identified DEGs between each condition in a pairwise manner between Wildtype (non-injected embryos), Scrambled (embryos injected with scrambled sgRNAs), and transcription factor knockout libraries (TF KO) for both diapause and development using DEseq2. No significant DEGs were detected between Scramble and Wildtype samples at FDR < 0.1 (after multiple hypothesis correction using Independent Hypothesis Weighting; IHW approach[151]). To eliminate the transcriptional impact of injection, we marked a gene as DEG if it was significantly differentially expressed at FDR < 0.1 in both "TF KO vs Scramble" and "TF KO vs Wildtype" samples (after multiple hypothesis correction using Independent Hypothesis Weighting; IHW approach). We use as Control the intersection of Wildtype and Scramble samples. There was no significant overlap between the DEGs for each TFKO and predicted target genes of each transcription factor as determined by our ATAC-seq data, indicating that all these genes may not be the direct targets of these transcription factors.

TF knockout correlation plots

To overlay the effect of gene knockouts on diapause, we assessed the correlation between i) the log fold-change of differentially expressed (DE) genes between control diapause and development and ii) the log fold-change of DE genes between gene knockout diapause and control diapause (Figure 6C). Each knockout was then assessed for its impact of the diapause transcriptional program: no effect (no correlation), accentuated diapause-like program (positive correlation), or switch to a more development-like program (negative correlation). Spearman's ρ and P-values were calculated by the basic functions of R v3.6.2 (Figures 6D and 6E).

TF knockout GO enrichment

GO enrichment analysis for each TF-KO was performed using Gene Set Enrichment Analysis (GSEA) implemented in ClusterProfiler R package[152] after ranking genes based on *significance* of enrichment defined as: $-\log_{10}$(P-value)*Fold Change (Figure 6F; Tables S6A–S6C).

TF knockout paralog pairs

Previously identified specialized paralog pairs were assessed for expression changes in the context of knockout embryos. The aggregated median expression distribution of diapause and development genes in each pair in Knockout samples were compared to their median expression in both Scrambled and Wildtype together (control). Degree of specialization (difference between diapause gene and development gene median expression) was different in the context of *REST* but not *FOXO3a* and *FOXO3b* knockout (Figures 6G and S6G, two-way ANOVA, $P < 0.05$).

Lipidomics analysis

Principal Component Analysis (PCA) was performed using all the lipids identified for: 1) African turquoise killifish diapause and development samples (Figure 7B); and 2) African turquoise and red-striped killifish pre-diapause samples (Figure S7C). The total of 431 filtered and normalized lipid intensities were used for PCA (see below), which were also plotted using autoplot function in ggfortify package (version 0.4.11) in R (version 4.0.5).

Discriminant analysis was performed using a Welch's t-test that does not assume equal population variances for each lipid among the two diapause (6 days and 1 month) and the two development conditions (pre-diapause and diapause escape). Lipids that were significantly different (Welch's t-test, $P < 0.05$ after multiple hypothesis correction using Benjamini-Hochberg method) between diapause and development but did not significantly change between the two development conditions were categorized as diapause specific lipids. These constitute lipids that go up or down when embryos enter diapause but do not change among the two development time points. This led to 350 diapause specific lipid changes, 80 of which were triglycerides, including very long chain fatty acid triglycerides (Figures 7C, 7D, S7B, and S7C; Table S7C).

Quantification of lipid droplets

To visualize lipid droplets in *N. furzeri*, embryos were collected using from the same mating cohorts used to generate CRISPR/Cas9-mediated knockout embryos for experiments described above (1 male to 3 females, ~1.5-3 months of age). Viable embryos were washed with ~1ml of embryo solution and then monitored until they had reached the proper stages of development and diapause. Embryos were segregated into four groups: 1) embryos in pre-diapause state (date of heartbeat onset) (Pre-Dia), 2) embryos in development (1 day post-heartbeat onset, heartbeat > ~85 beats per minutes) (Dev), 3) embryos in early diapause (6 days post heartbeat onset, heartbeat slowed to < ~20 beats per minutes) (Dia (6d)), and 4) embryos in late diapause (1 month post heartbeat onset, heartbeat slowed to < ~20 beats per minute) (Dia (1m)). Embryos were processed to visualize lipid droplets by staining with the neutral lipid dye BODIPY™ 493/503 (D3992, Invitrogen). Embryos were dissected at 4°C in 1x phosphate buffered saline (PBS), removing the chorion and embryonic membrane so the embryo body could be isolated. Following dissection, embryos were placed in a 9-well

1ml glass plate (PYREX™ Spot plate), with each well containing 5-7 embryo for the same condition. Embryos were fixed in ∼1ml of freshly diluted 4% paraformaldehyde (PFA) (28906, Thermo Scientific) for 1 hour at room temperature, followed by three wash steps in ∼1ml PBS to remove residual PFA. To stain for lipid droplets, embryos were incubated for 30 minutes at room temperature in the dark with 1.5μg/ml BODIPY™ 493/503 (D3992, Invitrogen) in ∼1ml PBS. Embryos were washed with ∼1ml PBS once to remove residual dye, mounted on a 2% agarose pad and covered with a glass cover slide using spacers for imaging.

Supplemental figures

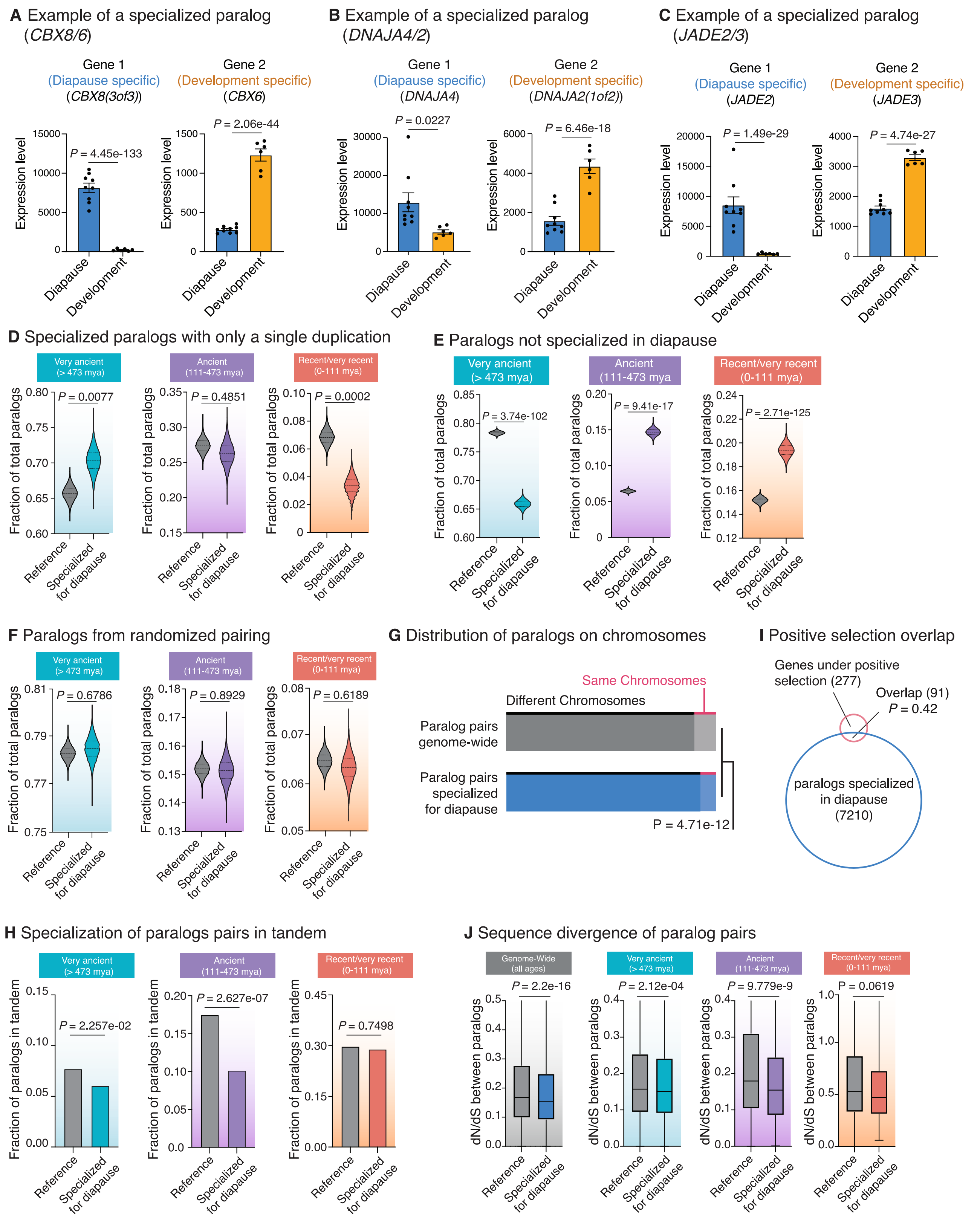

(legend on next page)

Figure S1. Evaluation of the diapause-specialized paralogs in the African turquoise killifish, related to Figure 1

(A–C) Examples of paralog gene pairs, with specialized expression of gene 1 in diapause (blue) and gene 2 in development (orange) in African turquoise killifish (*Nothobranchius furzeri*). Bars represent mean expression level (normalized DESeq2 count) across replicates in diapause or development state. Dots show normalized DESeq2 counts in each replicate. Error bar is standard error of mean. Corrected *p* values (median from pairwise comparisons) from DESeq2 Wald test.

(D) Fraction of total paralog pairs within each of the very ancient (left), ancient (middle), and recent/very recent (right) binned categories. Violin plots represent distribution of observed vs. expected specialized paralog fractions generated through 10,000 bootstrapped random sampling. Median and quartiles are indicated by dashed lines. Only paralogs that have experienced a single duplication event were included in this analysis. The enrichment of diapause-specialized paralog pairs within each bin is compared with genome-wide expectation (reference). Compared with the reference, paralogs with specialization in diapause are enriched among genes with very ancient duplication times and depleted among genes with ancient and recent or very recent duplication times, respectively, indicating that our results are not affected by gene family size. *p* values from chi-square test (see also Table S1D).

(E) Fraction of total paralog pairs within each of the very ancient (left), ancient (middle), and recent/very recent (right) binned categories. Violin plots represent distribution of observed vs. expected specialized paralog fractions generated through 10,000 bootstrapped random sampling. Median and quartiles are indicated by dashed lines. The enrichment of non-diapause-specialized paralog pairs within each bin is compared with genome-wide expectation (reference). Compared with the reference, paralogs with no specialization in diapause are depleted among genes with very ancient duplication times and enriched among genes with ancient and recent or very recent duplication times, respectively, suggesting that our results are specific to diapause-specialized paralogs (see also Table S1D).

(F) Fraction of total paralog pairs within each of the very ancient (left), ancient (middle), and recent/very recent (right) binned categories. Violin plots represent distribution of observed vs. expected specialized paralog fractions generated through 10,000 bootstrapped random sampling. Median and quartiles are indicated by dashed lines. Paralogs were randomized among each expression categorization grouping and compared with genome-wide expectation (reference). Compared with the reference, paralogs with randomized expression status show no significant enrichment or depletion among any duplication time, suggesting that enrichment for ancient paralogs is not expected by chance (see also Table S1D).

(G) Genomic distribution of all paralog pairs and of paralog pairs specialized in diapause. The majority of paralog pairs are chromosomal duplications. Paralog pairs on separate chromosomes are also more likely to specialize for diapause than the genome averages. *p* values were calculated using Mann-Whitney U test.

(H) Assessment of specialization status of tandem duplicates (paralogs on the same chromosomes). Tandem duplicates were significantly less likely to specialize for diapause compared with their genome averages for all the three duplication time categories. *p* values were calculated using Mann-Whitney U test.

(I) The overlap between paralogs specialized for diapause in the African turquoise killifish (blue circle) and genes that showed a signature of positive selection at the level of protein sequence (red circle). $p = 0.121$, hypergeometric test.

(J) Sequence divergence of the paralog pairs specialized for diapause. Sequence divergence (dN/dS) was calculated using Yang-Neilson method between each paralog pair genome wide followed by sub-setting for each duplication time category. Paralogs that are specialized for diapause are more conserved at the gene sequence level compared with reference (respective genome averages) for all the three duplication time category, as well as genome-wide comparison. *p* values were calculated using Mann-Whitney U test.

CellPress
OPEN ACCESS

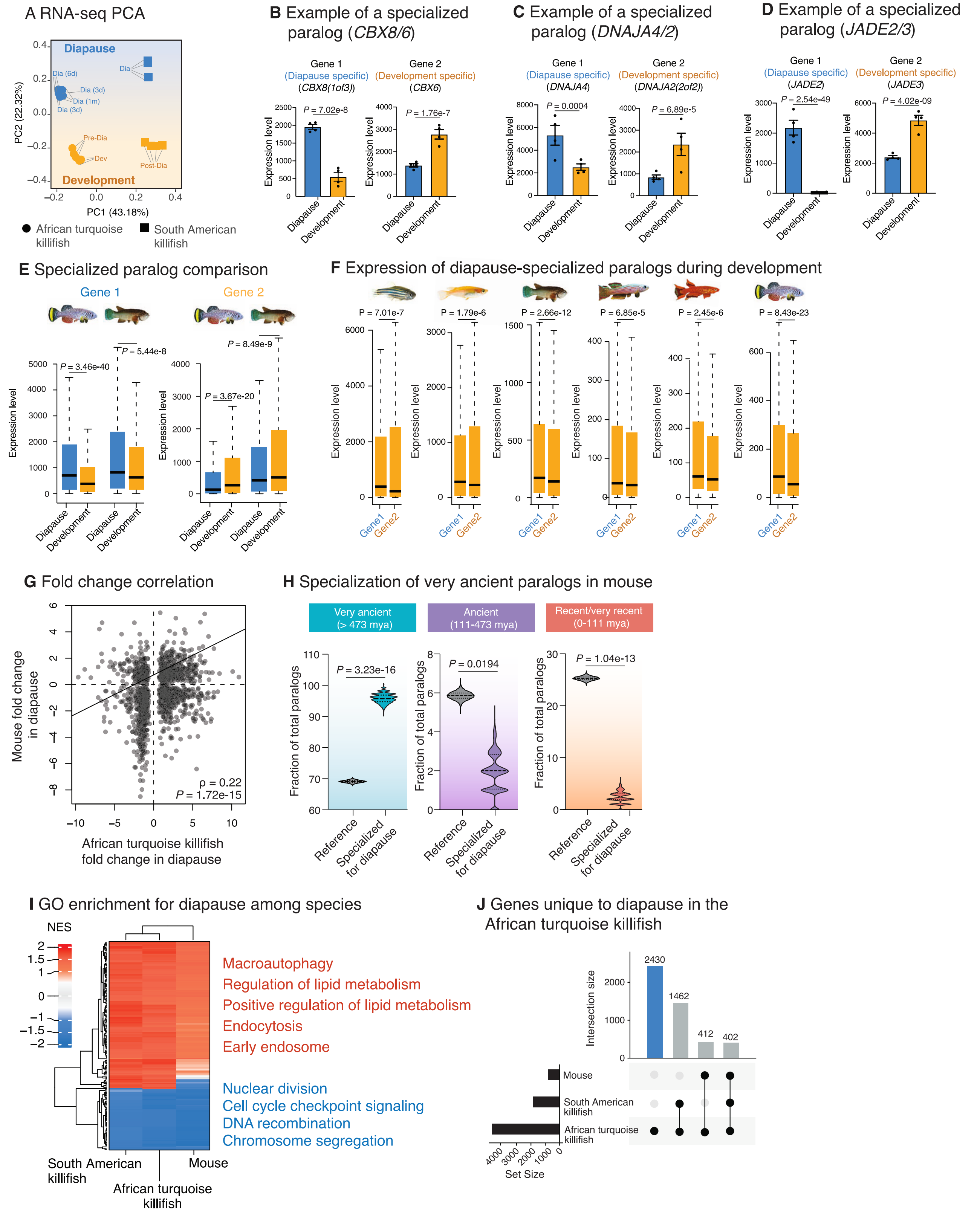

(legend on next page)

Figure S2. Comparison of diapause gene expression and specialized paralogs in African turquoise killifish, South American killifish, and additional fish species, related to Figure 2

(A) Principal-component analysis of RNA-seq libraries during diapause (blue) and development (orange) in African turquoise killifish (circles) and South American killifish (squares). Development samples in the South American killifish are from 4 days post-diapause development stage. PC1 separates samples by species, and PC2 separates samples by stage, suggesting that the diapause and development stages across species are comparable. Each dot represents an RNA-seq replicate library for a given species and stage.

(B–D) Examples of paralog gene pairs, with specialized expression of gene 1 in diapause (blue) and gene 2 in development (orange) in the South American killifish (*Austrofundulus limnaeus*). Displayed gene names are the assigned name of relevant ortholog in African turquoise killifish for comparison. Bars represent mean expression level (normalized DESeq2 count) across replicates in diapause or development state. Dots show normalized DESeq2 counts in each replicate. Error bar is standard error of mean. *p* values from DESeq2 Wald test.

(E) Comparison of diapause-specialized paralogs identified in the African turquoise killifish to their orthologs in the South American killifish. Both the diapause-specialized genes (gene 1 cohort; left) and the development-specialized gene (gene 2 cohort; right) exhibit the same expression pattern genome wide in both the fish species. These expression differences were significant in both African turquoise killifish and South American killifish. *p* values from Kolmogorov-Smirnov test.

(F) Expression of African turquoise killifish diapause-specialized paralogs and their one-to-one orthologs in other species. Expression was evaluated during pre-diapause development, and *p* values were calculated using Kolmogorov-Smirnov test. In all species evaluated, the expression pattern was similar at the comparable pre-diapause developmental time point with the diapause-specific gene (gene 1) always the more highly expressed during the pre-diapause developmental time point. This expression asymmetry is a known property of paralogs.[16]

(G) Spearman's rank correlation between ortholog genes that change with diapause in African turquoise killifish and mouse. Dots represent the fold change values of ortholog genes in diapause compared with development in the two species. Spearman's correlation coefficient (ρ) and *p* values are indicated.

(H) Fraction of total mouse paralog pairs within each of the very ancient (left), ancient (middle), and recent/very recent (right) binned categories. Violin plots represent the distribution of observed vs. expected specialized paralog fractions generated through 10,000 bootstrapped random sampling. Median and quartiles are indicated by dashed lines. The enrichment of diapause-specialized paralog pairs within each bin is compared with genome-wide expectation (see STAR Methods). Compared with the reference, paralogs with specialization in diapause are enriched among genes with very ancient duplication times and depleted among genes with ancient and recent/very recent duplication times, respectively. *p* values from chi-square test.

(I) Gene Ontology (GO) functions shared between diapause in African turquoise killifish and South American killifish or mouse (see also Table S2A).

(J) Upset plot depicting genes that are differentially expressed in diapause only in the African turquoise killifish compared with diapause in South American killifish and diapause in the mouse. The final set of unique genes used for downstream analysis is composed of 2,430 genes (blue histogram bin).

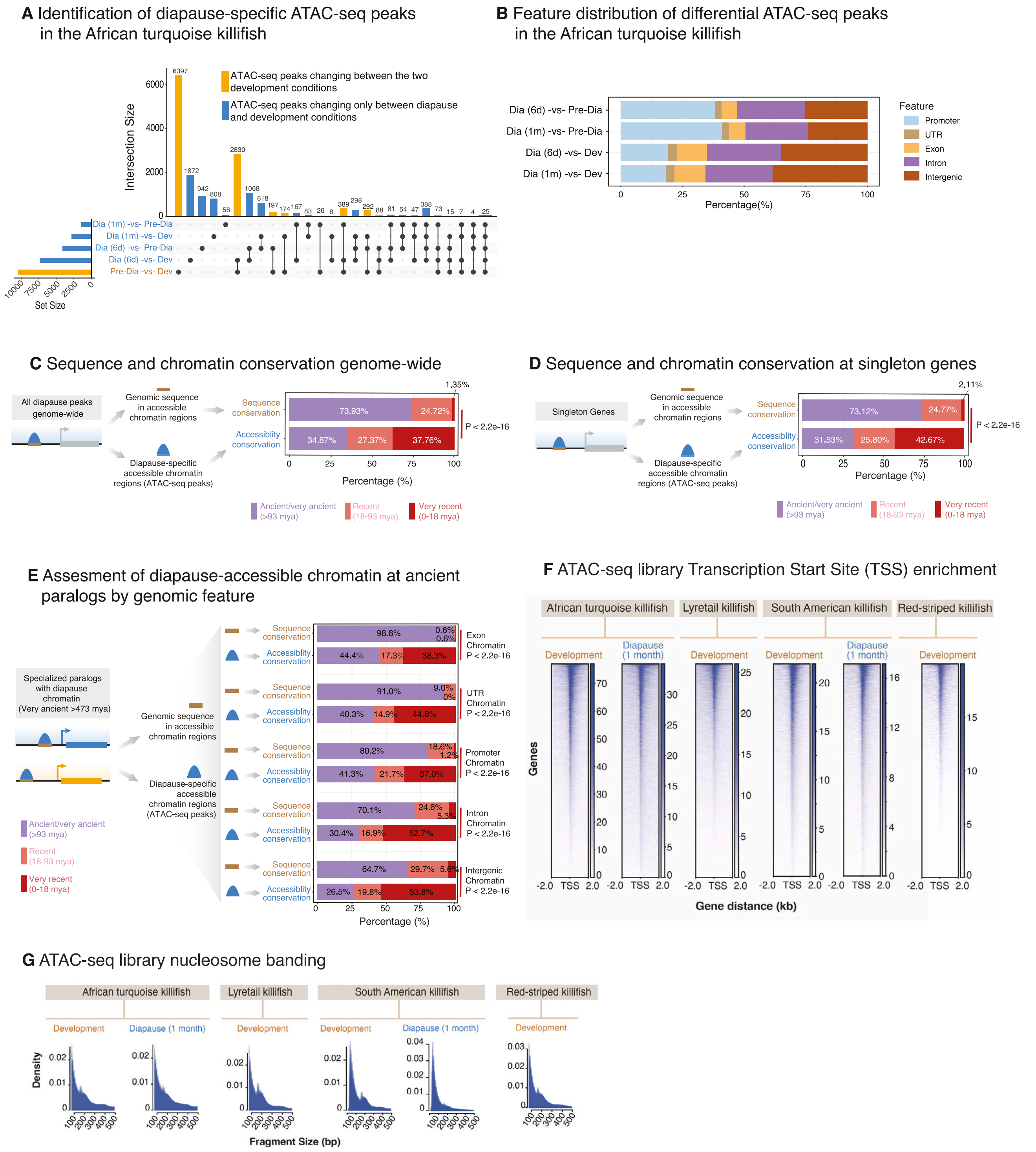

Figure S3. Characterization of ATAC-seq datasets in the African turquoise killifish, related to Figure 3

(A) Upset plot depicting differentially accessible chromatin regions (ATAC-seq peaks) between the consensus peak set for each biological time point surveyed in the African turquoise killifish. The final set of differentially accessible chromatin regions used for analysis is composed of all intersections containing peaks that only change between diapause and development conditions (blue histogram bins), while those that include a change between developmental time points were excluded (orange histogram bins).

(B) Percentage breakdowns of included diapause-specific, differentially accessible chromatin regions by genome feature in the African turquoise killifish. Feature categories (promoter, UTR, exon, intron, and intergenic) were made by consolidating specific regions.

(legend continued on next page)

(C) Conservation analysis of genomic sequence and chromatin accessibility genome wide for all the significant diapause-specific chromatin peaks (see Figure 3D for paralog-specific result). Left: schematic of the analysis. Right: percentage (e.g., conservation) of alignable regions containing diapause-specific chromatin accessibility (upper) and the conservation of diapause-specific chromatin accessibility (lower) genome wide.
(D) Conservation analysis of genomic sequence and chromatin accessibility for all the significant diapause-specific chromatin peaks at singleton genes (genes without paralogs). Left: schematic of the analysis. Right: percentage (e.g., conservation) of alignable regions containing diapause-specific chromatin and the conservation of diapause-specific chromatin accessibility at those sites.
(E) Conservation analysis of genomic sequence and chromatin accessibility at very ancient paralogs with specialization in diapause vs. development delineated by genomic feature in order of decreasing sequence conservation: accessible chromatin in exons (upper pair), untranslated regions (UTRs) (upper-middle pair), promoters (middle pair), introns (middle-lower pair), and intergenic regions (lower pair). Left: schematic of the analysis. Right: percentage (e.g., conservation) of alignable regions containing diapause-specific chromatin accessibility (upper) and the conservation of diapause-specific chromatin accessibility (lower) near specialized ancient paralogs.
(F) Representative plots for read enrichment at TSS and neighboring 2 kb regions for selected ATAC-seq libraries for each species. An enrichment of accessibility signal at TSS indicates good quality.
(G) Representative nucleosome banding patten displaying the presence/absence and intensity of the mono-, di-, and tri-nucleosome bands for selected ATAC-seq libraries.

CellPress
OPEN ACCESS

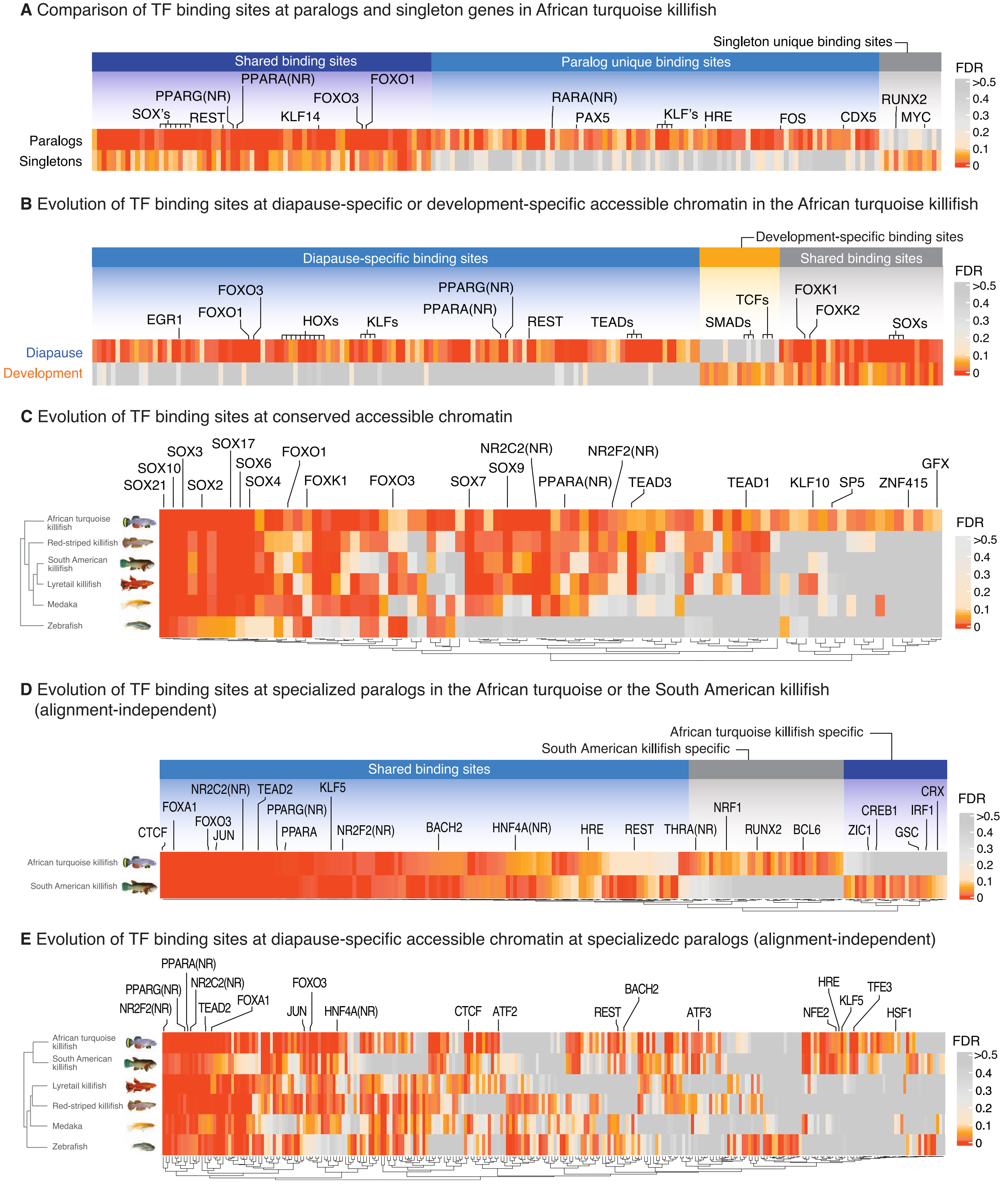

Figure S4. Transcription factor binding sites in accessible chromatin during diapause, related to Figure 4

(A) Comparison of transcription factor binding sites enriched in diapause-specific chromatin peaks closest to either specialized paralog or singleton genes (genes without paralogs). Many TF binding motifs are enriched specifically in diapause-specific chromatin at paralogs. Selected representative binding sites are highlighted.

(legend continued on next page)

(B) Evolution of transcription factor binding sites at diapause-specific or development-specific chromatin in the African turquoise killifish. The majority of enriched binding sites are specific to the diapause-accessible chromatin. Selected representative binding sites are highlighted.

(C) Evolution of transcription-factor binding sites in regions where chromatin accessibility is conserved across all fish species. Chromatin accessibility sites in the African turquoise killifish were included if they were conserved in at least one other killifish species without diapause and at least one outgroup fish species. Many transcription factor binding sites are enriched across all species and differ from the diapause-specific enrichment in the African turquoise killifish seen in Figure 4B. Selected representative motifs are highlighted.

(D) Evolution of transcription-factor binding sites enriched near specialized paralogs in the African turquoise killifish or the South American killifish (alignment-independent). Specialized paralogs were identified independently in both the species, and closest diapause-specific peaks were included. Majority of the binding sites are shared across the two species, suggesting that similar binding sites have evolved independently for diapause in these two species. Selected representative binding sites are highlighted.

(E) Evolution of transcription-factor binding sites at diapause-specific chromatin peaks at specialized paralogs in an alignment-independent manner. Diapause-specific chromatin peaks at specialized paralogs in either the African turquoise or the South American killifish were compared with all the peaks at their ortholog genes in other species after down sampling to the same peak numbers as the African turquoise killifish, preserving the distribution of peak type (e.g., promoter, intron, intergenic, etc.). Selected representative binding sites are highlighted.

CellPress
OPEN ACCESS

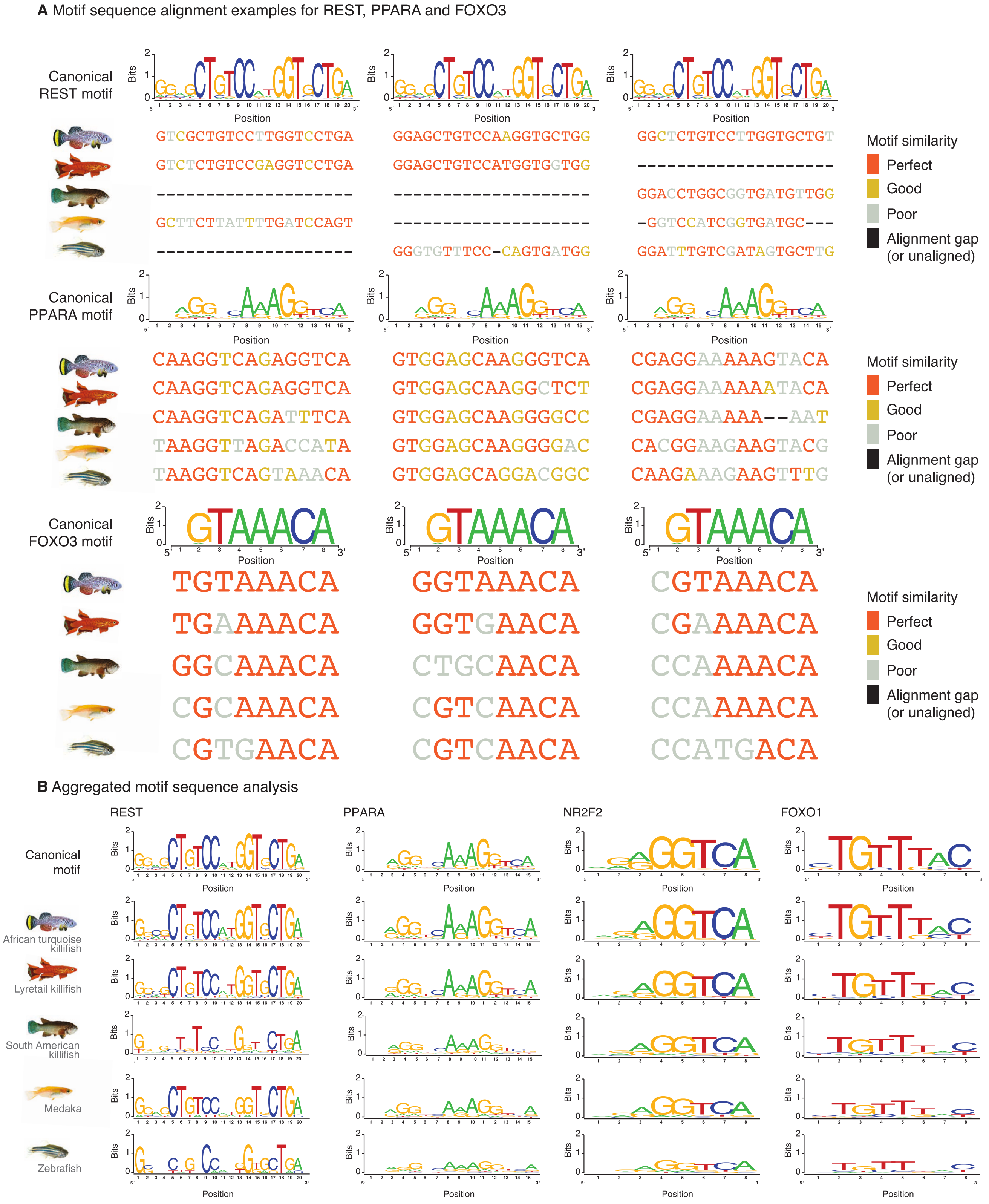

(legend on next page)

Figure S5. Additional examples and aggregated motifs from the motif evolution analysis, related to Figures 5 and 6

(A) Representative examples of REST (upper), PPARA (middle), and FOXO3 (lower) transcription factor binding sites in the African turquoise killifish and the aligned regions in other fish species. Aligned sequences colored in accordance with their closeness of fit to the information content of HOMER-produced consensus motif logo (top track). Only a single sequence is provided for both lyretail killifish and red-striped killifish, as they are aligned to the same lyretail killifish genome sequence.

(B) Aggregated informational content (bits) across all REST (left), PPARA (left-center), NR2F2 (right-center), and FOXO1 (right) transcription factor binding sites in diapause-accessible (differential) chromatin and aligned regions in other species regardless of accessibility status. The canonical motif logos are provided for comparison (upper logo). During sequence aggregation, sequence aligned to gaps were removed.

CellPress
OPEN ACCESS

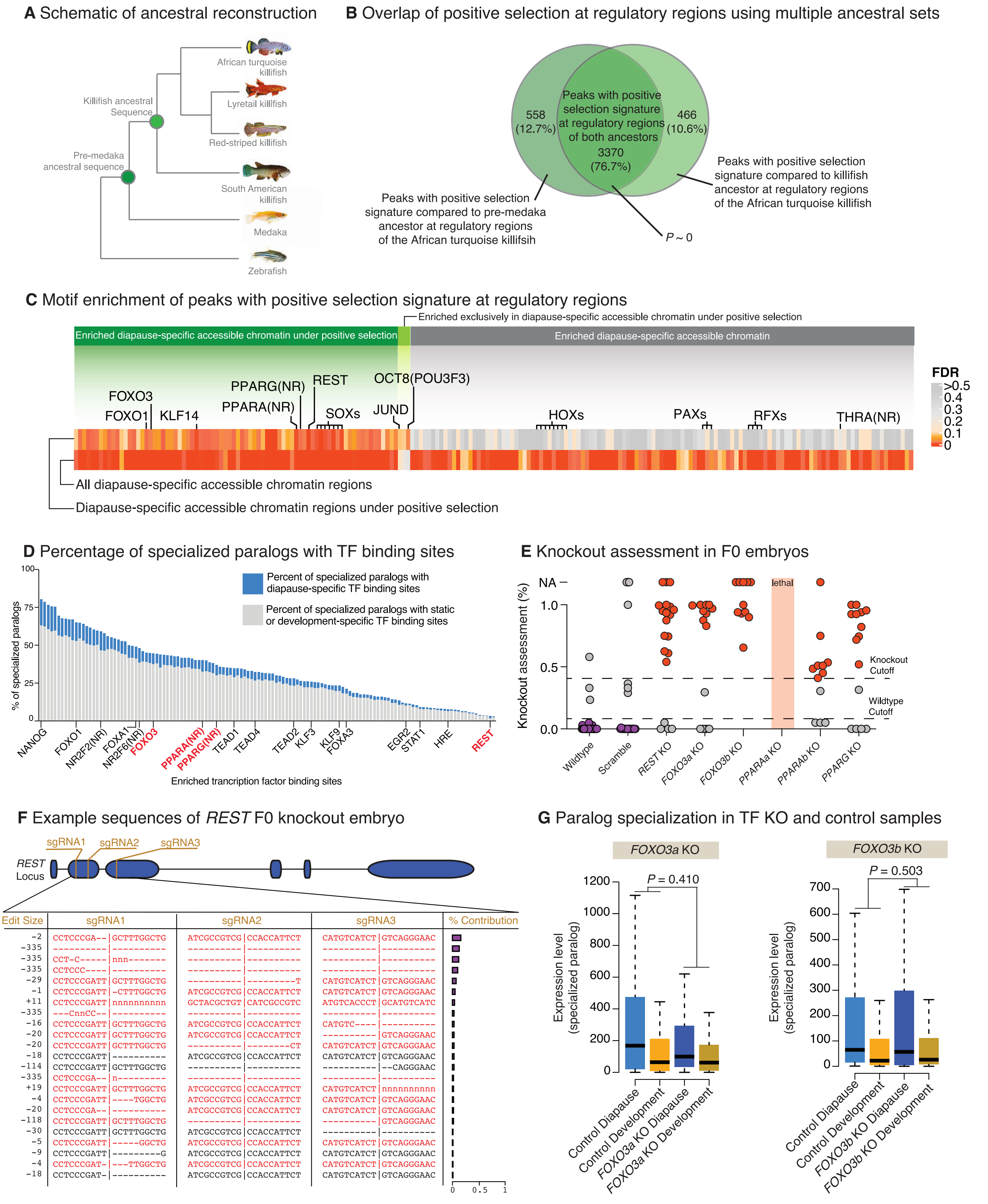

(legend on next page)

Figure S6. Positive selection analysis of motifs and CRISPR-Cas9-based knockout in F0 embryos, related to Figure 6

(A) Schematic tree showing the evolutionary timing of inferred ancestral sequences used for positive selection analysis on accessible chromatin regions. The green dots represent the inferred pre-medaka and killifish common ancestral sequences. The ancestral sequences were constructed using aligned sequences from each species in the tree with the site of green dots delineating the branches of the phylogeny classified as in-group and out-group, respectively (see STAR Methods).

(B) The overlap between peaks with a positive selection signature as calculated using the inferred pre-medaka (left) and killifish (right) ancestral sequence, respectively. The overlap between the two was significant ($p \sim 0$, hypergeometric test). We used the union of the two sets for determining the positive selection signature overlap with diapause-accessible (differential) chromatin near ancient, specialized paralogs (Figure 5E).

(C) Enrichment of transcription factor binding motifs among diapause-accessible chromatin peaks near ancient, specialized paralogs with a positive selection signature. Motifs such as REST, FOXO, and PPARA are significantly enriched in the positively selected chromatin regions.

(D) Percentage of specialized paralog pairs targeted by different transcription factors via chromatin accessibility (ATAC-seq). Each histogram represents the percentage of all specialized paralog pairs that have a chromatin accessibility site containing a given transcription factor binding site. Each histogram is also broken into the percentage of diapause-specific accessible chromatin containing the transcription factor binding sites (blue) and the percentage of development-specific/static accessible chromatin containing the transcription factor binding sites (gray). Representative transcription factors are labeled. Transcription factors used for functional evaluation by CRISPR-Cas9-mediated are highlighted in red.

(E) Predicted knockout scores generated by Synthego ICE Analysis. These scores were used to define thresholds for library inclusion for controls wild type (non-injected) or scramble (scrambled sgRNAs) (purple, <5% prediction score) and knockout (red, >45% knockout score). Libraries not meeting these criteria or excluded at other filtering steps are denoted in light gray. Samples for which no score could be generated are displayed at the top of the y axis. Note that *PPARAa* knockout is embryonic lethal, and no scores could be generated.

(F) Example of Sanger sequences from *REST* F0 knockout embryos. Schematic (top, exons in purple, introns in black) depicts the *REST* locus and denotes the three sites targeted by sgRNAs. The sequences below show the cut site (denoted with "|") and the ten bases directly upstream and downstream. The predicted indel is shown to the left of each sequence, and the individual sequence's contribution to the embryo lysate is shown as a histogram to the right. Each sequence resulting in a predicted frameshift is colored in red.

(G) Paralog specialization in diapause and development after *FOXO3a* and *FOXO3b* knockout. Differential expression between pair specialized for diapause and development (light blue/orange) is not significantly reduced in the context of *FOXO3a* and *FOXO3b* knockout (dark blue/orange) compared with control (median expression in both wild-type and scramble samples). (p values are from two-way ANOVA.)

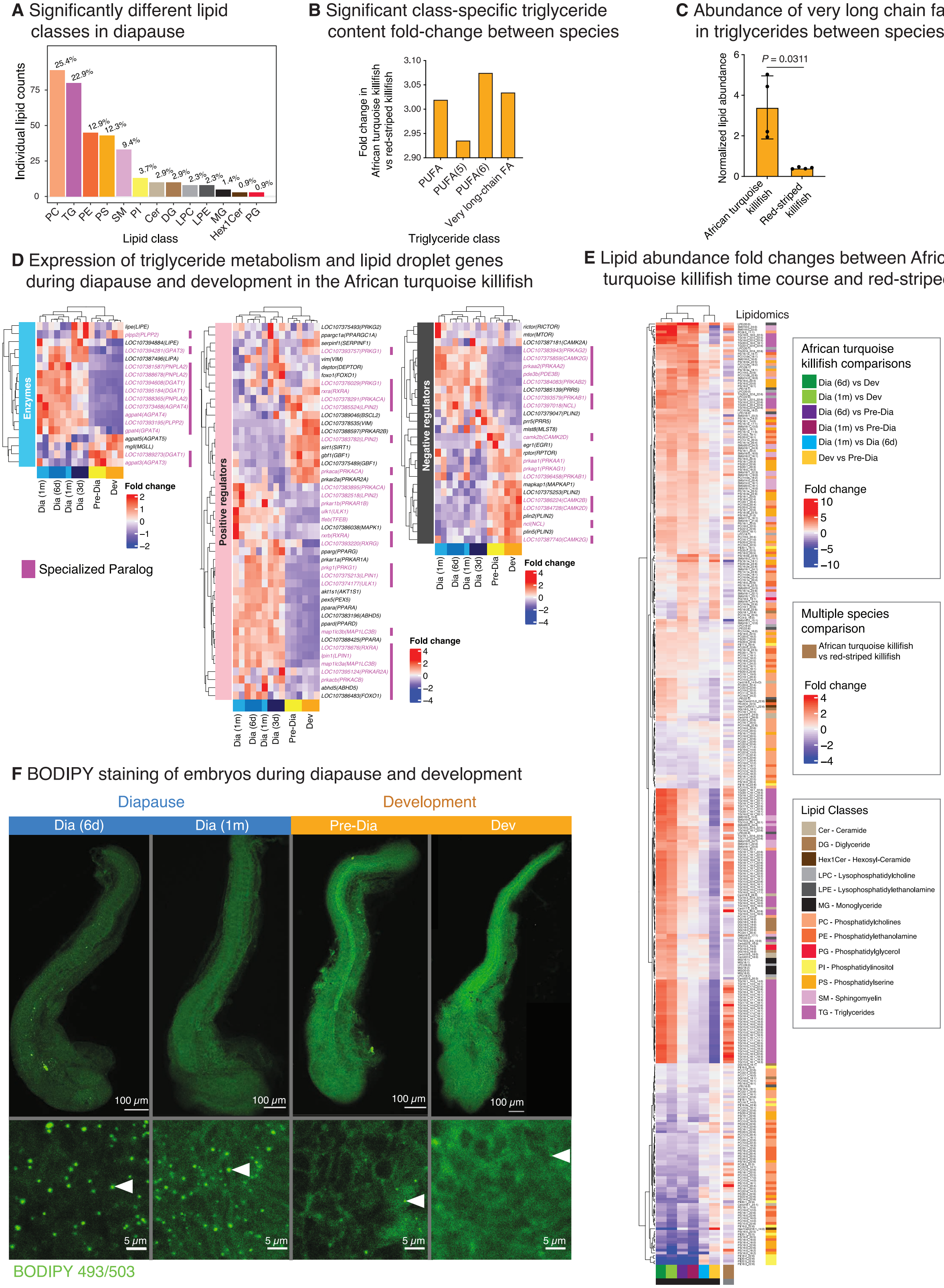

(legend on next page)

Figure S7. Analysis of TGs in the African turquoise killifish and lyretail killifish and analysis of lipid droplets in the African turquoise killifish, related to Figure 7

(A) Bar graph representing the number of diapause-specific differential lipids in each lipid class. Phosphatidylcholines (PCs) and triglycerides (TGs) constitute most of the differential lipids that change in diapause. PE, phosphatidylethanolamine; PS, phosphatidylserine; SM, sphingomyelin; PI, phosphatidylinositol; Cer, ceramide; DG, diglyceride; LPC, lysophosphatidylcholine; LPE, lysophosphatidylethanolamine; MG, monoradylglyceride; Hex1Cer, hexosyl-ceramide; PG, phosphatidylglycerol.

(B) Comparison between triglyceride subclass levels between the African turquoise killifish and the red-striped killifish, shown as fold change in total lipid abundance. All triglycerides belonging to each class (poly-unsaturated fatty acids [PUFAs] cumulatively or with specifically with five [5] or six [6] unsaturated/double-bond sites, respectively; very-long-chain FA, long-chain fatty acids that contain >21 carbons) were summed for this analysis (see STAR Methods). The same developmental stage corresponding to the pre-diapause stage in the African turquoise killifish was compared between the two species. The African turquoise killifish has a higher TG content at the pre-diapause stage compared with the red-striped killifish.

(C) Normalized lipid abundance counts for very-long-chain triglycerides in the African turquoise killifish (left) and red-striped killifish (right) during matched developmental time points (Pre-Dia). Data represented as in (F). *p* value from Welch's t test.

(D) RNA-seq expression levels of the genes involved in triglyceride and lipid droplets (LDs) divided by their functions: enzymes (left heatmap), positive regulators of triglyceride metabolism and LD formation (middle heatmap), and negative regulators of triglyceride or LD metabolism (right heatmap). Many enzymes, positive and negative regulators of TG and LD metabolism, were differentially regulated during diapause or development. Genes labeled in magenta are members of diapause-development-specialized paralog pairs. Many of them also show differential regulation during diapause and development, suggesting potential specialization of TG and LD metabolism for diapause or development.

(E) Heatmap representing the fold change of all significant lipids species between diapause vs. development in the African turquoise killifish (left) and between the African turquoise killifish vs. red-striped killifish (development only, rightmost). Fold change values are plotted between each pairwise comparison between diapause and development time points, or the two development time points. Lipids were included if significance was reached in any single comparison. The rightmost panel shows the fold change values of the same lipids in the African turquoise killifish compared with the red-striped killifish.

(F) Representative images of BODIPY 493/503 staining in whole, dissected embryos using a 5× (upper) and 20× (lower) objective. Scale bars represent 100 and 5 μm distance in each 5× and 20 image, respectively. White arrows highlight a single lipid droplet stained by BODIPY. 6 days in diapause (left), 1 month in diapause (center-left), pre-diapause (center-right), and development (right) time points were imaged in one experiment.
